# Synthesis of novel carbohydrate based pyridinium ionic liquids and cytotoxicity of ionic liquids for mammalian cells†

**DOI:** 10.1039/d0ra01370f

**Published:** 2020-04-07

**Authors:** Melanie Reiß, Andreas Brietzke, Thomas Eickner, Florian Stein, Alexander Villinger, Christian Vogel, Udo Kragl, Stefan Jopp

**Affiliations:** Institute of Chemistry, University of Rostock Albert-Einstein-Straße 3a 18059 Rostock Germany stefan.jopp@uni-rostock.de; Institute of Biomedical Engineering, University of Rostock Friedrich-Barnewitz-Straße 4 18119 Rostock Germany

## Abstract

The large pool of naturally occurring carbohydrates with their diversity in chirality and structure led to the idea of a systematic investigation of carbohydrate based ILs. To this end, we investigated the influence of different ether groups, mainly methyl or ethyl ether, on the secondary OH groups as well as different configurations on physical properties such as melting point, thermostability and especially the influence on cell toxicity. For this investigation we chose α- and β-methyl-, β-allyl- and β-phenyl d-glucopyranose as well as four 1-deoxy-pentoses. In order to be able to classify the results, more ionic liquids with different structural motives were examined for cytotoxicity. Here, we present data that confirm the biocompatibility of such ILs consisting of naturally occurring molecules or their derivatives. The synthesized carbohydrate based ILs were tested for their suitability as additives in coatings for medical applications such as drug-eluting balloons.

## Abstract 2

The large pool of naturally occurring carbohydrates with their diversity in chirality and structure led to the idea of a systematic investigation of carbohydrate based ILs.

## Graphical abstract

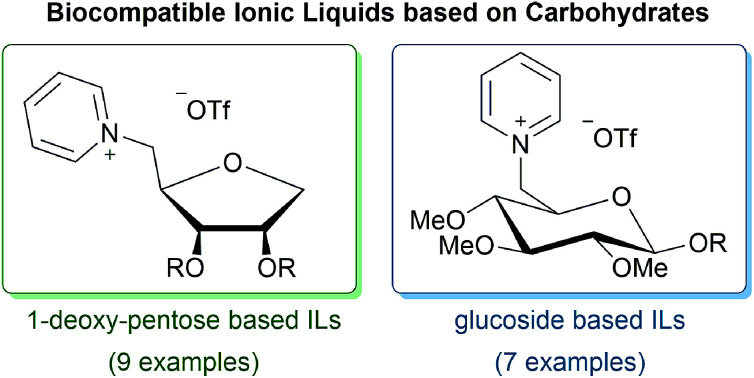

## Introduction

In recent years, ionic liquids have aroused the interest of scientists in the medical research field due to their wide variety of structures and potential applications.^1^ Current areas of investigation include IL based drugs (Active Pharmaceutical Ingredients-Ionic Liquids)^2^ as well as other applications of ILs in the pharmacological field such as drug delivery systems.^1,3^ In such systems, the IL serves as a solvent or solvent promoter for sparingly water-soluble drugs and can thus enhance pharmacokinetic and pharmacodynamic properties considerably.

Another interesting medical application are drug-eluting balloons, which are typically used in re-stenotic lesions.^4^ Previous works of our group have shown that the ionic liquid cetylpyridinium salicate [CetPyr][Sal] is a potential candidate for such an application.^5^ This IL however, while showing good properties in drug release and homogenous coating ability, is not suited for an actual application due to cytotoxicity.

**Fig. 1:**
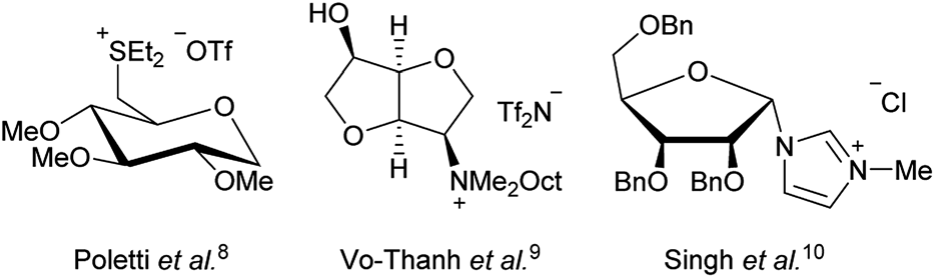
Examples of carbohydrate based ILs from the literature.

Thus we decided to further look into ILs based on natural products like amino acids^6^ and carbohydrates^7^ Especially carbohydrate based ionic liquids have piqued our interest as potential ILs with low cytotoxicity. While there have been a few previous studies on carbohydrate based ILs,^7–10^ most of them focused on the synthesis. Examples of previous ILs based on carbohydrates are shown in Fig. 1.

While the use of some of these carbohydrate based ILs as solvents or for asymmetric induction^9^ has been studied, a study for potential medical application has yet to be done.

## Results and discussion

### Synthesis

**Fig. 2:**
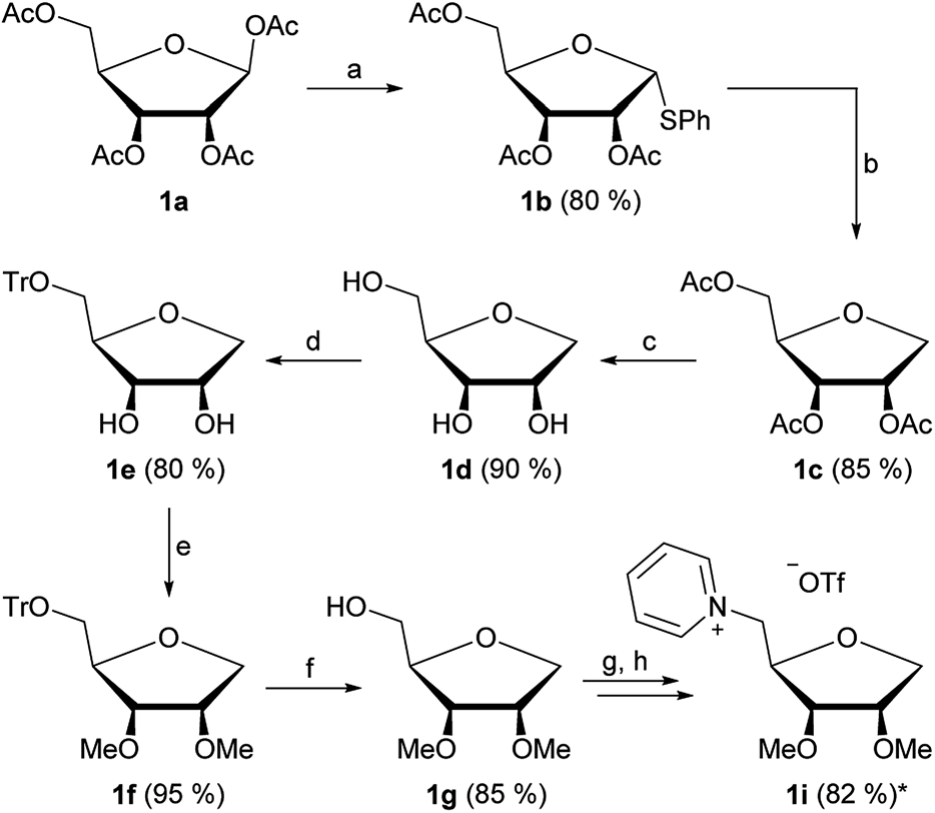
Synthesis of 1-deoxy-ribose based ionic liquid 1i; the same synthetic strategy was applied for lyxose (2a–i), xylose (3a–i) and arabinose (4a–i). (a) Thiophenol, BF_3_·OEt_2_, DCM, 0 °C ≥ r.t., 2 h; (b) Bu_3_SnH, AIBN, toluene, reflux, 2.5 h; (c) MeOH, Na, r.t., 4 h; (d) trityl chloride, NEt_3_, DMAP, DCM, r.t., overnight; (e) NaH, MeI, DMF, 0 °C ≥ r.t., overnight; (f) AcOH (70%), 70 °C, 45 min; (g) Tf_2_O, pyridine, DCM, 0 °C, 10 min; (h) pyridine, reaction at rotary evaporator, 700 mbar, 40 °C; *yield over two steps.

The first part of our strategy was the synthesis of four new ionic liquids derived from pentoses. We chose the peracetylated species of d-ribose 1a, d-lyxose 2a, d-xylose 3a and l-arabinose 4a as starting materials, which were prepared by procedures known from literature.^11^Fig. 2 shows the general synthetic pathway exemplary for d-ribose.

Starting from the peracetylated d-ribose 1a we investigated a direct reduction of the anomeric center using TMSOTf and Et_3_SiH.^12^ While this strategy successfully led to the 1-deoxy-ribose derivative 1c in a high yield of 92%, it could not be applied for the other three starting materials 2–4a. The yields were low and the products impure. Thus we switched to a two-step strategy of first introducing a thiophenyl group at the anomeric center followed by reduction with tributyltin hydride (Fig. 2a and b). This strategy could be applied for all four products in yields from 50 to 85%.

The next four steps were the deprotection of the acetyl groups, 5-*O*-tritylation, introduction of the methyl ether groups on the secondary OH groups and lastly deprotection of the 5-*O*-trityl group, leading to the products 1–4g (Fig. 2c–f). These steps were generally performed with high yields ranging from 80 to 95%.

Our strategy was finalized by converting the unprotected 5-OH group into a triflate, directly followed by the quarternization reaction with pyridine, yielding the products 1–4i in overall high yields after an 8-step synthesis (Fig. 2g and h).

By using 1d and 1e as starting materials in a similar pathway as shown in Fig. 1 we were furthermore able to produce four additional 1-deoxy-ribose based ILs with varying groups in positions 2 and 3.

**Fig. 3:**
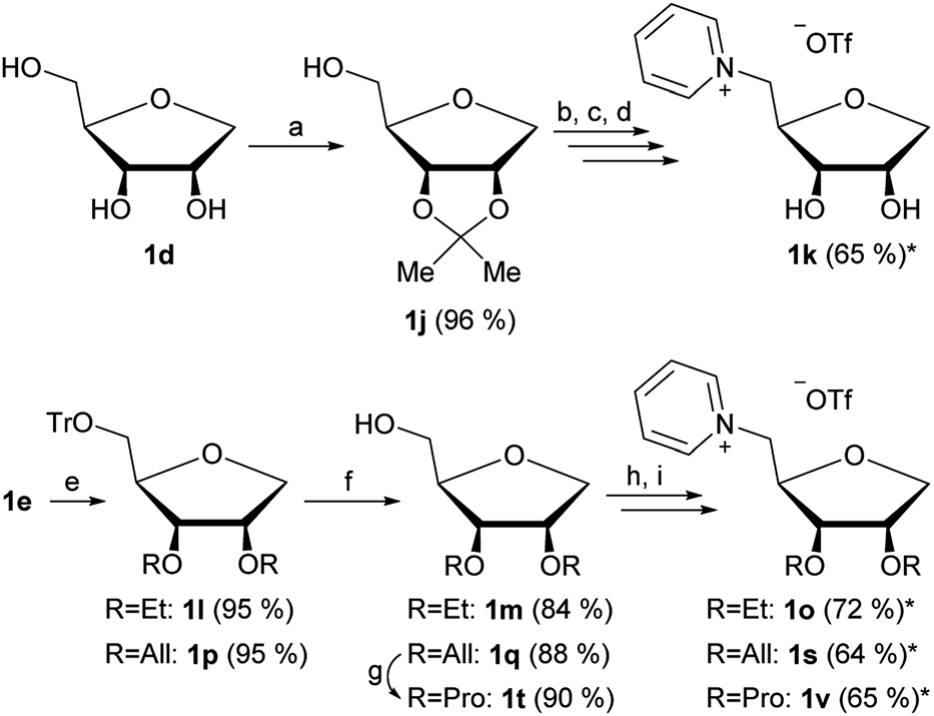
Synthesis of alternative 1-deoxy-ribose ILs with varying 2- and 3-*O*-groups. (a) 2,2-Dimethoxy propane, camphorsulfonic acid, acetone, r.t., 1.5 h; (b) Tf_2_O, pyridine, DCM, 0 °C, 10 min; (c) pyridine, reaction at rotary evaporator, 700 mbar, 40 °C; (d) AcOH (70%), r.t., 20 min; (e) NaH, ethyl or allyl bromide, DMF, 0 °C ≥ r.t., overnight; (f) AcOH (70%), 70 °C, 45 min; (g) Pd(OH)_2_, MeOH, H_2_-atmosphere, r.t., 12 h; (h) Tf_2_O, pyridine, DCM, 0 °C, 10 min; (i) pyridine, reaction at rotary evaporator, 700 mbar, 40 °C; *yield over two steps.

**Fig. 4:**
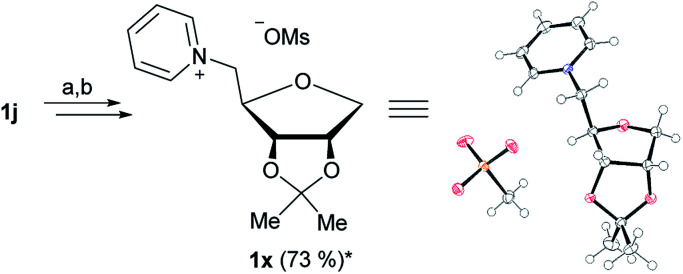
Synthesis and ORTEP^13^ of 1x. (a) Mesyl chloride, pyridine, r.t., 12 h; (b) pyridine, 125 °C, 5 h; *yield over two steps.

The isopropylidene protection of 1d (Fig. 3a) allows the production of ionic liquid 1k, which has free OH groups in the positions 2 and 3. Due to the instability of the isopropylidene group under acidic conditions, the synthesis of an isopropylidene protected 1-deoxy-ribose based IL was not possible at first. Said group was partly cleaved during the introduction of the 5-*O*-triflate group, thus a full cleavage of the isopropylidene group was performed after quarternization (Fig. 3b–d). We were however able to produce the isopropylidene protected 1-deoxy-ribose based IL 1x by using mesylate instead of triflate (Fig. 4).

By changing the reagent used for the introduction of the ether groups starting from 1e, ethyl and allyl ethers 1l and 1p have also been synthesized successfully (Fig. 3e). By further reducing the allyl group to a propyl group and carrying on with the already established strategy of introduction of the triflate followed by quarternization with pyridine, products 1o, 1s and 1v were achieved.

**Fig. 5:**
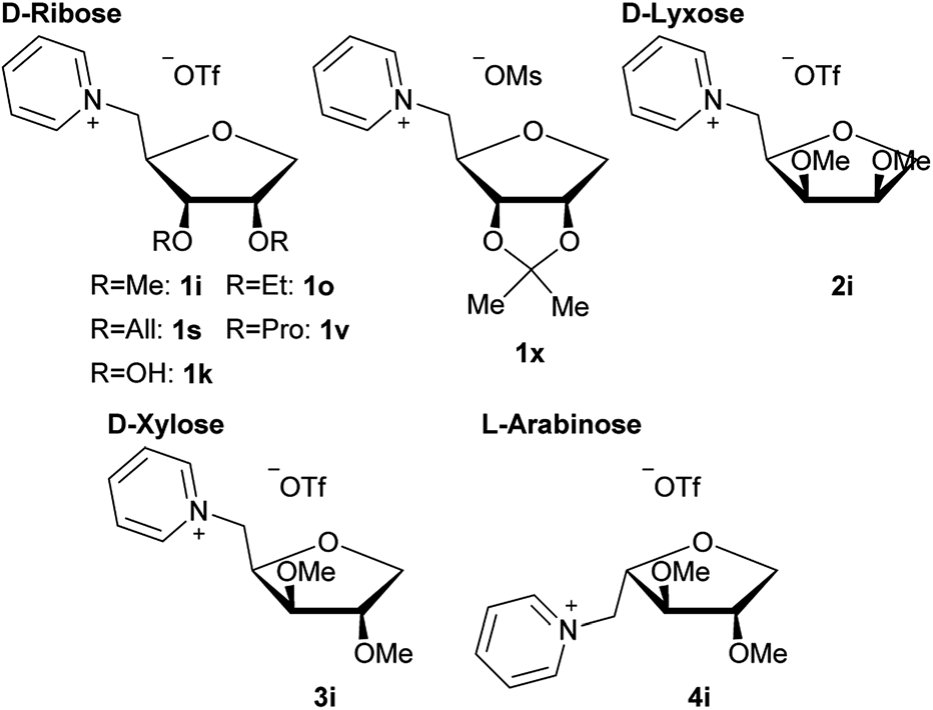
Overview of 1-deoxy-pentose based ILs.

All nine new pyridine salts derived from pentoses are shown in Fig. 5. These ionic structures all classify as ionic liquids, as further shown under “Thermal analysis”.

**Fig. 6:**
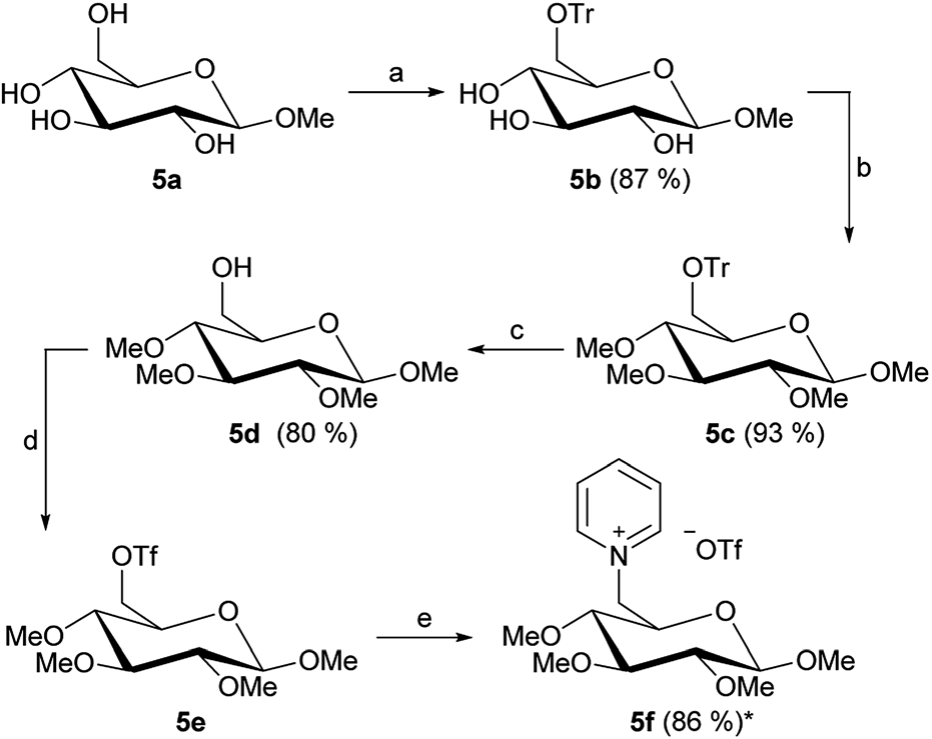
Synthesis of ionic liquid 5f from β-d-methyl glucoside 5a; the same strategy was applied for β-allyl glucoside (6a–f), β-phenyl glucoside (7a–f) and α-methyl glucoside (8a–f). (a) Trityl chloride, NEt_3_, DMAP, DCM, r.t., overnight; (b) NaH, MeI, DMF, 0 °C ≥ r.t., overnight; (c) AcOH (70%), 70 °C, 45 min; (d) Tf_2_O, pyridine, DCM, 0 °C, 10 min; (e) pyridine, reaction at rotary evaporator, 700 mbar, 40 °C; *yield over two steps.

Our second synthetic goal was to apply the established strategy for 1-deoxy-pentoses on different glucosides. Our starting materials were β-d-methyl, allyl and phenyl glucosides 5–7a as well as α-d-methyl glucoside 8a. Fig. 6 shows the general synthetic pathway exemplary for the β-d-methyl glucoside 5a.

The 5-step reaction starts with the introduction of the 6-*O*-trityl group (Fig. 6a). This step was performed with yields from 66% when using the β-d-allyl glucoside up to 87% for the β-d-methyl glucoside. The follow-up reactions are, similar to the synthesis shown in Fig. 2, the introduction of the methyl ether groups and afterwards the 6-*O*-trityl deprotection. These two steps were generally performed in high yields up to 94% (Fig. 6b and c). The resulting free 6-OH group of the products 5–8d was then converted into a triflate group followed by quarternization with pyridine, leading to the carbohydrate-based pyridinium triflate salts 5–8f (Fig. 6d and e).

**Fig. 7:**
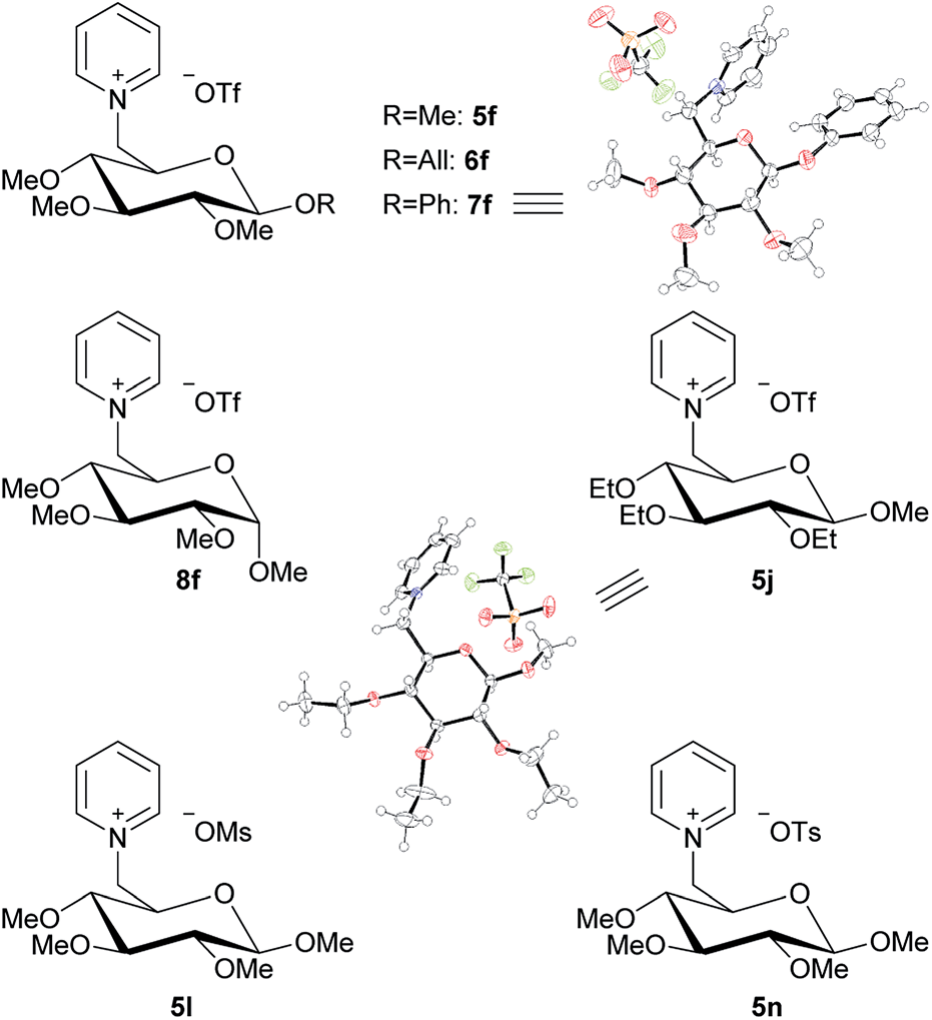
Overview of glucoside-based pyridinium salts and ORTEPs^13^ of 7f and 5j.

By following the same idea as applied before for the 1-deoxy-pentoses, the methyl ether groups were also changed to ethyl ether groups on the β-d-methyl glucoside structure. This leads to product 5j (Fig. 7). Lastly, two further salts were derived from 5d by changing the leaving group to mesylate or tosylate, leading to 5l and 5n, respectively (Fig. 7).

All seven new pyridine salts derived from glucosides are shown in Fig. 7. Most of these new products classify as ionic liquids, as further shown under “Thermal analysis”.

### Biocompatibility

**Fig. 8:**
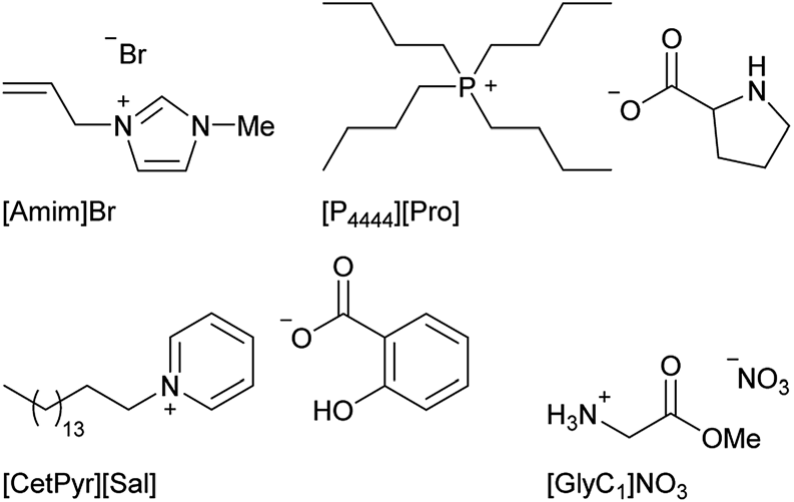
Examples of additional ILs used in cell viability assay.

For biocompatibility measurements, several commonly known ILs, whose cytotoxicity has already been thoroughly explored,^14^ have been studied in comparison to the new carbohydrate based ILs we synthesized. A few examples of these additionally tested ILs are shown in Fig. 8.

**Fig. 9:**
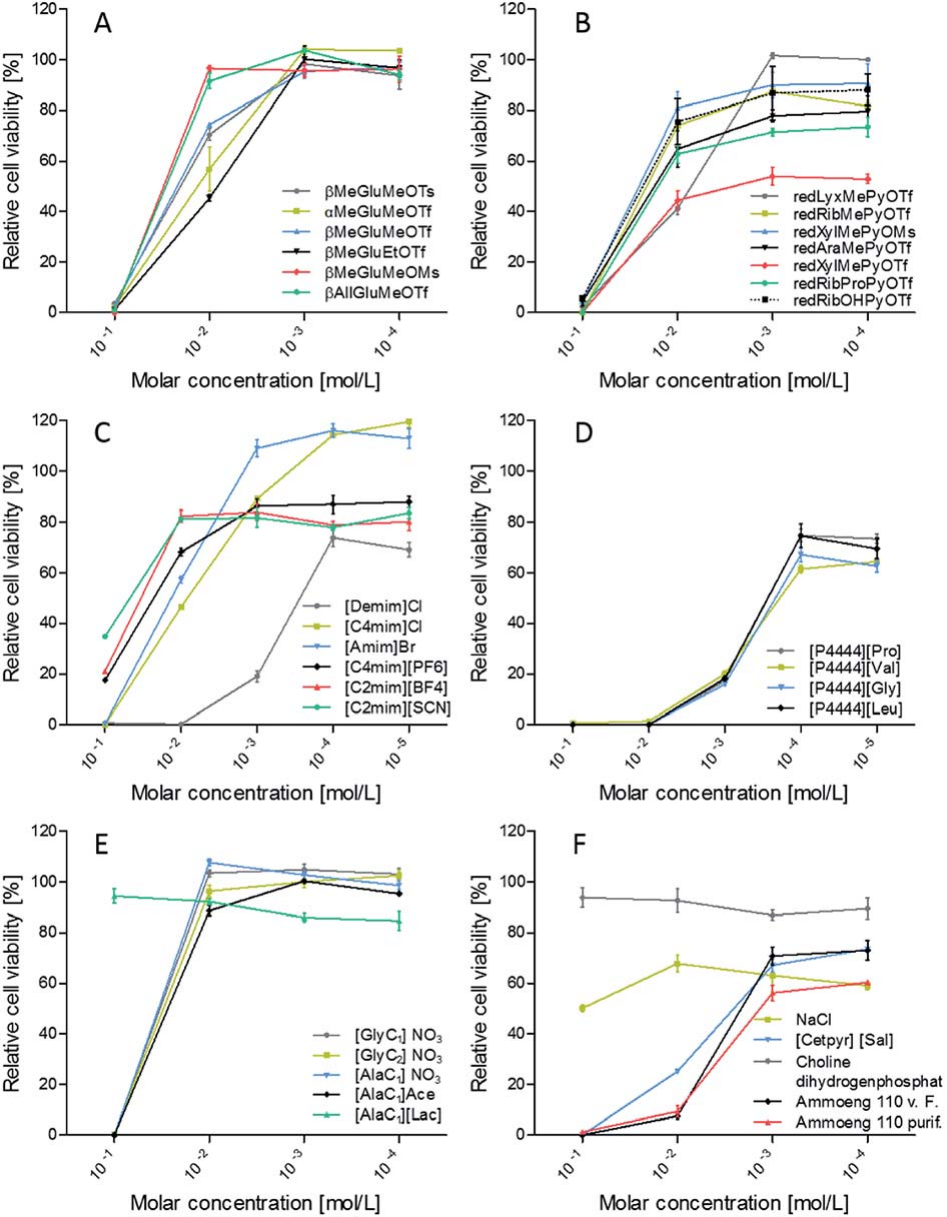
Relative cell viability of L929 mouse fibroblasts after 48 h cultivation in a dilution series of the ionic liquids compared to the pure cell culture medium control (*n* = 4, mean ± SEM). (A) Glucoside based ILs; (B) 1-deoxy-pentose based ILs; (C) imidazolium based ILs; (D) ILs based on tetrabutylphosphonium cation and amino acids as anion; (E) ILs based on amino acids as cation; (F) further natural salts and ILs.

Cell viability assay revealed a strong impact on cell viability at 0.1 mol L^−1^ molar concentration for the predominant share of the evaluated ionic liquids (Fig. 9A–F). According to that only [AlaC_1_][Lac], choline dihydrogenphosphate and sodium chloride, which was tested for comparison, exhibit no zytotoxic effects. Due to the natural occurrence of these substances in cells, this meets our expectations.

Besides that, further discriminations between the ionic liquids presented herein can be made. Comparison between the different glucose derivatives (Fig. 9A) shows almost no viability for any substance at *c* = 0.1 mol L^−1^, whereas major differences can be found at a concentration of 10^−2^ mol L^−1^. A change of the anomeric form from β-glucose to α-glucose results in a decreased viability.

Moreover, results indicate a major impact of the sort of anion on biocompatibility. For βMeGluMePyr^+^ viability rises for following anions in the order: ^−^OTs < ^−^OTf < ^−^OMs. This effect is supported by redXylMePyr^+^ with the counterions ^−^OTf < ^−^OMs (Fig. 9B). Surprisingly, the substitution of the methyl group by an allylic group at the anomeric center diminishes the cytotoxicity of the triflate anion.

For 1-deoxy-pentoses different viabilities could be observed in the following order: lyxose ≤ xylose < arabinose < ribose. Ribose derivatives are naturally found in all nucleotides (DNA, RNA) and hence in all cells, while lyxose can be found in the cell walls of few bacteria and xylose and arabinose in the polysaccharides of plants. Hence, ribose derivatives should be tolerated better by cells with mammalian origin.

Thus, the use of substances with biological origin such as carbohydrates and amino acids is a promising approach for improving the biocompatibility of ionic liquids. To test this, a common tetrabutylphosphonium cation [P_4444_] was countered with four different anions derived from proteinogenic hydrophobic amino acids: proline, valine, glycine and leucine (Fig. 9D). The resulting ionic liquids exhibit cytotoxic effects over the whole concentration range. There are no significant differences between the different anions.

In contrast amino acid derivatives from alanine and glycine as cations exhibit no cytotoxic effects at a concentration of 10^−2^ mol L^−1^ (Fig. 9E). There is also no difference between methoxy- or ethoxy esters in the glycine derivatives. According to the counter ion no significant differences between nitrate and acetate can be found.

Hence, using naturally occurring substances and their derivatives to generate the cation opens up new fields of application, such as biomedical applications. It is worth noting that the IL [AlaC_1_][Lac] shows good viability already at concentrations of 0.1 mol L^−1^, but compared to the other [AlaC_1_] derivatives a slightly worse impact on the biocompatibility at lower concentrations. Further studies have to be performed to shed light on this phenomenon (Tables 1 and 2).

**Table 1:** Relative cell viability of L929 mouse fibroblasts with carbohydrate based ILs

No.	Cation	Anion	EC_50_ [mmol L^−1^]
1i	redRibMePyr	OTf	46.40
2i	redLyxMePyr	OTf	25.19
3i	redXylMePyr	OTf	38.65
4i	redAraMePyr	OTf	40.37
1k	redRibOHPyr	OTf	54.80
1v	redRibPrPyr	OTf	136.62
5f	βMeGluMePyr	OTf	41.73
6f	βAllGluMePyr	OTf	36.40
7f	βPhGluMePyr	OTf	n.m.^a^
8f	αMeGluMePyr	OTf	49.88
5l	βMeGluMePyr	OMs	57.60
5n	βMeGluMePyr	OTs	51.49

a 7f was not measured due to its very poor solubility in water.

**Table 2:** Relative cell viability of L929 mouse fibroblasts with several ILs^15–18^

Cation	Anion	EC_50_ [mmol L^−1^]
P_4444_	Pro	0.575
P_4444_	Val	0.775
P_4444_	Gly	0.875
P_4444_	Leu	0.725
GlyC_1_	NO_3_	35
GlyC_2_	NO_3_	20
AlaC_1_	NO_3_	20
AlaC_1_	Lac	25
Choline	H_2_PO_4_	30
Amim	Br	15
C_2_mim	BF_4_	4
C_2_mim	SCN	8
C_4_mim	Cl	10
C_4_mim	PF_6_	7
C_10_mim (demim)	Cl	0.035
Ammoeng 110®	—	1.07
Ammoeng 110® purif.	—	3.08
Cetpyr	Sal	0.038
Na	Cl	51.85

### Thermal analysis

The analysis of the melting points of our 16 new carbohydrate based pyridinium salts allows the categorization into the class of ionic liquids, which are per definition salts with a melting point under 100 °C.

As such, only βPhGluMePyrOTf 7f, βMeGluEtPyrOTf 5j and βMeGluMePyrOTs 5n do not qualify as ionic liquids, while all other glucoside- and 1-deoxy-pentose products exhibit melting points under 100 °C, with many of them even being room temperature ionic liquids (Tables 3 and 4).

**Table 3:** Melting points and decomposition points of glucoside based products

No.	Cation	Anion	mp [°C]	dp [°C]
5f	βMeGluMePyr	OTf	Liquid at r.t.	225
6f	βAllGluMePyr	OTf	66–70	205
7f	βPhGluMePyr	OTf	164–168	225
8f	αMeGluMePyr	OTf	95–100	242
5j	βMeGluEtPyr	OTf	118–120	215
5l	βMeGluMePyr	OMs	60–63	250
5n	βMeGluMePyr	OTs	135–138	242

**Table 4:** Melting points, decomposition points and glass transition temperature of 1-deoxy-pentose based products

No.	Cation	Anion	mp [°C]	dp [°C]	*T*_g_ [°C]
1i	redRibMePyr	OTf	48–51	345	−18
2i	redLyxMePyr	OTf	Liquid at r.t.	325	−41
3i	redXylMePyr	OTf	32–36	345	−28
4i	redAraMePyr	OTf	Liquid at r.t.	340	−38
1o	redRibEtPyr	OTf	Liquid at r.t.	316	−26
1s	redRibAllPyr	OTf	Liquid at r.t.	301	n.m.
1v	redRibPrPyr	OTf	Liquid at r.t.	235	−27
1k	redRibOHPyr	OTf	Liquid at r.t.	297	−30
1x	redRibIsoPyr	OMs	92–94	296	n.m.

While most of the 1-deoxy-pentose based ILs are liquid at room temperature (Table 4) and such don't allow a direct comparison of the impact of configuration and varying groups on the melting point, such comparisons can be made for the glucoside based products (Table 3).

The type and configuration of the group at the anomeric center has a high influence on the melting point. A trend of βOMe < βOAll ≤ βOPh was found for the glucosides 5–7f. The double bonds of allyl and phenyl groups allow more interactions between the cations than the methyl group, leading to this trend. Furthermore, βMeGluMePyrOTf 5f is liquid at room temperature, while αMeGluMePyrOTf 8f has a melting point close to 100 °C, showing that the configuration of the anomeric center alone has a high impact on the melting point. The change of methyl ether groups in 5f to ethyl ether groups in 5j also heightens the melting point significantly. Lastly the corresponding anion also has a high influence, showing a clear trend of OTf < OMs ≤ OTs.

The decomposition points of the glucoside products vary from 205 to 250 °C, while the 1-deoxy-pentose products have higher values with 297 to 345 °C. In general, these new products have a good thermal stability.

Glass transition temperatures we measured for the 1-deoxy-pentose based IL's to acquire further information of the influences of configuration and varying groups on physical properties. Here we see a trend of lyxose 2i < arabinose 4i < xylose 3i < ribose 1i. The impact of the varying groups in positions 2 and 3 on the glass transition temperature is small, with the methyl ether product 1i having the highest and the free OH group product 1k the lowest *T*_g_ in direct comparison.

## Methods

### Cell viability assay

In order to evaluate the biocompatibility of the carbohydrate-based ILs cytotoxicity was tested with Cell Viability Assay Kit (BioAssay systems, Hayward, CA, USA).

L929 mouse fibroblasts (CCL-1, ATCC) were cultured in DMEM (PAN BIOTECH, Aidenbach, Germany) with 4.5 mg glucose and 10% fetal calf serum (FCS), 1% Penicillin/Streptomycin and 3.7 g L^−1^ NaHCO_3_.

For screening tests 2 × 10^4^ L929 mouse fibroblasts were seeded in a 96-well microtiter plate with 200 μL culture medium per well and incubated under cell culture conditions (37 °C, 5% CO_2_) for 24 hours.

To proof cell viability CellQuanti-Blue Cell Viability Assay Kit (BioAssay systems, Hayward, CA, USA) was implemented. 10% CellQuanti-Blue supplement was added to the wells followed by an incubation of another 2 hours under same conditions. The reductive activity of the cells conducts the metabolic turnover from resazurin to the fluorescent resorufin (absorption 544 nm, emission 590 nm) which was detected with the Fluostar optima (BMG LABTECH, Ortenberg, Germany).

### Thermal analysis

Melting points were determined with a micro heating stage (Mikroheiztisch BOETIUS, Dresden, Germany).

Decomposition points have been measured *via* thermogravimetric analysis using a Labsys 1600 TGA-DSC (SETARAM Instrumentation, Caluire, France).

The differential scanning calorimetry using a Pyris 1 DSC (PerkinElmer, Waltham, USA) allowed the analysis of glass transition temperatures.

## Conclusion

In this work we presented a reproducible strategy to synthesize new carbohydrate based pyridinium salts. Said synthetic pathway was successfully applied on 4 different 1-deoxy-pentoses as well as 4 different glucosides, leading to overall 16 new products with varying configurations and groups.

Thermal analysis shows that 13 of these new carbohydrate based pyridinium salts qualify as ionic liquids per definition, with most of them even being room temperature ionic liquids. Further biocompatibility tests have proven that these ionic liquids are suitable for biomedical applications, as they exhibit a much higher viability than common imidazolium or phosphonium based ILs, which have been tested in comparison. A potential application currently under testing is the usage of these new carbohydrate based ionic liquids as additives in coatings for drug-eluting balloons.

## Conflicts of interest

There are no conflicts to declare.

## Supplementary Material

RA-010-D0RA01370F-s001

RA-010-D0RA01370F-s002
