# Supplementary material for: Synthesis of novel carbohydrate based pyridinium ionic liquids and cytotoxicity of ionic liquids for mammalian cells
Source: RSC Adv. 2020 Apr 7;10(24):14299–304. doi: 10.1039/d0ra01370f (PMC9051878; doi:10.1039/d0ra01370f)
Supplement: RA-010-D0RA01370F-s001 [file RA-010-D0RA01370F-s001.pdf]

Electronic Supplementary Material (ESI) for RSC Advances.
This journal is © The Royal Society of Chemistry 2020

Supporting Information

Synthesis of novel carbohydrate based pyridinium ionic liquids and Cytotoxicity of ionic liquids for mammalian cells

M. Reiß,[a] A. Brietzke,[b] T. Eickner,[b] F. Stein,[a] A. Villinger,[a] C. Vogel,[a] U. Kragl[a] and S. Jopp[a]*

[a]Institute of Chemistry, University of Rostock, Albert-Einstein-Straße 3a, 18059 Rostock, Germany. Email: stefan.jopp@uni-rostock.de

[b]Institute of Biomedical Engineering, University of Rostock, Friedrich-Barnewitz-Straße 4, 18119 Rostock, Germany.

Table of contents:

General information 2
Thiophenyl-tri-*O*-acetyl-pentose glycosides **1-4b** 2
Tri-*O*-acetyl-1-deoxy-pentoses **1-4c** 4
1-Deoxy-pentoses **1-4d** 5
5-*O*-Trityl-1-deoxy-pentoses **1-4e** 7
5-*O*-Trityl-2,3-*O*-methyl-1-deoxy-pentoses **1-4f** + ethyl and allyl ethers **1l** and **1p** 8
2,3-*O*-Methyl-1-deoxy-pentoses **1-4g** + ethyl and allyl ethers **1m** and **1q** 11
Reduction of allyl ether **1q** to propyl ether **1t** 13
Synthesis of 1-deoxy-pentose based pyridinium triflate salts **1-4i**, **1o**, **1s** and **1v** 13
2,3-*O*-Isopropylidene-1-deoxy-D-ribofuranoside products **1j**, **1k** and **1x** 18
6-*O*-Trityl-glucopyranosides **5-8b** 21
6-*O*-Trityl-2,3,4-*O*-methyl-glucopyranosides **5-8c** and ethyl ether **5g** 22
2,3,4-*O*-Methyl-glucopyranosides **5-8d** and ethyl ether **5h** 25
Synthesis of glucopyranoside based pyridinium triflate salts **5-8f** and **5j** 27
Synthesis of glucopyranoside based pyridinium mesylate salt **5l** 30
Synthesis of glucopyranoside based pyridinium tosylate salt **5n** 31
NMR Spectra of all final pentose based ionic products 34
NMR Spectra of all final glucoside based ionic products 47
NMR spectra of key pentose intermediates 57
NMR Spectra of key glucoside intermediates 62

General information

All reagents and solvents were purchased from commercial sources and used as received without further purification, if not stated otherwise. The NMR spectra were recorded on a Bruker AVANCE 300 III, 250 II or 500. The IR spectra were measured as ATR experiments with a Nicolet 6700 FT-IR spectrometer and a Nicolet 550 FT-IR spectrometer. MS and HRMS were measured by an Agilent 6890 N/5973 GC-MS and an Agilent 1200/6210 Time-of-Flight LC-MS.

General procedure for synthesis of thiophenyl-tri-*O*-acetyl-pentose glycosides 1-4b.

Peracetylated D-ribose **1a**, D-lyxose **2a**, D-xylose **3a** or L-arabinose **4a** (1.20 g, 3.77 mmol) was dissolved in dichloromethane (24 mL) and cooled to 0°C. grinded molecular sieves (1 spatula tip) and thiophenol (0.46 mL, 4.52 mmol) were added and the reaction was stirred at 0°C for 15 min. $BF_3 \cdot OEt_2$ (2.40 mL, 18.93 mmol) was added slowly and the reaction was stirred further 1 h at 0°C followed by 1 h at room temperature. The reaction was then neutralized with NEt_3 (6 mL) and dichloromethane (100 mL) was added. The reaction was washed with brine (2x 30 mL) and cold water (2x 30 mL), dried and evaporated. Column chromatography (PE/EA 2:1 to 1:1) led to products **1-4b**.

Thiophenyl-2,3,5-tri-*O*-acetyl-α-D-ribofuranoside 1b

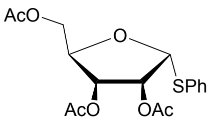

Yield: 1.11 g (80 %); R_f = 0.49 (PE/EA 1:1); $[\alpha]_D^{22}$ = -51.4° (c = 1.2, $CHCl_3$); ^1H NMR (300 MHz, $CDCl_3$): δ = 7.56–7.50 (m, 2H, CH_{Ar}), 7.36–7.31 (m, 3H, CH_{Ar}), 5.34–5.30 (d, 1H, $^3J_{H-1,H-2}$ = 4.91 Hz, H-1), 5.27–5.21 (m, 2H, H-2, H-3), 4.31–4.23 (m, 2H, H-4, H-5a), 4.10 (dd, 1H, H-5b), 2.10 (s, 3H, CH_3), 2.08 (s, 3H, CH_3), 2.06 (s, 3H, CH_3); ^{13}C NMR (75 MHz, $CDCl_3$): δ = 170.5, 169.6, 169.4 (3x C=O), 133.4, 129.2 (CH_{Ar}), 131.7 (C_{Ar}), 128.4 (CH_{Ar}), 87.9 (C-1), 80.1 (C-4), 73.9, 71.4 (C-2, C-3), 63.4 (C-5), 20.8, 20.5, 20.5 (3x CH_3); HRMS (ESI), m/z calc. for $C_{17}H_{20}NaO_7S$ $[M+Na]^+$: 391.082, found: 391.083; Elemental Analysis for $C_{17}H_{20}O_7S$: C: 55.42, H: 5.47, found: C: 55.33, H: 5.55.

Thiophenyl-2,3,5-tri-*O*-acetyl-α-D-lyxofuranoside 2b

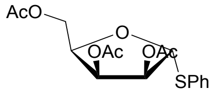

Yield: 0.69 g (50 %); R_f = 0.46 (PE/EA 2:1); $[\alpha]_D^{21}$ = -72.6° (c = 1.0, $CHCl_3$); ^1H NMR (300 MHz, $CDCl_3$): δ = 7.57–7.51 (m, 2H, CH_{Ar}), 7.37–7.32 (m, 3H, CH_{Ar}), 5.50 (d, 1H, H-3), 5.46 (d, 1H, $^3J_{H-1,H-2}$ = 5.99 Hz, H-1), 5.32 (dd, 1H, H-2), 4.51–4.43 (m, 1H, H-4), 4.27 (s, 1H, H-5a), 4.24 (d, 1H, H-5b), 2.11 (s, 3H, CH_3), 2.08 (s, 6H, CH_3); ^{13}C NMR (75 MHz, $CDCl_3$): δ = 170.5, 169.6, 169.3 (3x C=O), 132.8, 129.1, 128.2 (CH_{Ar}), 87.7 (C-1), 77.2 (C_{Ar}), 76.4 (C-4), 75.0 (C-2), 70.9 (C-3), 61.7 (C-5), 20.8, 20.5, 20.4 (3x CH_3); HRMS (ESI), m/z calc. for $C_{17}H_{20}NaO_7S$ $[M+Na]^+$: 391.082, found: 391.082; Elemental Analysis for $C_{17}H_{20}O_7S$: C: 55.42, H: 5.47, S: 8.70, found: C: 55.33, H: 5.64, S: 8.60.

Thiophenyl-2,3,5-tri-*O*-acetyl-α-D-xylofuranoside 3b

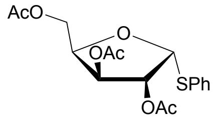

Yield: 1.04 g (74 %); R_f = 0.60 (PE/EA 1:1); $[\alpha]_D^{21}$ = -73.8° (c = 1.0, $CHCl_3$); ^1H NMR (500 MHz, $CDCl_3$): δ = 7.49–7.46 (m, 2H, CH_{Ar}), 7.40–7.29 (m, 3H, CH_{Ar}), 5.44 (d, 1H, $^3J_{H-1,H-2}$ = 3.15 Hz, H-1), 5.30 (dd, 1H, $^3J_{H-2,H-3}$ = 2.21 Hz, H-3), 5.14 (dd, 1H, H-2), 4.47 (dt, 1H, $^3J_{H-4,H-5a}$ = 4.73 Hz, H-4), 4.25 (dd, 1H, H-5a), 4.13 (dd, 1H, H-5b), 2.08 (s, 3H, CH_3), 2.03 (s, 3H, CH_3), 2.02 (s, 3H, CH_3); ^{13}C NMR (125 MHz, $CDCl_3$): δ = 169.9, 169.3, 169.1 (3x C=O), 133.3 (C_{Ar}), 131.1, 129.1, 127.5 (CH_{Ar}), 88.2 (C-1), 79.7 (C-2), 78.1 (C-4), 74.9 (C-3), 61.7 (C-5), 20.5, 20.5, 20.3 (3x CH_3); HRMS (ESI), m/z calc. for $C_{17}H_{20}NaO_7S$ $[M+Na]^+$: 391.082, found: 391.082; Elemental Analysis for $C_{17}H_{20}O_7S$: C: 55.42, H: 5.47, S: 8.70, found: C: 55.37, H: 5.34, S: 8.80.

Thiophenyl-2,3,5-tri-*O*-acetyl-α-L-arabinofuranoside 4b

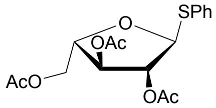

Yield: 1.15 g (83 %); R_f = 0.47 (PE/EA 1:1); $[\alpha]_D^{22}$ = -159.5° (c = 1.0, $CHCl_3$); ^1H NMR (300 MHz, $CDCl_3$): δ = 7.53–7.49 (m, 2H, CH_{Ar}), 7.36–7.28 (m, 3H, CH_{Ar}), 5.55 (d, 1H, $^3J_{H-1,H-2}$ = 2.08 Hz, H-1), 5.29 (t, 1H, $^3J_{H-2,H-3}$ = 2.27 Hz, H-2), 5.09 (dd, 1H, H-3), 4.54–4.45 (m, 1H, H-4), 4.41 (dd, 1H, $^3J_{H-4,H-5a}$ = 11.90 Hz, H-5a); 4.29 (dd, 1H, H-5b); 2.13 (s, 3H, CH_3), 2.11 (s, 3H, CH_3), 2.10 (s, 3H, CH_3); ^{13}C NMR (75 MHz, $CDCl_3$): δ = 170.5, 170.0, 169.5 (3x

C=O), 133.4 (C_{Ar}), 132.0, 129.0, 127.8 (CH_{Ar}), 90.9 (C-1), 81.7 (C-2); 80.0 (C-4), 77.1 (C-3), 62.8 (C-5); 20.7, 20.7, 20.7 (3x CH_3); HRMS (ESI), m/z calc. for $C_{17}H_{20}NaO_7S$ $[M+Na]^+$: 391.082, found: 391.083; Elemental Analysis for $C_{17}H_{20}O_7S$: C: 55.42, H: 5.47, S: 8.70, found: C: 55.42, H: 5.49, S: 8.80.

General procedure for synthesis of tri-*O*-acetyl-1-deoxy-pentoses 1-4c.

1-4b (650 mg, 1.76 mmol) was dissolved in toluene (20 mL) and tributyltin hydride (886 µL, 3.35 mmol) and AIBN (68.2 mg) were added. The reaction was heated under reflux for 2.5 h and evaporated afterwards. The crude reaction mixture was dissolved in diethyl ether (100 mL), washed with a 10 % KF solution (2x 30 mL) and cold water (2x 30 mL), dried and evaporated again. Column chromatography (Tol/EA 10:1) led to products **1-4c**.

1-Deoxy-2,3,5-tri-*O*-acetyl-D-ribofuranoside 1c

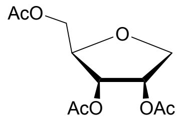

Yield: 390 mg (85 %); $R_f = 0.42$ (PE/EA 1:1); $[\alpha]_D^{22} = 70.7°$ (c = 1.5, $CHCl_3$); ^1H NMR (500 MHz, $CDCl_3$): δ = 5.37 (dt, 1H, $^3J_{H-2,H-3} = 5.36$ Hz, H-2), 5.14 (dt, 1H, H-3), 4.34 (dt, 1H, H-5a), 4.20–4.08 (m, 2H, H-4, H-5b), 3.88 (dd, 1H, H-1b), 2.10 (s, 3H, CH_3), 2.10 (s, 3H, CH_3), 2.09 (s, 3H, CH_3); ^{13}C NMR (125 MHz, $CDCl_3$): δ = 170.6, 169.9, 169.8 (3x C=O), 78.0 (C-4), 71.8 (C-2), 71.2 (C-3), 70.8 (C-1), 63.5 (C-5), 20.8, 20.6, 20.5 (3x CH_3); HRMS (ESI), m/z calc. for $C_{11}H_{16}NaO_7$ $[M+Na]^+$: 283.079, found: 283.079; Elemental Analysis for $C_{11}H_{16}O_7$: C: 50.77, H: 6.20, found: C: 50.57, H: 6.29.

1-Deoxy-2,3,5-tri-*O*-acetyl-D-lyxofuranoside 2c

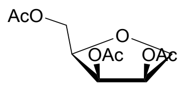

Yield: 229 mg (50 %); $R_f = 0.41$ (PE/EA 1:1); $[\alpha]_D^{22} = 11.9°$ (c = 1.0, $CHCl_3$); ^1H NMR (500 MHz, $CDCl_3$): δ = 5.49 (t, 1H, $^3J_{H-2,H-3} = 5.10$ Hz, H-3), 5.40 (dd, 1H, $^3J_{H-1a,H-2} = 6.04$ Hz, H-2), 4.32–4.19 (m, 3H, H-4, H-5a, H-5b), 4.09 (dd, 1H, $^3J_{H-1a,H-1b} = 10.01$ Hz, H-1a), 3.92 (dd, 1H, H-1b), 2.11 (s, 3H, CH_3), 2.08 (s, 3H, CH_3), 2.07 (s, 3H, CH_3); ^{13}C NMR (125 MHz, $CDCl_3$): δ = 170.7, 169.9, 169.7 (3x C=O), 76.4 (C-4), 71.6, 71.3 (C-2, C-3), 69.6 (C-1), 62.7 (C-5), 20.8, 20.6, 20.4 (3x CH_3); HRMS (ESI), m/z calc. for $C_{11}H_{16}NaO_7$ $[M+Na]^+$: 283.079, found: 283.079; Elemental Analysis for $C_{11}H_{16}O_7$: C: 50.77, H: 6.20, found: C: 50.73, H: 6.26.

1-Deoxy-2,3,5-tri-*O*-acetyl-D-xylofuranoside 3c

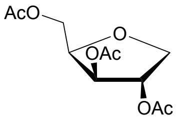

Yield: 320 mg (70 %); R_f = 0.50 (PE/EA 1:1); $[\alpha]_D^{21}$ = 57.5° (c = 1.0, $CHCl_3$); ^1H NMR (300 MHz, $CDCl_3$): δ = 5.36 (dd, 1H, $^3J_{H\text{-}2,H\text{-}3}$ = 2.46 Hz, H-3), 5.14 (ddd, 1H, H-2), 4.34–4.23 (m, 3H, H-4, H-1a, H-1b), 4.16 (dd, 1H, H-5a), 3.77 (dd, 1H, H-5b), 2.10 (s, 3H, CH_3), 2.09 (s, 3H, CH_3), 2.08 (s, 3H, CH_3); ^{13}C NMR (75 MHz, $CDCl_3$): δ = 170.6, 169.8, 169.5 (3x C=O), 77.3 (C-2), 77.3 (C-4), 76.0 (C-3), 71.8 (C-1), 61.9 (C-5), 20.8, 20.8, 20.6 (3x CH_3); HRMS (ESI), m/z calc. for $C_{11}H_{16}NaO_7$ $[M+Na]^+$: 283.079, found: 283.079; Elemental Analysis for $C_{11}H_{16}O_7$: C: 50.77, H: 6.20, found: C: 50.54, H: 6.34.

1-Deoxy-2,3,5-tri-*O*-acetyl-L-arabinofuranoside 4c

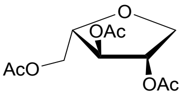

Yield: 298 mg (65 %); R_f = 0.67 (PE/EA 1:1); $[\alpha]_D^{23}$ = 18.7° (c = 1.5, $CHCl_3$); ^1H NMR (500 MHz, $CDCl_3$): δ = 5.20–5.17, 5.07–5.04 (2x m, 2H, H-2, H-3), 4.36 (dd, 1H, H-5a), 4.21 (dd, 1H, H-5b), 4.08 (dd, 1H, H-1a), 4.05–3.98 (m, 2H, H-1b, H-4), 2.11 (s, 3H, CH_3), 2.10 (s, 3H, CH_3), 2.10 (s, 3H, CH_3); ^{13}C NMR (125 MHz, $CDCl_3$): δ = 170.6, 170.0, 169.8 (3x C=O), 81.7 (C-4), 78.3, 77.7 (C-2, C-3), 72.1 (C-1), 63.5 (C-5), 20.8, 20.8, 20.7 (3x CH_3); HRMS (ESI), m/z calc. for $C_{11}H_{16}NaO_7$ $[M+Na]^+$: 283.079, found: 283.079; Elemental Analysis for $C_{11}H_{16}O_7$: C: 50.77, H: 6.20, found: C: 50.56, H: 6.49.

General procedure for synthesis of 1-deoxy-pentoses 1-4d.

1-4c (520 mg, 2.00 mmol) was dissolved in dry methanol (100 mL) und cooled to 0°C. Sodium was added in small portions until pH was 12. The reaction was stirred at room temperature for 4 hours. The reaction was neutralized with Amberlite H^+ ion exchange resin, filtrated and evaporated. Column chromatography ($CHCl_3$/MeOH 10:1 to 5:1) led to products **1-4d**.

1-Deoxy-D-ribofuranoside 1d

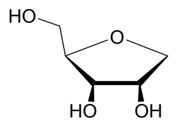

Yield: 241 mg (90 %); m.p. = 95–101°C; R_f = 0.29 ($CHCl_3$/MeOH 4:1); $[\alpha]_D^{23}$ = 60.6° (c = 1.0, MeOH); ^{1}H NMR (300 MHz, MeOD): δ = 4.14 (m, 1H), 4.05–3.93 (m, 2H, H-1a, H-1b), 3.80–3.67 (m, 3H), 3.57 (dd, 1H, H-5b); ^{13}C NMR (63 MHz, MeOD): δ = 73.9 (C-1), 84.3, 73.3, 72.6 (C-2, C-3, C-4), 63.3 (C-5); HRMS (ESI), m/z calc. for $C_5H_{10}NaO_4$ $[M+Na]^+$: 157.047, found: 157.047; Elemental Analysis for $C_5H_{10}O_4$: C: 44.77, H: 7.51, found: C: 44.69, H: 7.57.

1-Deoxy-D-lyxofuranoside 2d

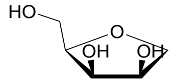

Product **2d** was not free from impurities after column chromatography. The crude product was directly used for the next reaction, see **2e**.

1-Deoxy-D-xylofuranoside 3d

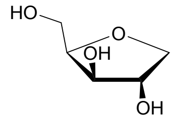

Yield: 201 mg (73 %); R_f = 0.35 ($CHCl_3$/MeOH 4:1); $[\alpha]_D^{23}$ = -15.3° (c = 1.0, MeOH); ^{1}H NMR (300 MHz, MeOD): δ = 4.14 (m, 1H), 4.15–3.99 (m, 4H, H-1a, H-2, H-3, H-4), 3.80 (dd, 1H, H-5a), 3.72 (dd, 1H, H-5b), 3.65 (m, 1H, H-1b); ^{13}C NMR (75 MHz, MeOD): δ = 82.4, 78.8, 78.3 (C-2, C-3, C-4), 74.4 (C-1), 61.7 (C-5); HRMS (ESI), m/z calc. for $C_5H_{10}NaO_4$ $[M+Na]^+$: 157.047, found: 157.047; Elemental Analysis for $C_5H_{10}O_4$: C: 44.77, H: 7.51, found: C: 44.56, H: 7.70.

1-Deoxy-L-arabinofuranoside 4d

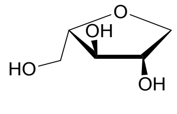

Yield: 233 mg (87 %); R_f = 0.25 ($CHCl_3$/MeOH 4:1); $[\alpha]_D^{18}$ = -16.5° (c = 1.0, MeOH); ^{1}H NMR (500 MHz, MeOD): δ = 4.08–4.03 (m, 1H, H-2), 3.99–3.92 (m, 2H, H-1a, H-3), 3.82–3.74 (m, 2H, H-1b, H-4), 3.69–3.64 (m, 2H, H-5a, H-5b); ^{13}C NMR (75 MHz, MeOD): δ = 87.9 (C-4), 80.0 (C-3), 78.9 (C-2), 74.8 (C-1), 63.7 (C-5); HRMS (ESI), m/z calc. for $C_5H_{10}NaO_4$ $[M+Na]^+$:

157.047, found: 157.047; Elemental Analysis for $C_5H_{10}O_4$: C: 44.77, H: 7.51, found: C: 44.49, H: 7.69.

General procedure for synthesis of 5-*O*-trityl-1-deoxy-pentoses 1-4e.

1-4d (1.34 g, 10.0 mmol), trityl chloride (4.18 g, 15.0 mmol), DMAP (one spatula tip) and NEt_3 (6.7 mL) were stirred at room temperature overnight in dichloromethane (25 mL). The reaction mixture was evaporated. Column chromatography (PE/EA 4:1 to EA) led to products **1-4e**.

5-*O*-Trityl-1-deoxy-D-ribofuranoside 1e

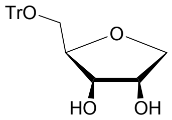

Yield: 3.01 g (80 %); m.p. = 143°C; R_f = 0.69 (EA); $[\alpha]_D^{22}$ = 42.5° (c = 1.0, MeOH); ^{1}H NMR (300 MHz, DMSO-d_6): δ = 7.42–7.23 (m, 15H, CH_{Ar}), 4.78 (d, 1H, OH), 4.74 (d, 1H, OH), 4.03–3.99 (m, 1H, $^3J_{H\text{-}1a,H\text{-}2}$ = 4.73 Hz, H-2), 3.96 (dd, 1H, H-1a), 3.81–3.74 (m, 2H, H-3,H-4), 3.60 (dd, 1H, $^3J_{H\text{-}1b,H\text{-}2}$ = 9.46 Hz, H-1b), 3.12 (dd, 1H, $^3J_{H\text{-}5a,H\text{-}5b}$ = 9.77 Hz, H-5a), 2.97 (dd, 1H, H-5b); ^{13}C NMR (75 MHz, DMSO-d_6): δ = 143.8 (C_{Ar}), 128.3, 127.8, 126.9 (CH_{Ar}), 85.7 (*C*Ph_3), 80.8 (C-4), 72.5 (C-1), 72.2, 70.3 (C-2, C-3), 64.6 (C-5); HRMS (ESI), m/z calc. for $C_{24}H_{24}NaO_4$ [M+Na]$^+$: 399.157, found: 399.157; Elemental Analysis for $C_{24}H_{24}O_4$: C: 76.57, H: 6.43, found: C: 76.46, H: 6.48.

5-*O*-Trityl-1-deoxy-D-lyxofuranoside 2e

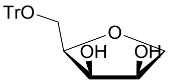

Yield: 2.07 g (55 %*); R_f = 0.26 (PE/EA 1:1); $[\alpha]_D^{23}$ = 25.9° (c = 1.0, $CHCl_3$); ^{1}H NMR (500 MHz, $CDCl_3$): δ = 7.44–7.23 (m, 15H, CH_{Ar}), 4.30 (t, 1H, $^3J_{H\text{-}3,H\text{-}4}$ = 5.36 Hz, H-3), 4.25 (t, 1H, $^3J_{H\text{-}1b,H\text{-}2}$ = 4.41 Hz, H-2),4.08–4.04 (m, 1H, H-4), 3.94 (dd, 1H, H-1a), 3.90 (dd, 1H, H-1b), 3.57 (dd, 1H, H-5a), 3.32 (dd, 1H, H-5b); ^{13}C NMR (125 MHz, $CDCl_3$): δ = 143.2 (C_{Ar}), 128.5, 128.1, 127.3 (CH_{Ar}), 87.9 (*C*Ph_3), 78.6 (C-4), 72.6 (C-3), 72.5 (C-1), 71.9 (C-2), 62.9 (C-5); HRMS (ESI), m/z calc. for $C_{24}H_{24}NaO_4$ [M+Na]$^+$: 399.157, found: 399.157; Elemental Analysis for $C_{24}H_{24}O_4$: C: 76.57, H: 6.43, found: C: 76.48, H: 6.49.

*yield over two steps, impure product **2d** was used for this reaction.

5-*O*-Trityl -1-deoxy-D-xylofuranoside 3e

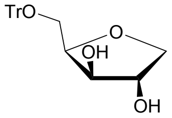

Yield: 3.31 g (88 %); R_f = 0.49 (EA); $[\alpha]_D^{22}$ = 16.3° (c = 1.0, $CHCl_3$); ^{1}H NMR (500 MHz, DMSO-d_6): δ = 7.44–7.23 (m, 15H, CH_{Ar}), 5.04 (d, 1H, OH), 4.87 (d, 1H, OH), 4.07 (dd, 1H, H-4), 3.94 (t, 1H, H-2), 3.90 (dd, 1H, H-1a), 3.84 (t, 1H, H-3), 3.49 (d, 1H, H-1b), 3.14–3.10 (m, 2H, H-5a, H-5b); ^{13}C NMR (125 MHz, DMSO-d_6): δ = 143.9 (C_{Ar}), 128.3, 127.8, 126.9 (CH_{Ar}), 85.8 (*C*Ph_3), 79.5 (C-4), 76.6, 76.3 (C-2, C-3), 72.9 (C-1), 62.8 (C-5); HRMS (ESI), m/z calc. for $C_{24}H_{24}NaO_4$ [M+Na]$^+$: 399.157, found: 399.157; Elemental Analysis for $C_{24}H_{24}O_4$: C: 76.57, H: 6.43, found: C: 76.48, H: 6.47.

5-*O*-Trityl-1-deoxy-L-arabinofuranoside 4e

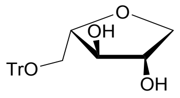

Yield: 2.82 g (75 %); R_f = 0.38 (EA); $[\alpha]_D^{22}$ = -46.6° (c = 1.0, $CHCl_3$); ^{1}H NMR (300 MHz, DMSO-d_6): δ = 7.45–7.20 (m, 15H, CH_{Ar}), 5.15 (d, 1H, OH), 4.92 (d, 1H, OH), 3.96–3.90 (m, 1H, H-3), 3.85 (dd, 1H, H-5a), 3.81–3.72 (m, 2H, H-2, H-4), 3.61 (dd, 1H, H-5b), 3.12–3.01 (m, 2H, H-1a, H-1b); ^{13}C NMR (75 MHz, DMSO-d_6): δ = 143.9 (C_{Ar}), 128.3, 127.8, 126.9 (CH_{Ar}), 85.8 (*C*Ph_3), 84.4, 78.6 (C-2, C-4), 76.9 (C-3), 73.0 (C-1), 64.6 (C-5); HRMS (ESI), m/z calc. for $C_{24}H_{24}NaO_4$ [M+Na]$^+$: 399.157, found: 399.157; Elemental Analysis for $C_{24}H_{24}O_4$: C: 76.57, H: 6.43, found: C: 76.32, H: 6.43.

General procedure for synthesis of 5-*O*-trityl-2,3-*O*-methyl-1-deoxy-pentoses 1-4f as well as ethyl and allyl ethers 1l and 1p

1-4e (2.07 g, 5.5 mmol) was dissolved in dry DMF (33 mL) and cooled to 0°C. NaH (60 % dispersion in mineral oil, 1.3 eq per OH group) was added in small portions. The reaction was stirred at 0°C for 30 min, then methyl iodide (2.0 eq per OH group) was added and the reaction was stirred over night at room temperature. The solvent was evaporated, dichlormethane (100 mL) was added and the mixture was washed (3x 30 mL). Column chromatography (PE/EA 3:1) led to products **1-4f**.

For ethylation, **1e** (2.07 g, 5.5 mmol) and ethyl bromide (2.0 eq per OH group) was used, leading to product **1l**.

For allylation, **1e** (1.69 g, 4.5 mmol) and allyl bromide (1.56 mL, 18.0 mmol) was used, leading to product **1p**.

5-*O*-Trityl-2,3-*O*-methyl-1-deoxy-D-ribofuranoside 1f

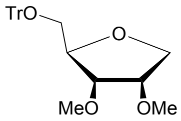

Yield: 2.11 g (95 %); R_f = 0.55 (PE/EA 1:1); $[\alpha]_D^{22}$ = 35.4° (c = 1.5, $CHCl_3$); ^1H NMR (500 MHz, $CDCl_3$): δ = 7.49–7.45, 7.33–7.29, 7.27–7.22 (m, 15H, CH_{Ar}), 4.11–4.04 (m, 2H, H-1a, H-4), 3.97–3.92 (m, 2H, H-1b, H-2), 3.82 (t, 1H, H-3), 3.45 (s, 3H, CH_3), 3.38 (s, 3H, CH_3), 3.34 (dd, 1H, H-5a), 3.14 (dd, 1H, H-5b); ^{13}C NMR (75 MHz, $CDCl_3$): δ = 143.9 (C_{Ar}), 128.7, 127.8, 127.0 (CH_{Ar}), 86.6 (CPh_3), 81.0 (C-3), 80.5 (C-4), 79.3 (C-2), 69.6 (C-1), 64.3 (C-5), 58.0, 57.7 (2x CH_3); HRMS (ESI), m/z calc. for $C_{26}H_{28}NaO_4$ $[M+Na]^+$: 427.188, found: 427.189; Elemental Analysis for $C_{26}H_{28}O_4$: C: 77.20, H: 6.98, found: C: 77.31, H: 6.85.

5-*O*-Trityl-2,3-*O*-methyl-1-deoxy-D-lyxofuranoside 2f

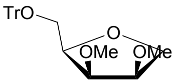

Yield: 2.11 g (95 %); m.p. = 96–99°C; R_f = 0.74 (PE/EA 1:1); $[\alpha]_D^{23}$ = -6.8° (c = 1.0, $CHCl_3$); ^1H NMR (300 MHz, DMSO-*d6*): δ = 7.46–7.21 (m, 15H, CH_{Ar}), 4.12 (dt, 1H, $^3J_{H-4,H-5b}$ = 5.10 Hz, H-4), 4.05–3.88 (m, 2H, H-2, H-3), 3.80 (dd, 1H, H-1a), 3.54 (dd, 1H, H-1b), 3.25 (s, 3H, CH_3), 3.24 (s, 3H, CH_3), 3.14 (dd, 1H, H-5a), 3.02 (dd, 1H, H-5b); ^{13}C NMR (75 MHz, DMSO-*d6*): δ = 143.8 (C_{Ar}), 128.2, 127.8, 126.9 (CH_{Ar}), 85.9 (CPh_3), 80.0 (C-2), 79.1 (C-3), 78.4 (C-4), 67.9 (C-1), 62.4 (C-5), 58.7, 57.3 (2x CH_3); HRMS (ESI), m/z calc. for $C_{26}H_{28}NaO_4$ $[M+Na]^+$: 427.188, found: 427.188; Elemental Analysis for $C_{26}H_{28}O_4$: C: 77.20, H: 6.98, found: C: 77.14, H: 7.17.

5-*O*-Trityl-2,3-*O*-methyl-1-deoxy-D-xylofuranoside 3f

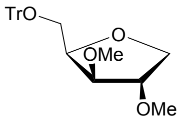

Yield: 2.11 g (95 %); R_f = 0.80 (PE/EA 1:1); $[\alpha]_D^{22}$ = -40.1° (c = 1.0, $CHCl_3$); ^1H NMR (500 MHz, DMSO-*d6*): δ = 7.41–7.23 (m, 15H, CH_{Ar}), 4.05–3.89 (m, 1H, H-4), 3.90–3.85 (m, 2H, H-1a, H-2), 3.77 (d, 1H, H-3), 3.62–3.57 (m, 1H, H-1b), 3.30 (s, 3H, CH_3), 3.22 (s, 3H, CH_3), 3.13–3.05 (m, 2H, H-5a, H-5b); ^{13}C NMR (125 MHz, DMSO-*d6*): δ = 143.7 (C_{Ar}), 128.2, 127.8, 127.0 (CH_{Ar}), 85.9 (CPh_3), 83.0 (C-3), 82.6 (C-2), 79.2 (C-4), 70.4 (C-1), 61.5 (C-5), 56.9, 56.3 (2x CH_3); HRMS (ESI), m/z calc. for $C_{26}H_{28}NaO_4$ $[M+Na]^+$: 427.188, found: 427.189; Elemental Analysis for $C_{26}H_{28}O_4$: C: 77.20, H: 6.98, found: C: 76.94, H: 7.11.

5-*O*-Trityl-2,3-*O*-methyl-1-deoxy-L-arabinofuranoside 4f

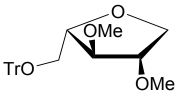

Yield: 1.94 g (87 %); R_f = 0.59 (PE/EA 1:1); $[\alpha]_D^{22}$ = -3.2° (c = 1.0, $CHCl_3$); ^{1}H NMR (500 MHz, $CDCl_3$): δ = 7.51–7.44, 7.33–7.19 (m, 15H, CH_{Ar}), 4.00-3.93 (m, 1H, $^3J_{H-4,H-5}$ = 5.67 Hz, H-4), 3.92–3.85 (m, 2H, $^3J_{H-1H-2}$ = 4.15 Hz, H-1a, H-1b), 3.81–3.76 (m, 1H, H-2), 3.74–3.70 (m, 1H, H-3), 3.37 (s, 3H, CH_3), 3.32 (dd, 1H, H-5a), 3.27 (s, 3H, CH_3), 3.19 (dd, 1H, H-5b); ^{13}C NMR (125 MHz, $CDCl_3$): δ = 143.9 (C_{Ar}), 128.7, 127.8, 126.9 (CH_{Ar}), 86.6 (*C*Ph_3), 86.2 (C-3), 85.2 (C-2), 82.7 (C-4), 71.2 (C-1), 63.9 (C-5), 57.4, 56.7 (2x CH_3); HRMS (ESI), m/z calc. for $C_{26}H_{28}NaO_4$ $[M+Na]^+$: 427.188, found: 427.188; Elemental Analysis for $C_{26}H_{28}O_4$: C: 77.20, H: 6.98, found: C: 76.91, H: 6.99.

5-*O*-Trityl-2,3-*O*-ethyl-1-deoxy-D-ribofuranoside 1l

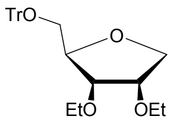

Yield: 2.26 g (95 %); R_f = 0.60 (PE/EA 4:1); $[\alpha]_D^{25}$ = 36.4° (c = 1.0, $CHCl_3$); ^{1}H NMR (300 MHz, DMSO-d_6): δ = 7.44–7.21 (m, 15H, CH_{Ar}), 4.05–3.97 (m, 1H, H-2), 3.92 (dd, 1H, H-1a), 3.88–3.77 (m, 2H, H-3, H-4), 3.73 (dd, 1H, H-1b), 3.48–3.36 (m, 4H, 2x CH_2), 3.12 (dd, 1H, H-5a), 2.94 (dd, 1H, H-5b), 1.12–1.04 (m, 6H, 2x CH_3); ^{13}C NMR (75 MHz, DMSO-d_6): δ = 143.7 (C_{Ar}), 128.2, 127.9, 127.0 (CH_{Ar}), 85.8 (*C*Ph_3), 79.3, 79.0 (C-3, C-4), 76.7 (C-2), 70.1 (C-1), 64.7, 64.5 (2x CH_2), 63.8 (C-5), 15.4, 15.2 (2x CH_3); HRMS (ESI), m/z calc. for $C_{28}H_{32}NaO_4$ $[M+Na]^+$: 455.219, found: 455.220; Elemental Analysis for $C_{28}H_{32}O_4$: C: 77.75, H: 7.46, found: C: 78.03, H: 7.67.

5-*O*-Trityl-2,3-*O*-allyl-1-deoxy-D-ribofuranoside 1p

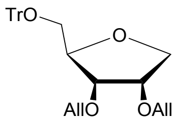

Yield: 1.95 g (95 %); R_f = 0.65 (PE/EA 4:1); $[\alpha]_D^{25}$ = 30.3° (c = 1.0, $CHCl_3$); ^{1}H NMR (300 MHz, $CDCl_3$): δ = 7.42–7.21 (m, 15H, CH_{Ar}), 5.98–5.72 (m, 2H, 2x C*H*=CH_2), 5.33–5.05 (m, 4H, 2x CH=C*H*$_2$), 4.10–3.86 (m, 8H, 2x OCH_2, H-1a, H-2, H-3, H-4), 3.77 (dd, 1H, H-1b), 3.15 (dd, 1H, H-5a), 2.97 (dd, 1H, H-5b); ^{13}C NMR (75 MHz, $CDCl_3$): δ = 143.7 (C_{Ar}), 135.3, 134.9 (*C*H=CH_2), 128.2, 127.8, 127.0 (CH_{Ar}), 116.5, 116.4 (CH=*C*H_2), 85.8 (*C*Ph_3), 79.5,

78.5, 76.4 (C-2, C-3, C-4), 70.1 (C-1), 70.1, 70.0 (OCH_2), 63.8 (C-5); HRMS (ESI), m/z calc. for $C_{30}H_{32}NaO_4$ $[M+Na]^+$: 479.219, found: 479.220; Elemental Analysis for $C_{30}H_{32}O_4$: C: 78.92, H: 7.06, found: C: 79.24, H: 7.17.

General procedure for synthesis of 2,3-*O*-methyl-1-deoxy-pentoses 1-4g as well as ethyl and allyl ethers 1m and 1q

1-4f, **1l** or **1p** (4.0 mmol) was heated to 70°C in a 70 % acetic acid solution (20 mL) for 45 min. The solvent was then removed by codistillation with toluene. Column chromatography (PE/EA 3:1 to 1:2) led to products **1-4g**, **1m** or **1q**.

2,3-*O*-Methyl-1-deoxy-D-ribofuranoside 1g

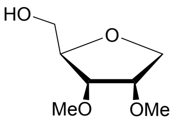

Yield: 511 mg (85 %); R_f = 0.16 (EA); $[\alpha]_D^{22}$ = 106.5° (c = 1.0, $CHCl_3$); ^1H NMR (500 MHz, $CDCl_3$): δ = 3.98 (dd, 1H, H-1a), 3.96–3.89 (m, 3H, H-1b, H-2, H-4), 3.85 (dd, 1H, H-5a), 3.78 (dd, 1H, H-3), 3.63 (dd, 1H, H-5b), 3.46 (s, 3H, CH_3), 3.44 (s, 3H, CH_3); ^{13}C NMR (125 MHz, $CDCl_3$): δ = 80.7, 78.9 (C-2, C-4), 80.3 (C-3), 70.2 (C-1), 62.4 (C-5), 58.1, 57.6 (2x CH_3); HRMS (ESI), m/z calc. for $C_7H_{14}NaO_4$ $[M+Na]^+$: 185.078, found: 185.079; Elemental Analysis for $C_7H_{14}O_4$: C: 51.84, H: 8.70, found: C: 51.98, H: 8.75.

2,3-*O*-Methyl-1-deoxy-D-lyxofuranoside 2g

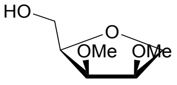

Yield: 511 mg (85 %); R_f = 0.18 (EA); $[\alpha]_D^{24}$ = -42.0° (c = 1.0, MeOH); ^1H NMR (300 MHz, $CDCl_3$): δ = 4.13–4.05 (m, 1H, H-4), 4.03–3.95 (m, 2H, H-2 or H-3, H-1a), 3.90 (dt, 1H, H-2 or H-3), 3.78 (dd, 1H, H-1b), 3.72 (d, 2H, H-5a, H-5b), 3.30 (s, 6H, 2x CH_3); ^{13}C NMR (75 MHz, DMSO-d_6): δ = 81.3, 79.0, 78.4 (C-2, C-3, C-4), 68.8 (C-1), 61.6 (C-5), 59.0, 57.7 (2x CH_3); HRMS (ESI), m/z calc. for $C_7H_{14}NaO_4$ $[M+Na]^+$: 185.078, found: 185.078; Elemental Analysis for $C_7H_{14}O_4$: C: 51.84, H: 8.70, found: C: 51.85, H: 8.97.

2,3-*O*-Methyl-1-deoxy-D-xylofuranoside 3g

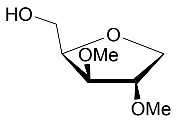

Yield: 511 mg (85 %); R_f = 0.24 (EA); $[\alpha]_D^{22}$ = -58.4° (c = 1.8, $CHCl_3$); ^1H NMR (500 MHz, DMSO-d_6): δ = 4.52 (t, 1H, OH), 3.92–3.88 (m, 1H, H-1a), 3.88–3.86 (m, 1H, H-2), 3.80–3.75 (m, 1H, H-4), 3.71 (dd, 1H, H-3), 3.57–3.52 (m, 2H, H-5a, H-1b), 3.47–3.42 (m, 1H, H-5b), 3.31 (s, 6H, 2x CH_3); ^{13}C NMR (125 MHz, DMSO-d_6): δ = 82.9, 82.8, (C-2, C-3), 81.2 (C-4), 70.2 (C-1), 58.8 (C-5), 57.1, 56.3 (2x CH_3); HRMS (ESI), m/z calc. for $C_7H_{14}NaO_4$ $[M+Na]^+$: 185.078, found: 185.079; Elemental Analysis for $C_7H_{14}O_4$: C: 51.84, H: 8.70, found: C: 51.22, H: 8.75.

2,3-*O*-Methyl-1-deoxy-L-arabinofuranoside 4g

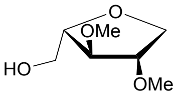

Yield: 511 mg (85 %); R_f = 0.19 (EA); $[\alpha]_D^{22}$ = -46.0° (c = 1.0, MeOH); ^1H NMR (500 MHz, DMSO-d_6): δ = 4.75 (t, 1H, OH), 3.82–3.76 (m, 2H, H-1a, H-3), 3.70 (dd, 1H, H-1b), 3.66–3.60 (m, 2H, H-2, H-4), 3.43–3.35 (m, 2H, H-5a, H-5b), 3.30 (s, 6H, 2x CH_3); ^{13}C NMR (125 MHz, DMSO-d_6): δ = 85.5, 84.2, 84.2 (C-2, C-3, C-4), 70.3 (C-1), 61.6 (C-5), 56.6, 56.1 (2x CH_3); HRMS (ESI), m/z calc. for $C_7H_{14}NaO_4$ $[M+Na]^+$: 185.078, found: 185.079; Elemental Analysis for $C_7H_{14}O_4$: C: 51.84, H: 8.70, found: C: 51.56, H: 8.56.

2,3-*O*-Ethyl-1-deoxy-D-ribofuranoside 1m

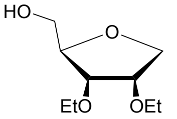

Yield: 639 mg (84 %); R_f = 0.20 (EA); $[\alpha]_D^{24}$ = 82.5° (c = 1.0, $CHCl_3$); ^1H NMR (300 MHz, $CDCl_3$): δ = 4.06–3.77 (m, 6H, H-1a, H-1b, H-2, H-3, H-4, H-5a), 3.73–3.46 (m, 5H, H-5b, 2x CH_2), 2.07 (s, 1H, OH), 1.23 (t, 6H, 2x CH_3); ^{13}C NMR (75 MHz, $CDCl_3$): δ = 80.9, 78.6, 77.3 (C-2, C-3, C-4), 71.2 (C-1), 65.9, 65.5 (2x CH_2), 62.2 (C-5), 15.3, 15.3 (2x CH_3); HRMS (ESI), m/z calc. for $C_9H_{18}NaO_4$ $[M+Na]^+$: 213.110, found: 213.110; Elemental Analysis for $C_9H_{18}O_4$: C: 56.82, H: 9.54, found: C: 56.61, H: 9.74.

2,3-*O*-Allyl-1-deoxy-D-ribofuranoside 1q

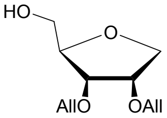

Yield: 754 mg (88 %); R_f = 0.22 (EA); $[\alpha]_D^{24}$ = 76.3° (c = 1.0, $CHCl_3$); ^1H NMR (250 MHz, $CDCl_3$): δ = 6.07–5.80 (m, 2H, 2x C*H*=CH_2), 5.36–5.17 (m, 4H, 2x CH=C*H*$_2$), 4.22–3.80 (m, 10H, H-1a, H-1b, H-2, H-3, H-4, H-5a, 2x OCH_2), 3.69–3.55 (m, 1H, H-5b), 1.98 (q, 1H, OH); ^{13}C NMR (63 MHz, $CDCl_3$): δ = 134.6, 134.5 (2x *C*H=CH_2), 117.5, 117.4 (2x CH=*C*H$_2$), 80.9, 77.9, 76.7 (C-2, C-3, C-4), 71.5, 71.0, 71.0 (C-1, 2x OCH_2), 62.2 (C-5); HRMS (ESI), m/z calc. for $C_{11}H_{18}NaO_4$ $[M+Na]^+$: 237.110, found: 237.110; Elemental Analysis for $C_{11}H_{18}O_4$: C: 61.66, H: 8.47, found: C: 61.56, H: 8.65.

Procedure for the reduction of 2,3-*O*-allyl-1-deoxy-D-ribofuranoside 1q to the corresponding propyl ether 1t

1q (321 mg, 1.5 mmol) and $Pd(OH)_2$ (20 mg) were dissolved in dry methanol (20 mL) and stirred at room temperature in a closed flask under H_2-atmosphere for 12 h. The reaction was filtered afterwards to yield product **1t**.

2,3-*O*-Propyl-1-deoxy-D-ribofuranoside 1t

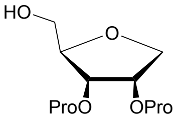

Yield: 295 mg (90 %); R_f = 0.34 (EA); $[\alpha]_D^{24}$ = 92.8° (c = 1.0, $CHCl_3$); ^1H NMR (300 MHz, $CDCl_3$): δ = 4.02–3.77 (m, 6H, H-1a, H-1b, H-2, H-3,H-4; H-5a), 3.65–3.38 (m, 5H, H-5b, 2x OCH_2), 2.07 (s, 1H, OH), 1.62 (m, 4H, CH_2), 0.93 (t, 6H, CH_3); ^{13}C NMR (75 MHz, $CDCl_3$): δ = 80.8, 78.9, 77.5 (C-2, C-3, C-4), 72.3, 72.0 (2x OCH_2), 71.2 (C-1), 62.3 (C-5), 23.0, 23.0 (2x CH_2), 10.5, 10.5 (2x CH_3); HRMS (ESI), m/z calc. for $C_{11}H_{22}NaO_4$ $[M+Na]^+$: 241.141, found: 241.141; Elemental Analysis for $C_{11}H_{22}O_4$: C: 60.52, H: 10.16, found: C: 60.34, H: 9.89.

General two-step procedure for the introduction of the 5-*O*-triflate group followed by quarternization with pyridine

1-4g, **1m**, **1q** or **1t** (4.5 mmol) was dissolved in dry dichlormethane (18 mL) and cooled to 0°C. Pyridine (1.07 mL, 13.3 mmol) was added. Afterwards, Tf_2O (1.5 mL, 9.0 mmol) was added dropwise and the mixture was stirred for 10 min at 0°C. Dichloromethane (20 mL) was added and the mixture was washed with cold water (15 mL), sat. $NaHCO_3$ (2x 15 mL) and cold water again (15 mL). The dried organic phase contains the 5-*O*-triflate intermediates **1-4h**, **1n**, **1r** or **1u**, which were directly used in the next step. Pyridine (2.15 mL, 26.6 mmol) was added and the follow-up reaction was performed at the rotary evaporator (40°C, 700 mbar). Further work-up is stated under the respective products **1-4i**, **1o**, **1s** or **1v**.

***N*-(2,3-*O*-Methyl-1,5-deoxy-D-ribofuranoside-5-yl)-pyridinium triflate 1i**

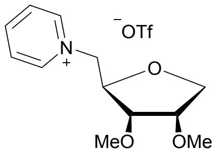

Work-up: The crude product was dissolved in dest. Water (100 mL) and washed with dichloromethane (4x 10 mL) and diethyl ether (2x 10 mL). The aqueous phase was evaporated. The product was then dissolved in dry methanol (10 mL) and a small amount of activated charcoal was added to further purify the product from remaining pyridine. The solvent was evaporated after filtration and the product was dried under high vaccum.

Yield: 1.27 g (82 %); m.p. = 48–51°C; R_f = 0 (EA); $[\alpha]_D^{24}$ = 68.2° (c = 1.0, MeOH); ^1H NMR (500 MHz, MeOD): δ = 8.97 (d, 2H, CH_{Pyr}), 8.64 (t, 1H, CH_{Pyr}), 8.13 (d, 2H, CH_{Pyr}), 4.92 (dd, 1H, $^3J_{H\text{-}5a,H\text{-}5b}$ = 13.56 Hz, H-5a), 4.71 (dd, 1H, H-5b), 4.18 (ddd, 1H, $^3J_{H\text{-}4,H\text{-}5b}$ = 5.36 Hz, H-4), 4.07 (ddd, 1H, H-2), 4.01 (dd, 1H, H-1a), 3.94 (d, 1H, H-1b), 3.73 (dd, 1H, H-3), 3.48 (s, 3H, CH_3), 3.42 (s, 3H, CH_3); ^{13}C NMR (75 MHz, MeOD): δ = 147.5, 146.8, 129.4 (CH_{Pyr}), 83.7 (C-3), 79.6, 79.4 (C-2, C-4), 71.9 (C-1), 64.8 (C-5), 58.7, 58.0 (2x CH_3); ^{19}F NMR (282 MHz, MeOD): δ = -80.06; HRMS (ESI), m/z calc. for $C_{12}H_{18}NO_3$ $[Cation]^+$: 224.128, found: 224.128, m/z calc. for CF_3O_3S $[Anion]^-$: 148.953, found: 148.953; Elemental Analysis for $C_{13}H_{18}F_3NO_6S$: C: 41.82, H: 4.86, N: 3.75, S: 8.59, found: C: 41.79, H: 4.69, N: 3.74, S: 8.69.

***N*-(2,3-*O*-Methyl-1,5-deoxy-D-lyxofuranoside-5-yl)-pyridinium triflate 2i**

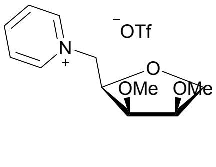

Work-up: The crude product was dissolved in dest. Water (100 mL) and washed with dichloromethane (4x 10 mL) and diethyl ether (2x 10 mL). The aqueous phase was evaporated. The product was then dissolved in dry methanol (10 mL) and a small amount of

activated charcoal was added to further purify the product from remaining pyridine. The solvent was evaporated after filtration and the product was dried under high vaccum.

Yield: 941 mg (63 %); liquid at room temperature; R_f = 0 (EA); $[\alpha]_D^{23}$ = 31.4° (c = 1.1, $CHCl_3$); ^1H NMR (300 MHz, MeOD): δ = 8.90–8.85 (CH_{Pyr}), 8.60–8.52 (m, 1H, CH_{Pyr}), 8.08–8.00 (m, 2H, CH_{Pyr}), 4.79–4.74 (m, 2H, H-5a, H-5b), 4.52–4.44 (m, 1H, H-4), 4.23 (dd, 1H, H-3), 4.01 (dd, 1H, H-1a), 3.96–3.91 (m, 1H, H-2), 3.76 (dd, 1H, H-1b), 3.50 (s, 3H, CH_3), 3.20 (s, 3H, CH_3); ^{13}C NMR (75 MHz, MeOD): δ = 147.7, 146.8, 128.5 (CH_{Pyr}), 82.8 (C-3), 79.6 (C-2), 77.8 (C-4), 70.8 (C-1), 62.9 (C-5), 59.2, 58.1 (2x CH_3); ^{19}F NMR (282 MHz, MeOD): δ = -80.06; HRMS (ESI), m/z calc. for $C_{12}H_{18}NO_3$ [Cation]$^+$: 224.128, found: 224.128, m/z calc. for CF_3O_3S [Anion]$^-$: 148.953, found: 148.952; Elemental Analysis for $C_{13}H_{18}F_3NO_6S$: C: 41.82, H: 4.86, N: 3.75, S: 8.59, found: C: 41.73, H: 4.95, N: 3.82, S: 8.76.

***N*-(2,3-*O*-Methyl-1,5-deoxy-D-xylofuranoside-5-yl)-pyridinium triflate 3i**

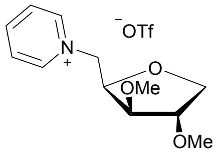

Work-up: The crude product was dissolved in dest. Water (100 mL) and washed with dichloromethane (4x 10 mL) and diethyl ether (2x 10 mL). The aqueous phase was evaporated. The product was then dissolved in dry methanol (10 mL) and a small amount of activated charcoal was added to further purify the product from remaining pyridine. The solvent was evaporated after filtration and the product was dried under high vaccum.

Yield: 1.27 g (85 %); m.p. = 32–36°C; R_f = 0 (EA); $[\alpha]_D^{27}$ = -2.3° (c = 1.1, MeOH); ^1H NMR (500 MHz, MeOD): δ = 9.06–8.97 (m, 2H, CH_{Pyr}), 8.70–8.60 (m, 1H, CH_{Pyr}), 8.19–8.09 (m, 2H, CH_{Pyr}), 4.98 (dd, 1H, $^3J_{H\text{-}5a,H\text{-}5b}$ = 13.60 Hz, H-5a), 4.81 (dd, 1H, H-5b), 4.44–4.37 (m, 1H, H-4), 4.17–4.10 (m, 1H, H-1a), 4.09–4.04 (m, 2H, H-2, H-3), 3.83 (dd, 1H, H-1b), 3.51 (s, 3H, CH_3), 3.44 (s, 3H, CH_3); ^{13}C NMR (75 MHz, MeOD): δ = 147.3, 147.0, 129.2 (CH_{Pyr}), 85.3, 84.4 (C-2, C-3), 80.3 (C-4), 72.6 (C-1), 62.6 (C-5), 58.2, 57.4 (2x CH_3); ^{19}F NMR (282 MHz, MeOD): δ = -80.06; HRMS (ESI), m/z calc. for $C_{12}H_{18}NO_3$ [Cation]$^+$: 224.128, found: 224.128, m/z calc. for CF_3O_3S [Anion]$^-$: 148.953, found: 148.953; Elemental Analysis for $C_{13}H_{18}F_3NO_6S$: C: 41.82, H: 4.86, N: 3.75, S: 8.59, found: C: 41.87, H: 4.96, N: 3.64, S: 8.31.

***N*-(2,3-*O*-Methyl-1,5-deoxy-L-arabinofuranoside-5-yl)-pyridinium triflate 4i**

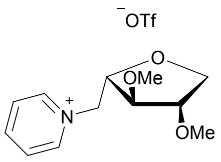

Work-up: The crude product was dissolved in dest. Water (100 mL) and washed with dichloromethane (4x 10 mL) and diethyl ether (2x 10 mL). The aqueous phase was evaporated. The product was then dissolved in dry methanol (10 mL) and a small amount of activated charcoal was added to further purify the product from remaining pyridine. The solvent was evaporated after filtration and the product was dried under high vaccum.

Yield: 1.34 g (90 %); liquid at room temperature; R_f = 0 (EA); $[\alpha]_D^{23}$ = -97.0° (c = 1.0, MeOH); ^{1}H NMR (500 MHz, MeOD): δ = 8.94 (d, 2H, CH_{Pyr}), 8.62 (t, 1H, CH_{Pyr}), 8.10 (t, 2H, CH_{Pyr}), 4.92 (dd, 1H, $^3J_{H\text{-}5a,H\text{-}5b}$ = 13.60 Hz, H-5a), 4.75 (dd, 1H, H-5b), 4.28–4.20 (m, 1H, $^3J_{H\text{-}4,H\text{-}5a}$ = 10.39 Hz, H-4), 4.04 (d, 1H, H-1a), 3.90–3.77 (m, 3H, H-1b, H-2, H-3), 3.46 (s, 3H, CH_3), 3.24 (s, 3H, CH_3); ^{13}C NMR (125 MHz, MeOD): δ = 147.3, 147.1, 129.1 (CH_{Pyr}), 87.1, 84.6 (C-2, C-3), 83.4 (C-4), 72.9 (C-1), 64.1 (C-5), 58.1, 57.2 (2x CH_3); ^{19}F NMR (282 MHz, MeOD): δ = -80.07; HRMS (ESI), m/z calc. for $C_{12}H_{18}NO_3$ [Cation]$^+$: 224.128, found: 224.128, m/z calc. for CF_3O_3S [Anion]$^-$: 148.953, found: 148.953; Elemental Analysis for $C_{13}H_{18}F_3NO_6S$: C: 41.82, H: 4.86, N: 3.75, S: 8.59, found: C: 41.48, H: 4.98, N: 3.69, S: 8.43.

***N*-(2,3-*O*-Ethyl-1,5-deoxy-D-ribofuranoside-5-yl)-pyridinium triflate 1o**

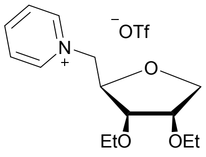

Work-up: The crude product was dissolved in dest. Water (100 mL) and washed with dichloromethane (4x 10 mL) and diethyl ether (2x 10 mL). The aqueous phase was evaporated. The product was then dissolved in dry methanol (10 mL) and a small amount of activated charcoal was added to further purify the product from remaining pyridine. The solvent was evaporated after filtration and the product was dried under high vaccum.

Yield: 1.17 g (72 %); liquid at room temperature; R_f = 0 (EA); $[\alpha]_D^{24}$ = 61.7° (c = 1.0, MeOH); ^{1}H NMR (500 MHz, MeOD): δ = 8.97 (d, 2H, CH_{Pyr}), 8.64 (t, 1H, CH_{Pyr}), 8.13 (d, 2H, CH_{Pyr}), 4.91 (dd, 1H, $^3J_{H\text{-}5a,H\text{-}5b}$ = 13.56 Hz, H-5a), 4.72 (dd, 1H, H-5b), 4.20 (dt, 1H, $^3J_{H\text{-}4,H\text{-}5b}$ = 5.04 Hz, H-4), 4.13 (dt, 1H, H-2), 4.04 (dd, 1H, H-1a), 3.90 (dd, 1H, H-1b), 3.80 (dd, 1H, H-3), 3.75–3.67, 3.66–3.55 (m, 4H, 2x CH_2), 1.24 (t, 3H, CH_3), 1.20 (t, 3H, CH_3); ^{13}C NMR (75 MHz, MeOD): δ = 147.5, 146.8, 129.4 (CH_{Pyr}), 82.3 (C-3), 79.7 (C-4), 78.1 (C-2), 72.8 (C-

1), 67.2, 66.7 (2x CH_2), 64.9 (C-5), 15.8, 15.7 (2x CH_3); ^{19}F NMR (282 MHz, MeOD): δ = -80.05; HRMS (ESI), m/z calc. for $C_{14}H_{22}NO_3$ [Cation]$^+$: 252.159, found: 252.160, m/z calc. for CF_3O_3S [Anion]$^-$: 148.953, found: 148.952; Elemental Analysis for $C_{15}H_{22}F_3NO_6S$: C: 44.88, H: 5.52, N: 3.49, S: 7.99, found: C: 44.52, H: 5.49, N: 3.42, S: 7.92.

***N*-(2,3-*O*-Allyl-1,5-deoxy-D-ribofuranoside-5-yl)-pyridinium triflate 1s**

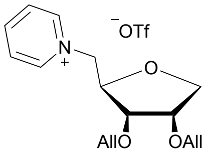

Work-up: The crude product was dissolved in dest. Water (100 mL) and washed with diethyl ether (2x 10 mL). The aqueous phase was evaporated. The product was then dissolved in dry methanol (10 mL) and a small amount of activated charcoal was added to further purify the product from remaining pyridine. The solvent was evaporated after filtration and the product was dried under high vaccum.

Yield: 1.11 g (64 %); liquid at room temperature; R_f = 0 (EA); $[\alpha]_D^{25}$ = 70.4° (c = 1.1, MeOH); ^{1}H NMR (300 MHz, MeOD): δ = 9.01–8.94 (m, 2H, CH_{Pyr}), 8.63 (t, 1H, CH_{Pyr}), 8.12 (t, 2H, CH_{Pyr}), 6.05–5.85 (m, 2H, 2x C*H*=CH_2), 5.39–5.26 (m, 2H, CH=CH_2), 5.26–5.14 (m, 2H, CH=CH_2), 4.93 (dd, 1H, $^3J_{H\text{-}5a,H\text{-}5b}$ = 13.41 Hz, H-5a), 4.72 (dd, 1H, $^3J_{H\text{-}4,H\text{-}5b}$ = 8.31 Hz, H-5b), 4.27 (ddd, 1H, $^3J_{H\text{-}4,H\text{-}5a}$ = 3.02 Hz, H-4), 4.24–4.01 (m, 6H, H-1a, H-2, 2x OCH_2), 3.93 (dd, 1H, H-1b), 3.86 (dd, 1H, H-3); ^{13}C NMR (75 MHz, MeOD): δ = 147.5, 146.8 (CH_{Pyr}), 136.0, 135.6 (2x *C*H=CH_2), 129.4 (CH_{Pyr}), 118.4, 117.7 (2x CH=*C*H_2), 81.8 (C-3), 79.7 (C-4), 77.6 (C-2), 72.8, 72.7 (2x OCH_2), 72.2 (C-1), 64.8 (C-5); ^{19}F NMR (282 MHz, MeOD): δ = -80.06; HRMS (ESI), m/z calc. for $C_{16}H_{22}NO_3$ [Cation]$^+$: 276.159, found: 276.160, m/z calc. for CF_3O_3S [Anion]$^-$: 148.953, found: 148.952; Elemental Analysis for $C_{17}H_{22}F_3NO_4S$: C: 48.00, H: 5.21, N: 3.29, S: 7.54, found: C: 47.42, H: 5.10, N: 3.44, S: 7.59.

***N*-(2,3-*O*-Propyl-1,5-deoxy-D-ribofuranoside-5-yl)-pyridinium triflate 1v**

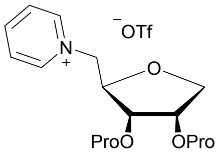

Work-up: The crude product was dissolved in dest. Water (100 mL) and washed with diethyl ether (2x 10 mL). The aqueous phase was evaporated. The product was then dissolved in dry methanol (10 mL) and a small amount of activated charcoal was added to further purify

the product from remaining pyridine. The solvent was evaporated after filtration and the product was dried under high vaccum.

Yield: 1.12 g (65 %); liquid at room temperature; R_f = 0 (EA); $[\alpha]_D^{22}$ = 54.5° (c = 1.0, MeOH); ^{1}H NMR (300 MHz, MeOD): δ = 9.00–8.94 (m, 2H, CH_{Pyr}), 8.63 (t, 1H, CH_{Pyr}), 8.13 (t, 2H, CH_{Pyr}), 4.91 (dd, 1H, $^3J_{H-4,H-5a}$ = 3.21 Hz, H-5a), 4.71 (dd, 1H, $^3J_{H-5a,H-5b}$ = 13.41 Hz, H-5b), 4.22 (dt, 1H, H-4), 4.11 (dt, 1H, $^3J_{H-2,H-3}$ = 8.12 Hz, H-2), 4.03 (dd, 1H, $^3J_{H-1a,H-1b}$ = 10.01 Hz, H-1a), 3.91 (dd, 1H, H-1b), 3.78 (dd, 1H, H-3), 3.68–3.42 (m, 4H, 2x OCH_2), 1.72–1.52 (m, 4H, 2x CH_2), 0.97 (t, 3H, CH_3), 0.94 (t, 3H, CH_3); ^{13}C NMR (75 MHz, MeOD): δ = 147.4, 146.6, 129.2 (CH_{Pyr}), 82.4 (C-3), 79.5 (C-4), 78.1 (C-2), 73.3, 72.9 (2x OCH_2), 72.6 (C-1), 64.8 (C-5), 24.1, 24.1 (2x CH_2), 10.9, 10.9 (2x CH_3); ^{19}F NMR (282 MHz, MeOD): δ = -80.07; HRMS (ESI), m/z calc. for $C_{16}H_{26}NO_3$ [Cation]$^+$: 280.191, found: 280.191, m/z calc. for CF_3O_3S [Anion]$^-$: 148.953, found: 148.953; Elemental Analysis for $C_{17}H_{26}F_3NO_4S$: C: 47.54, H: 6.10, N: 3.26, S: 7.47, found: C: 45.97, H: 6.08, N: 3.20, S: 7.54.

Procedure for synthesis of 2,3-*O*-Isopropylidene-1-deoxy-D-ribofuranoside 1j

1d (872 mg, 6.5 mmol) was suspended in dry acetone (6.0 mL). Dimethoxy propane (1.6 mL, 13.0 mmol) and camphersulfonic acid (154 mg, 0.66 mmol) were added and the reaction was stirred at room temperature for 1.5 h. Afterwards methanol (12 mL) and sat. $NaHCO_3$ (2 mL) were added and the mixture was evaporated. Column chromatography (PE/EA 1:1, 1 % NEt_3) led to product **1j**.

2,3-*O*-Isopropylidene-1-deoxy-D-ribofuranoside 1j

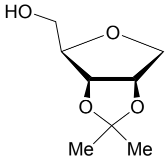

Yield: 1.09 g (96 %); R_f = 0.29 (PE/EA 1:1); $[\alpha]_D^{22}$ = 37.5° (c = 1.0, $CHCl_3$); ^{1}H NMR (300 MHz, $CDCl_3$): δ = 4.86–4.81 (m, 1H, H-2), 4.67 (dd, 1H, H-3), 4.01 (dt, 1H, H-4), 3.95 (dd, 1H, H-1a), 3.88 (dd, 1H, H-1b), 3.60–3.48 (m, 2H, H-5a, H-5b), 1.46 (s, 3H, CH_3), 1.32 (s, 3H, CH_3); ^{13}C NMR (75 MHz, $CDCl_3$): δ = 113.6 ($C_{isopropylidene}$), 86.8 (C-4), 83.8 (C-3), 82.7 (C-2), 74.2 (C-1), 62.5 (C-5), 27.0, 25.2 (2x CH_3); HRMS (ESI), m/z calc. for $C_8H_{14}NaO_4$ [M+Na]$^+$: 197.078, found: 197.079; Elemental Analysis for $C_8H_{14}O_4$: C: 55.16, H: 8.10, found: C: 55.36, H: 8.14.

Procedure for the introduction of the 5-*O*-triflate group, followed by quarternization with pyridine, followed by 2,3-*O*-isopropylidene deprotection, leading to product 1k

1j (784 mg, 4.5 mmol) was dissolved in dry dichlormethane (18 mL) and cooled to 0°C. Pyridine (1.07 mL, 13.3 mmol) was added. Afterwards, Tf_2O (1.5 mL, 9.0 mmol) was added dropwise and the mixture was stirred for 10 min at 0°C. Dichloromethane (20 mL) was added and the mixture was washed with cold water (15 mL), sat. $NaHCO_3$ (2x 15 mL) and cold water again (15 mL). The dried organic phase contains a mixture of the 5-*O*-triflate intermediates, both 2,3-*O*-isopropylidene protected and unprotected. The mixture was directly used in the next step. Pyridine (2.15 mL, 26.6 mmol) was added and the follow-up reaction was performed at the rotary evaporator (40°C, 700 mbar). The crude product was dissolved in dest. water (100 mL) and washed with dichloromethane (4x 10 mL) and diethyl ether (2x 10 mL). The aqueous phase was evaporated. The product was then dissolved in dry methanol (10 mL) and a small amount of activated charcoal was added to further purify the product from remaining pyridine. The solvent was evaporated and the product was dried under high vaccum. This leads to a mixture of both 2,3-*O*-isopropylidene protected and unprotected pyridinium triflate salts. To achieve pure product **1k**, a 70 % acetic acid solution (3 mL) was added and the reaction was stirred for 20 min. The mixture was codistilled with toluene until all acetic acid is removed. The crude product was again dissolved in dest. water (100 mL) and washed with dichloromethane (3x 20 mL). The aqueous phase was evaporated. The product was then dissolved in dry methanol (10 mL) and a small amount of activated charcoal was added. The solvent was evaporated after filtration and the product was dried under high vaccum, leading to **1k**.

***N*-(1,5-deoxy-D-ribofuranoside-5-yl)-pyridinium triflate 1k**

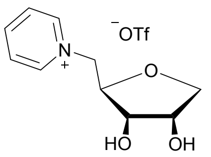

Yield: 1.01 g (65 %); liquid at room temperature; R_f = 0 (EA); $[\alpha]_D^{22}$ = 35.8° (c = 1.0, MeOH); ^{1}H NMR (300 MHz, MeOD): δ = 9.00–8.93 (m, 2H, CH_{Pyr}), 8.67–8.59 (m, 1H, CH_{Pyr}), 8.17–8.07 (m, 2H, CH_{Pyr}), 4.93 (dd, 1H, $^3J_{H\text{-}5a,H\text{-}5b}$ = 13.41 Hz, H-5a), 4.71 (dd, 1H, H-5b), 4.18 (dt, 1H, H-2), 4.13–4.05 (m, 2H, H-1b, H-4), 3.90 (dd, 1H, H-3), 3.75 (dd, 1H, H-1b); ^{13}C NMR (63 MHz, MeOD): δ = 147.4, 146.8, 129.4 (CH_{Pyr}), 80.8 (C-4), 75.1 (C-3), 74.9 (C-1), 72.3 (C-2), 64.8 (C-5); ^{19}F NMR (282 MHz, MeOD): δ = -80.04; HRMS (ESI), m/z calc. for $C_{10}H_{14}NO_3$ [Cation]$^+$: 196.097, found: 196.097, m/z calc. for CF_3O_3S [Anion]$^-$: 148.953, found: 148.952;

Elemental Analysis for $C_{11}H_{14}NO_6S$: C: 38.26, H: 4.09, N: 4.06, S: 9.29, found: C: 38.14, H: 4.13, N: 3.98, S: 8.94.

Procedure for synthesis of 2,3-*O*-Isopropylidene-5-*O*-mesyl-1-deoxy-D-ribofuranoside 1w

1j (610 mg, 3.5 mmol) was dissolved pyridine (20 mL) and mesyl chloride (0.39 mL, 5.0 mmol) was added. The reaction was stirred at room temperature overnight. Dichloromethane (100 mL) was added and the mixture was washed with cold water (20 mL), 15 % aqueous $NaHSO_4$ solution (3x 20 mL) and cold water again (20 mL). Column chromatography (PE/EA 2:1) led to product **1w**.

2,3-*O*-Isopropylidene-5-*O*-mesyl-1-deoxy-D-ribofuranoside 1w

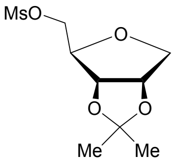

Yield: 759 mg (86 %); m.p. = 78–80°C; R_f = 0.48 (PE/EA 1:1); $[\alpha]_D^{22}$ = 39.5° (c = 1.0, $CHCl_3$); ^1H NMR (250 MHz, $CDCl_3$): δ = 4.90–4.80 (m, 1H, H-2), 4.67 (d, 1H, H-3), 4.35 (m, 3H, H-4, H-5a, H-5b), 4.09–3.94 (m, 2H, H-1a, H-1b), 3.07 (s, 3H, SO_2CH_3), 1.53 (s, 3H, CH_3), 1.35 (s, 3H, CH_3); ^{13}C NMR (63 MHz, $CDCl_3$): δ = 113.3 ($C_{isopropylidene}$), 82.3 (C-4), 81.8 (C-3), 81.0 (C-2), 73.6 (C-1), 68.6 (C-5), 37.7 (SO_2CH_3), 26.6, 25.0 (2x CH_3); HRMS (ESI), m/z calc. for $C_9H_{16}NaO_6S$ $[M+Na]^+$: 275.056, found: 275.056; Elemental Analysis for $C_9H_{16}O_6S$: C: 42.85, H: 6.39, S: 12.71, found: C: 42,91, H: 6.28, S: 12.68.

Procedure for the quarternization of the 5-*O*-mesylate 1w

1w (757 mg, 3.0 mmol) was dissolved in dry pyridine (5 mL) and heated at 125°C for 5 h. The crude product was dissolved in dest. water (100 mL) and washed with dichloromethane (4x 10 mL) and diethyl ether (2x 10 mL). The aqueous phase was evaporated. The product was then dissolved in dry methanol (10 mL) and a small amount of activated charcoal was

added to further purify the product from remaining pyridine. The solvent was evaporated after filtration and the product **1x** was dried under high vaccum.

***N*-(2,3-*O*-Isopropylidene-1,5-deoxy-D-ribofuranoside-5-yl)-pyridinium mesylate 1x**

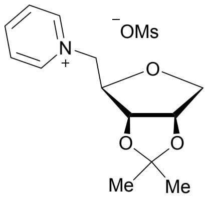

Yield: 845 mg (85 %); m.p. = 92–94°C; R_f = 0 (EA); $[\alpha]_D^{21}$ = 99.0° (c = 1.0, MeOH); ^{1}H NMR (250 MHz, MeOD): δ = 8.95 (d, 2H, CH_{Pyr}), 8.70–8.57 (m, 1H, CH_{Pyr}), 8.14 (t, 2H, CH_{Pyr}), 4.97 (dd, 1H, $^3J_{H-2,H-3}$ = 6.15 Hz, H-2), 4.89–4.79 (m, 1H, H-5a), 4.77 (dd, 1H, H-3), 4.65 (t, 1H, H-5b), 4.46–4.36 (m, 1H, H-4), 4.18 (dd, 1H, $^3J_{H-1a,H-2}$ = 3.78 Hz, H-1a), 4.01 (pd, 1H, H-1b), 2.70 (s, 3H, SO_3CH_3), 1.45 (s, 3H, CH_3), 1.35 (s, 3H, CH_3); ^{13}C NMR (75 MHz, $CDCl_3$): δ = 147.4, 146.7, 129.5 (CH_{Pyr}), 114.4 ($C_{isopropylidene}$), 85.5 (C-4), 83.9 (C-3), 82.5 (C-2), 73.2 (C-1), 61.2 (C-5), 39.7 (SO_3CH_3), 26.9, 25.2 (2x CH_3); HRMS (ESI), m/z calc. for $C_{13}H_{18}NO_3$ [Cation]$^+$: 236.128, found: 236.128, m/z calc. for CH_3O_3S [Anion]$^-$: 94.981, found: 94.981; Elemental Analysis for $C_{14}H_{21}NO_6S$: C: 49.40, H: 6.51, N: 4.11, S: 9.42, found: C: 49.27, H: 6.26, N: 4.10, S: 9.22.

General procedure for synthesis of 6-*O*-trityl-glucopyranosides 5-8b.

Methyl-β-D-glucopyranoside **5a**, allyl-β-D-glucopyranoside **6a**, phenyl-β-D-glucopyranoside **7a** or methyl-α-D-glucopyranoside **8a** (10.0 mmol), trityl chloride (4.18 g, 15.0 mmol), DMAP (one spatula tip) and NEt_3 (6.7 mL) were stirred at room temperature overnight in dichloromethane (25 mL). The reaction mixture was evaporated. Column chromatography (PE/EA 4:1 to EA) led to products **5-8b**.

Methyl-6-*O*-trityl-β-D-glucopyranoside 5b

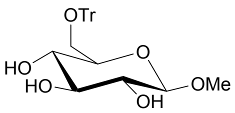

Yield: 3.80 g (87 %); m.p. = 108–109°C; R_f = 0.25 (EA); $[\alpha]_D^{25}$ = -61.3° (c = 1.0, $CHCl_3$); ^{1}H NMR (300 MHz, DMSO-d_6): δ = 7.46–7.21 (m, 15H, CH_{Ar}), 5.09 (d, 1H, OH), 4.95 (d, 1H, OH), 4.84 (d, 1H, OH), 4.14 (d, 1H, H-1), 3.49 (s, 3H, CH_3), 3.39–3.22 (m, 2H, H-6a, H-6b), 3.16–2.95 (m, 4H, H-2, H-3, H-4, H-5); ^{13}C NMR (75 MHz, DMSO-d_6): δ = 144.0 (C_{Ar}), 128.3,

127.8, 126.9 (CH_{Ar}), 103.80 (C-1), 85.5 (*C*Ph_3), 76.8, 75.1, 73.4, 70.22 (C-2, C-3, C-4, C-5), 63.6 (C-6), 55.6 (CH_3); HRMS (ESI), m/z calc. for $C_{26}H_{28}NaO_6$ $[M+Na]^+$: 459.178, found: 459.178; Elemental Analysis for $C_{26}H_{28}O_6$: C: 71.54, H: 6.47, found: C: 71.32, H: 6.69.

Allyl-6-*O*-trityl-β-D-glucopyranoside 6b

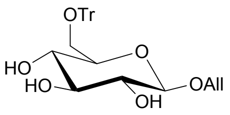

Yield: 3.05 g (66 %); R_f = 0.49 (EA); $[\alpha]_D^{23}$ = 41.7° (c = 1.0, $CHCl_3$); ^1H NMR (500 MHz, $CDCl_3$): δ = 7.47–7.22 (m, 15H, CH_{Ar}), 6.07–5.96 (m, 1H, C*H*=CH_2), 5.37 (d, 1H, CH=C*H_2*), 5.19 (d, 1H, CH=C*H_2*), 5.09 (s, 1H, OH), 4.95 (s, 1H, OH), 4.82 (s, 1H, OH), 4.36 (d, 1H, OCH_2), 4.28 (t, 1H, H-1), 4.20 (dd, 1H, OCH_2), 3.38–3.29 (m, 1H, H-5), 3.29–3.23 (m, 1H, H-6a), 3.18–3.00 (m, 4H, H-2, H-3, H-4, H-6b); ^{13}C NMR (125 MHz, $CDCl_3$): δ = 144.0 (C_{Ar}), 135.0 (*C*H=CH_2), 128.3, 127.7, 126.9 (CH_{Ar}), 116.6 (CH=*C*H_2), 102.1 (C-1), 85.5 (*C*Ph_3), 76.9, 73.4, 70.2 (C-2, C-3, C-4), 75.2 (C-5), 68.9 (OCH_2), 63.6 (C-6); HRMS (ESI), m/z calc. for $C_{28}H_{30}NaO_6$ $[M+Na]^+$: 485.193, found: 485.193; Elemental Analysis for $C_{28}H_{30}O_6$: C: 72.71, H: 6.54, found: C: 72.54, H: 6.58.

Phenyl-6-*O*-trityl-β-D-glucopyranoside 7b

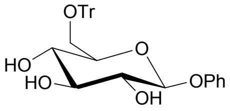

Yield: 4.25 g (83 %); R_f = 0.45 ($CHCl_3$/MeOH 10:1); $[\alpha]_D^{23}$ = -51.2° (c = 1.0, $CHCl_3$); ^1H NMR (300 MHz, DMSO-*d_6*): δ = 7.44–7.02 (m, 20H, CH_{Ar}), 5.35 (d, 1H, OH), 5.09 (d, 1H, OH), 4.96 (d, 1H, OH), 4.99 (d, 1H, H-1), 3.66 (t, 1H, H-5), 3.33–3.24 (m, 3H, H-2, H-3, H-6a), 3.06–2.98 (m, 2H, H-4, H-6b); ^{13}C NMR (75 MHz, DMSO-*d_6*): δ = 157.2, 143.8 (C_{Ar}); 129.6, 126.8, 128.3, 127.7, 121.6, 116.3 (CH_{Ar}) 100.0 (C-1), 85.6 (*C*Ph_3), 76.8, 73.2, (C-2, C-3), 75.2 (C-5), 70.3 (C-4), 63.7 (C-6); HRMS (ESI), m/z calc. for $C_{31}H_{30}NaO_6$ $[M+Na]^+$: 521.193, found: 521.194; Elemental Analysis for $C_{31}H_{30}O_6$: C: 74.68, H: 6.07, found: C: 74.82, H: 6.36.

Methyl-6-*O*-trityl-α-D-glucopyranoside 8b

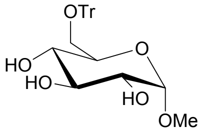

Yield: 3.71 g (85 %); m.p. = 152–154°C; R_f = 0.35 (EA); $[\alpha]_D^{23}$ = 74.2° (c = 1.0, $CHCl_3$); ^1H NMR (500 MHz, DMSO-d_6): δ = 7.43–7.22 (m, 15H, CH_{Ar}), 4.81 (d, 1H, OH), 4.75 (d, 1H, OH), 4.72 (d, 1H, OH), 4.62 (d, 1H, $^3J_{H-1,H-2}$ = 3.47 Hz, H-1), 3.62 (t, 1H, H-5), 3.41 (s, 3H, CH_3), 3.39–3.34 (m, 1H, H-3), 3.29–3.20 (m, 2H, H-2, H-6a), 3.05–2.94 (m, 2H, H-4, H-6b); ^{13}C NMR (125 MHz, DMSO-d_6): δ = 144.0 (C_{Ar}), 128.3, 127.8, 126.9 (CH_{Ar}), 99.6 (C-1), 85.6 (CPh_3), 73.6 (C-3), 71.9 (C-2), 70.9 (C-5), 70.7 (C-4), 63.8 (C-6), 54.1 (CH_3); HRMS (ESI), m/z calc. for $C_{26}H_{28}NaO_6$ $[M+Na]^+$: 459.178, found: 459.178; Elemental Analysis for $C_{26}H_{28}O_6$: C: 71.54, H: 6.47, found: C: 71.31, H: 6.48.

General procedure for synthesis of 6-*O*-trityl-2,3,4-*O*-methyl-glucopyranosides 5-8c and ethyl ether 5g

5-8b (5.5 mmol) was dissolved in dry DMF (33 mL) and cooled to 0°C. NaH (60 % dispersion in mineral oil, 1.3 eq per OH group) was added in small portions. The reaction was stirred at 0°C for 30 min, then methyl iodide (2.0 eq per OH group) was added and the reaction was stirred over night at room temperature. The solvent was evaporated, dichlormethane (100 mL) was added and the mixture was washed (3x 30 mL). Column chromatography (PE/EA 3:1) led to products **5-8c**.

For ethylation, **5b** and ethyl bromide (33.0 mmol, 2.46 mL) was used, leading to product **5g**.

Methyl-6-*O*-trityl-2,3,4-*O*-methyl-β-D-glucopyranoside 5c

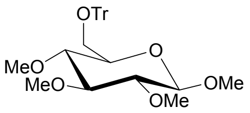

Yield: 2.45 g (93 %); R_f = 0.70 (EA); $[\alpha]_D^{25}$ = 50.1° (c = 1.0, $CHCl_3$); ^1H NMR (300 MHz, $CDCl_3$): δ = 7.43–7.07 (m, 15H, CH_{Ar}), 4.09 (d, 1H, H-1), 3.51 (s, 3H, CH_3), 3.50 (s, 3H, CH_3), 3.50 (s, 3H, CH_3), 3.34 (dd, 1H, $^3J_{H-4,H-5}$ = 2.08 Hz, H-5), 3.28 (d, 1H, H-6), 3.20 (s, 3H, CH_3), 3.15–3.09 (m, 1H, H-4), 3.03–2.94 (m, 3H, H-2, H-3, H-6); ^{13}C NMR (75 MHz, $CDCl_3$): δ = 144.0 (C_{Ar}), 128.7, 127.7, 126.9 (CH_{Ar}), 104.1 (C-1), 86.63 (CPh_3), 86.6, 83.8, 74.4 (C-2, C-3, C-4), 79.7 (C-5), 62.3 (C-6), 60.8, 60.5, 60.4, 55.6 (4x CH_3); HRMS (ESI), m/z calc. for $C_{29}H_{34}NaO_6$ $[M+Na]^+$: 501.225, found: 501.225; Elemental Analysis for $C_{29}H_{34}O_6$: C: 72.78, H: 7.16, found: C: 72.49, H: 7.24.

Allyl-6-*O*-trityl-2,3,4-*O*-methyl-β-D-glucopyranoside 6c

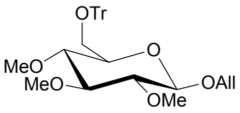

Yield: 2.47 g (89 %); R_f = 0.74 (PE/EA 3:1); $[\alpha]_D^{24}$ = 9.6° (c = 1.0, $CHCl_3$); ^{1}H NMR (500 MHz, DMSO-d_6): δ = 7.46–7.25 (m, 15H, CH_{Ar}), 6.06–5.97 (m, 1H, C*H*=CH_2), 5.35 (dd, 1H, CH=CH_2), 5.21 (dd, 1H, CH=CH_2), 4.40 (d, 1H, $^3J_{H-1,H-2}$ = 7.88 Hz, H-1), 4.39–4.34 (m, 1H, OCH_2), 4.16 (dd, 1H, OCH_2), 3.49 (s, 3H, CH_3), 3.48 (s, 3H, CH_3), 3.35–3.31 (m, 1H, H-5), 3.28–3.21 (m, 2H, H-4, H-6a), 3.19 (s, 3H, CH_3), 3.11 (t, 1H, $^3J_{H-2,H-3}$ = 8.83Hz, H-3), 3.00 (t, 1H, H-2), 2.94 (dd, 1H, H-6b); ^{13}C NMR (125 MHz, DMSO-d_6): δ = 143.7 (C_{Ar}), 134.7 (*C*H=CH_2), 128.2, 127.8, 127.0 (CH_{Ar}), 116.5 (CH=*C*H_2), 101.6 (C-1), 85.5 (*C*Ph_3), 85.4 (C-3), 83.3 (C-2), 79.1 (C-4), 73.2 (C-5), 68.9 (OCH_2), 62.1 (C-6), 59.9, 59.7, 59.4 (3x CH_3); HRMS (ESI), m/z calc. for $C_{31}H_{36}NaO_6$ $[M+Na]^+$: 527.240, found: 527.241; Elemental Analysis for $C_{31}H_{36}O_6$: C: 73.79, H: 7.19, found: C: 73.58, H: 7.11.

Phenyl-6-*O*-trityl-2,3,4-*O*-methyl-β-D-glucopyranoside 7c

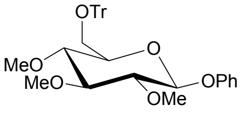

Yield: 2.59 g (85 %); R_f = 0.62 (PE/EA 1:1); $[\alpha]_D^{25}$ = -15.9° (c = 1.0, $CHCl_3$); ^{1}H NMR (300 MHz, DMSO-d_6): δ = 7.45–7.00 (m, 20H, CH_{Ar}), 5.14 (d, 1H, H-1), 3.69 (dd, 1H, H-5), 3.58 (s, 3H, CH_3), 3.51 (s, 3H, CH_3), 3.18 (s, 3H, CH_3), 3.29–3.21 (m, 4H, H-2, H-3, H-5, H-6a), 2.97 (dd, 1H, H-6b); ^{13}C NMR (75 MHz, DMSO-d_6): δ = 156.8, 143.6 (C_{Ar}), 129.4, 128.2, 127.8, 127.0, 122.2, 116.4 (CH_{Ar}), 99.6 (C-1), 85.7 (*C*Ph_3), 85.2, 83.0, 79.2, 73.3 (C-2, C-3, C-4, C-5), 62.3 (C-6), 59.9, 59.8, 59.4 (3x CH_3); HRMS (ESI), m/z calc. for $C_{34}H_{36}NaO_6$ $[M+Na]^+$: 563.240, found: 563.240; Elemental Analysis for $C_{34}H_{36}O_6$: C: 75.53, H: 6.71, found: C: 75.26, H: 6.88.

Methyl-6-*O*-trityl-2,3,4-*O*-methyl-α-D-glucopyranoside 8c

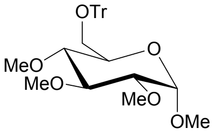

Yield: 2.47 g (94 %); R_f = 0.73 (PE/EA 1:1); $[\alpha]_D^{23}$ = 94.2° (c = 1.0, $CHCl_3$); ^{1}H NMR (300 MHz, DMSO-d_6): δ = 7.45–7.22 (m, 15H, CH_{Ar}), 4.94 (d, 1H, $^3J_{H-1,H-2}$ = 3.21 Hz, H-1), 3.50 (dd, 1H, H-3 or H-4), 3.43 (s, 3H, CH_3), 3.37 (s, 3H, CH_3), 3.35 (s, 3H, CH_3), 3.30–3.09 (m, 4H, H-2, H-5, H-6a, H-3 or H-4), 3.16 (s, 3H, CH_3), 3.00 (dd, 1H, H-6b); ^{13}C NMR (75 MHz, DMSO-d_6): δ = 143.7 (C_{Ar}), 128.2, 127.9 127.0 (CH_{Ar}), 96.5 (C-1), 85.6 (*C*Ph_3),

82.9, 80.9, 79.2, 69.4 (C-2, C-3, C-4, C-5), 62.3 (C-6), 59.9, 59.5, 57.5, 54.2 (4x CH_3); HRMS (ESI), m/z calc. for $C_{29}H_{34}NaO_6$ $[M+Na]^+$: 501.225, found: 501.225; Elemental Analysis for $C_{29}H_{34}O_6$: C: 72.78, H: 7.16, found: C: 72.63, H: 7.23.

Methyl-6-*O*-trityl-2,3,4-*O*-ethyl-β-D-glucopyranoside 5g

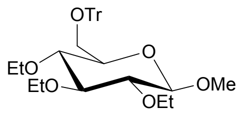

Yield: 2.34 g (82 %); R_f = 0.69 (PE/EA 3:1); $[\alpha]_D^{25}$ = 13.3° (c = 1.0, $CHCl_3$); ^1H NMR (500 MHz, DMSO-d_6): δ = 7.47–7.24 (m, 15H, CH_{Ar}), 4.26 (d, 1H, H-1), 3.81–3.53 (m, 5H, CH_2), 3.50 (s, 3H, CH_3), 3.29–3.24 (m, 4H, CH_2, H-4, H-5, H-6a), 3.14 (t, 1H, $^3J_{H-2,H-3}$ = 8.83 Hz, H-3), 3.01 (dd, 1H, H-2), 2.92 (dd, 1H, H-6b), 1.14–1.09 (m, 6H, 2x CH_3), 0.78 (t, 3H, CH_3); ^{13}C NMR (125 MHz, DMSO-d_6): δ = 143.7 (C_{Ar}), 128.2, 127.8, 127.0 (CH_{Ar}), 103.3 (C-1), 85.5 (*C*Ph_3), 83.9 (C-3), 81.7 (C-2), 77.4, 73.4 (C-4, C-5), 67.8, 67.2, 67.1 (3x CH_2), 62.0 (C-6), 55.7, 5.7, 15.6, 15.4 (4x CH_3); HRMS (ESI), m/z calc. for $C_{32}H_{40}NaO_6$ $[M+Na]^+$: 543.272, found: 543.272; Elemental Analysis for $C_{32}H_{40}O_6$: C: 73.82; H: 7.74, found: C: 73.55; H: 7.81.

General procedure for synthesis of 2,3,4-*O*-methyl-glucopyranosides 5-8d and ethyl ether 5h

5-8c (4.0 mmol) was heated to 70°C in a 70 % acetic acid solution (20 mL) for 1 h. The solvent was then removed by codistillation with toluene. Column chromatography (PE/EA 3:1 to 1:2) led to products **5-8d** and **5h**.

Methyl-2,3,4-*O*-methyl-β-D-glucopyranoside 5d

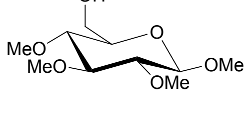

Yield: 756 mg (80 %); m.p. = 90–93°C; R_f = 0.59 (EA); $[\alpha]_D^{25}$ = -24.4° (c = 1.0, MeOH); ^1H NMR (300 MHz, $CDCl_3$): δ = 4.21 (d, 1H, $^3J_{H-1,H-2}$ = 7.74 Hz, H-1), 3.88 (dd, 1H, $^3J_{H-6a,H-6b}$ = 11.90 Hz, H-6a), 3.73 (dd, 1H, H-6b), 3.63 (s, 3H, CH_3), 3.58 (s, 3H, CH_3), 3.56 (s, 3H, CH_3), 3.54 (s, 3H, CH_3), 3.28–3.21 (m, 1H, $^3J_{H-5,H-6b}$ = 4.34 Hz, H-5), 3.21–3.15 (m, 2H, H-3,

H-4), 3.00–2.92 (m, 1H, H-2), 2.02 (s, 1H, OH); ^{13}C NMR (75 MHz, $CDCl_3$): δ = 104.4 (C-1), 86.3, 79.4 (C-3, C-4), 83.8 (C-2), 74.8 (C-5), 62.1 (C-6), 60.8, 60.5, 60.5, 57.2 (4x CH_3); HRMS (ESI), m/z calc. for $C_{10}H_{20}NaO_6$ $[M+Na]^+$: 259.115, found: 259.115; Elemental Analysis for $C_{10}H_{20}O_6$: C: 50.84, H: 8.53, found: C: 51.10, H: 8.65.

Allyl-2,3,4-*O*-methyl-β-D-glucopyranoside 6d

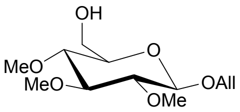

Yield: 944 mg (90 %); m.p. = 48–50°C; R_f = 0.59 (EA); $[\alpha]_D^{24}$ = -30.8° (c = 1.0, $CHCl_3$); ^{1}H NMR (500 MHz, $CDCl_3$): δ = 5.98–5.89 (m, 1H, C*H*=CH_2), 5.33 (dq, 1H, CH=CH_2), 5.21 (dq, 1H, CH=CH_2), 4.38–4.32 (m, 2H, H-1, OCH_2), 4.13 (m, 1H, OCH_2), 3.86 (dq, 1H, $^3J_{H-6a,H-6b}$ = 2.84 Hz, H-6a), 3.74–3.68 (m, 1H, H-6b), 3.63 (s, 3H, CH_3), 3.60 (s, 3H, CH_3), 3.55 (s, 3H, CH_3), 3.25–3.21 (m, 1H, H-5), 3.19–3.15 (m, 2H, H-3, H-4), 3.03–2.99 (m, 1H, H-2), 1.99 (m, 1H, OH); ^{13}C NMR (125 MHz, $CDCl_3$): δ = 133.9 (*C*H=CH_2), 117.3 (CH=*C*H_2), 102.5 (C-1), 86.4, 79.5 (C-3, C-4), 83.9 (C-2), 74.9 (C-5), 70.4 (OCH_2), 62.1 (C-6), 60.8, 60.6, 60.5 (3x CH_3); HRMS (ESI), m/z calc. for $C_{12}H_{22}NaO_6$ $[M+Na]^+$: 285.131, found: 285.131; Elemental Analysis for $C_{12}H_{22}O_6$: C: 54.95, H: 8.45, found: C: 54.96, H: 8.35.

Phenyl-2,3,4-*O*-methyl-β-D-glucopyranoside 7d

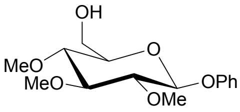

Yield: 937 mg (75 %); m.p. = 103–105°C; R_f = 0.38 (PE/EA 1:1); $[\alpha]_D^{23}$ = -109.1° (c = 1.0, $CHCl_3$); ^{1}H NMR (300 MHz, $CDCl_3$): δ = 7.26–7.20 (m, 2H, CH_{Ar}), 7.01–6.91 (m, 3H, CH_{Ar}), 4.87–4.83 (m, 1H, H-1), 3.82 (dd, 1H, $^3J_{H-6a,H-6b}$ = 11.90 Hz, H-6a), 3.65 (dd, 1H, H-6b), 3.59 (s, 3H, CH_3), 3.59 (s, 3H, CH_3), 3.50 (s, 3H, CH_3), 3.35–3.26 (m, 1H, $^3J_{H-5,H-6a}$ = 9.06 Hz, H-5), 3.23–3.12 (m, 3H, H-2, H-3, H-4); ^{13}C NMR (75 MHz, $CDCl_3$): δ = 157.1 (C_{Ar}), 129.6, 116.5, 122.7 (CH_{Ar}), 101.1 (C-1), 86.2, 83.6, 79.2 (C-2, C-3, C-4), 75.2 (C-5), 62.0 (C-6), 60.9, 60.6, 60.6 (3x CH_3); HRMS (ESI), m/z calc. for $C_{15}H_{22}NaO_6$ $[M+Na]^+$: 321.131, found: 321.131; Elemental Analysis for $C_{15}H_{22}O_6$: C: 60.39, H: 7.43, found: C: 60.18, H: 7.35.

Methyl-2,3,4-*O*-methyl-α-D-glucopyranoside 8d

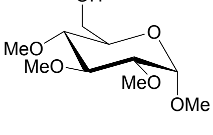

Yield: 822 mg (87 %); m.p. = 29–30°C; R_f = 0.40 (EA); $[\alpha]_D^{23}$ = 148.7° (c = 1.0, $CHCl_3$); ^1H NMR (500 MHz, $CDCl_3$): δ = 4.80 (d, 1H, $^3J_{H-1,H-2}$ = 3.47 Hz, H-1), 3.85–3.80 (m, 1H, H-6a), 3.77–3.69 (m, 1H, H-6b), 3.63 (s, 3H, CH_3), 3.56 (s, 3H, CH_3), 3.52 (s, 3H, CH_3), 3.41 (s, 3H, CH_3), 3.58–3.49 (m, 2H, H-4, H-5), 3.17 (dd, 1H, H-2), 3.16–3.13 (m, 1H, H-3), 1.90 (s, 1H, OH); ^{13}C NMR (125 MHz, $CDCl_3$): δ = 97.5 (C-1), 83.4, 70.6 (C-4, C-5), 81.9 (C-2), 79.7 (C-3), 62.0 (C-6), 60.8, 60.5, 59.0, 55.1 (4x CH_3); HRMS (ESI), m/z calc. for $C_{10}H_{20}NaO_6$ $[M+Na]^+$: 259.115, found: 259.115; Elemental Analysis for $C_{10}H_{20}O_6$: C: 50.84, H: 8.53, found: C: 50.99, H: 8.30.

Methyl-2,3,4-*O*-ethyl-β-D-glucopyranoside 5h

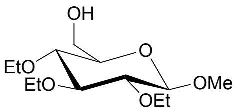

Yield: 813 mg (73 %); m.p. = 83°C; R_f = 0.50 (PE/EA 1:1); $[\alpha]_D^{21}$ = -13.3° (c = 1.0, $CHCl_3$); ^1H NMR (300 MHz, $CDCl_3$): δ = 4.21 (d, 1H, $^3J_{H-1,H-2}$ = 7.74 Hz, H-1), 3.94–3.60 (m, 8H, 3x CH_2, H-6a, H-6b), 3.54 (s, 3H, CH_3), 3.31–3.22 (m, 3H, H-3, H-4, H-5), 3.10–3.00 (m, 1H, H-2), 1.99 (dd, 1H, OH), 1.26–1.16 (m, 9H, 3x CH_3); ^{13}C NMR (75 MHz, $CDCl_3$): δ = 104.6 (C-1), 82.1, (C-2), 84.5, 77.8, 75.0 (C-3, C-4, C-5), 68.9, 68.3, 68.3 (3x CH_2), 62.1 (C-6), 57.3, 15.8, 15.7, 15.7 (4x CH_3); HRMS (ESI), m/z calc. for $C_{13}H_{26}NaO_6$ $[M+Na]^+$: 301.162, found: 301.162; Elemental Analysis for $C_{13}H_{26}O_6$: C: 56.10, H: 9.42, found: C: 56.15, H: 9.25.

General two-step procedure for the introduction of the 6-*O*-triflate group followed by quarternization with pyridine

5-8d or **5h** (4.5 mmol) was dissolved in dry dichloromethane (18 mL) and cooled to 0°C. Pyridine (1.07 mL, 13.3 mmol) was added. Afterwards, Tf_2O (1.5 mL, 9.0 mmol) was added dropwise and the mixture was stirred for 10 min at 0°C. Dichloromethane (20 mL) was added and the mixture was washed with cold water (15 mL), sat. $NaHCO_3$ (2x 15 mL) and cold water again (15 mL). The dried organic phase contains the 6-*O*-triflate intermediates **5-8e** or **5i**. which were directly used in the next step. Pyridine (2.15 mL, 26.6 mmol) was added and the follow-up reaction was performed at the rotary evaporator (40°C, 700 mbar). Further work-up is stated under the respective products **5-8f** or **5j**.

***N*-(Methyl-6-deoxy-2,3,4-*O*-methyl-β-D-glucopyranoside-6-yl)-pyridinium triflate 5f**

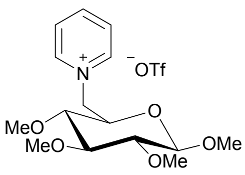

Work-up: The crude product was dissolved in dest. Water (100 mL) and washed with dichloromethane (4x 10 mL) and diethyl ether (2x 10 mL). The aqueous phase was evaporated. The product was then dissolved in dry methanol (10 mL) and a small amount of activated charcoal was added to further purify the product from remaining pyridine. The solvent was evaporated after filtration and the product was dried under high vaccum.

Yield: 1.73 g (86 %); liquid at room temperature; R_f = 0 (EA); $[\alpha]_D^{21}$ = -15.7° (c = 1.0, MeOH); ^{1}H NMR (300 MHz, MeOD): δ = 8.99–8.93 (m, 2H, CH_{Pyr}), 8.65 (t, 1H, CH_{Pyr}), 8.19–8.10 (m, 2H, CH_{Pyr}), 5.07 (dd, 1H, $^3J_{H\text{-}6a,H\text{-}5}$ = 2.64 Hz, H-6a), 4.76 (dd, 1H, H-6b), 4.12 (d, 1H, $^3J_{H\text{-}1,H\text{-}2}$ = 7.74 Hz, H-1), 3.74 (dt, 1H, $^3J_{H\text{-}4,H\text{-}5}$ = 9.44 Hz, H-5), 3.62 (s, 3H, CH_3), 3.61 (s, 3H, CH_3), 3.50 (s, 3H, CH_3), 3.26 (s, 3H, CH_3), 3.26–3.20 (m, 1H, $^3J_{H\text{-}3,H\text{-}4}$ = 9.06 Hz, H-3), 3.06 (t, 1H, H-4), 2.94 (t, 1H, H-2); ^{13}C NMR (75 MHz, MeOD): δ = 147.7, 147.1, 129.3 (CH_{Pyr}), 105.5 (C-1), 87.3 (C-3), 85.0 (C-2), 82.0 (C-4), 74.1 (C-5), 63.7 (C-6), 61.3, 61.0, 60.9, 57.3 (4x CH_3); ^{19}F NMR (282 MHz, MeOD): δ = -78.85; HRMS (ESI), m/z calc. for $C_{15}H_{24}NO_5$ [Cation]$^+$: 298.165, found: 298.165, m/z calc. for CF_3O_3S [Anion]$^-$: 148.953, found: 148.953; Elemental Analysis for $C_{16}H_{24}F_3NO_8S$: C: 42.95, H: 5.41, N: 3.13, found: C: 43.15, H: 5.63, N: 2.87.

_N_-(Allyl-6-deoxy-2,3,4-_O_-methyl-β-D-glucopyranoside-6-yl)-pyridinium triflate 6f

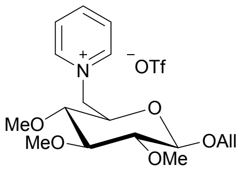

Work-up: The crude product was dissolved in dest. Water (100 mL) and washed with dichloromethane (4x 10 mL) and diethyl ether (2x 10 mL). The aqueous phase was evaporated. The product was then dissolved in dry methanol (10 mL) and a small amount of activated charcoal was added to further purify the product from remaining pyridine. The solvent was evaporated after filtration and the product was dried under high vaccum.

Yield: 1.81 g (85 %); m.p. = 66–70°C; R_f = 0 (EA); $[\alpha]_D^{22}$ = -19.4° (c = 1.1, MeOH); ^{1}H NMR (500 MHz, MeOD): δ = 8.96–8.93 (m, 2H, CH_{Pyr}), 8.68–8.63 (m, 1H, CH_{Pyr}), 8.18-8.13 (m, 1H, CH_{Pyr}), 5.84–5.75 (m, 1H, C*H*=CH$_2$), 5.14 (dq, 1H, CH=C*H*$_2$), 5.09–5.04 (m, 2H, CH=C*H*$_2$, H-6a), 4.75 (dd, 1H, $^3J_{H-5,H-6b}$ = 9.18 Hz, H-6b), 4.26 (d, 1H, $^3J_{H-1,H-2}$ = 7.65 Hz, H-1), 4.02 (m, 1H, OCH_2), 3.87 (m, 1H, OCH_2), 3.74 (dt, 1H, $^3J_{H-4,H-5}$ = 9.56 Hz, H-5), 3.62 (s, 3H, CH_3), 3.61 (s, 3H, CH_3), 3.53 (s, 3H, CH_3), 3.25 (t, 1H, H-3), 3.07 (t, 1H, H-4), 2.99 (dd, 1H, H-2); ^{13}C NMR (125 MHz, MeOD): δ = 147.7, 147.1 (CH_{Pyr}), 135.4 (*C*H=CH$_2$), 129.3 (CH_{Pyr}), 117.4 (CH=*C*H$_2$), 103.8 (C-1), 87.3 (C-3), 85.1 (C-2), 82.0 (C-4), 74.2 (C-5), 71.5 (OCH_2), 63.7 (C-6), 61.2, 61.0, 61.0 (3x CH_3); ^{19}F NMR (282 MHz, MeOD): δ = -80.08; HRMS (ESI), m/z calc. for $C_{17}H_{26}NO_5$ [Cation]$^+$: 324.181, found: 324.180, m/z calc. for CF_3O_3S [Anion]$^-$: 148.953, found: 148.953; Elemental Analysis for $C_{18}H_{26}F_3NO_8S$: C: 45.66, H: 5.54, N: 2.96, S: 6.77, found: C: 45.61, H: 5.52, N: 2.75, S: 6.91.

***N*-(Phenyl-6-deoxy-2,3,4-*O*-methyl-β-D-glucopyranoside-6-yl)-pyridinium triflate 7f**

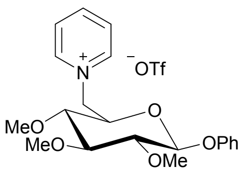

Work-up: The crude product was dissolved in dichloromethane (100 mL) and washed with cold water (2x 10 mL). The organic phase was evaporated and the product was crystalized from ethanol.

Yield: 1.10 g (48 %); m.p. = 164–172°C; R_f = 0 (EA); $[\alpha]_D^{22}$ = 41.5° (c = 0.7, acetone); ^{1}H NMR (300 MHz, MeOD): δ = 8.86 (dd, 2H, CH_{Pyr}), 8.64-8.57 (m, 1H, CH_{Pyr}), 8.01 (dd, 2H, CH_{Pyr}), 7.16-7.07 (m, 2H, CH_{Ar}), 7.01–6.94 (m, 1H, CH_{Ar}), 6.61–6,55 (m, 2H, CH_{Ar}), 5.10 (dd, 1H, $^3J_{H-6a,H-6b}$ = 13.41 Hz, H-6a), 4.89 (d, 1H, $^3J_{H-1,H-2}$ = 7.55 Hz, H-1), 4.74 (dd, 1H, $^3J_{H-5,H-6b}$ = 9.82 Hz, H-6b), 3.94 (dt, 1H, H-5), 3.66 (s, 6H, 2x CH_3), 3.63 (s, 3H, CH_3), 3.37 (q, 1H, $^3J_{H-3,H-4}$ = 8.69 Hz, H-3), 3.29–3.25 (m, 1H, H-2), 3.21 (q, 1H, H-4); ^{13}C NMR (75 MHz, MeOD): δ = 158.0 (C_{Ar}), 147.6, 147.0, 129.2 (CH_{Pyr}), 129.3, 123.9, 117.7 (CH_{Ar}), 101.4 (C-1), 87.4 (C-3), 83.9 (C-2), 82.1 (C-4), 74.4 (C-5), 63.6 (C-6), 61.4, 61.2, 61.1 (3x CH_3); ^{19}F NMR (282 MHz, MeOD): δ = -80.08; HRMS (ESI), m/z calc. for $C_{20}H_{26}NO_5$ [Cation]$^+$: 360.181, found: 360.180, m/z calc. for CF_3O_3S [Anion]$^-$: 148.953, found: 148.953; Elemental Analysis for $C_{21}H_{26}F_3NO_8S$: C: 49.50, H: 5.14, N: 2.75, found: C: 49.70, H: 5.35, N: 2.58.

***N*-(Methyl-6-deoxy-2,3,4-*O*-methyl-α-D-glucopyranoside-6-yl)-pyridinium triflate 8f**

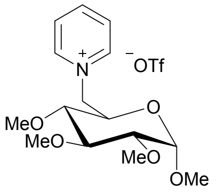

Work-up: The crude product was dissolved in dest. Water (100 mL) and washed with dichloromethane (4x 10 mL) and diethyl ether (2x 10 mL). The aqueous phase was evaporated. The product was then dissolved in dry methanol (10 mL) and a small amount of activated charcoal was added to further purify the product from remaining pyridine. The solvent was evaporated after filtration and the product was dried under high vaccum.

Yield: 1.69 g (84 %); liquid at room temperature; R_f = 0 (EA); $[\alpha]_D^{23}$ = 75.1° (c = 1.0, MeOH); ^{1}H NMR (500 MHz, MeOD): δ = 9.01–8.96 (m, 2H, CH_{Pyr}), 8.68–8.63 (m, 1H, CH_{Pyr}), 8.17–8.12 (m, 2H, CH_{Pyr}), 5.05 (dd, 1H, $^3J_{H-6a,H-5}$ = 2.68 Hz, H-6a), 4.82 (d, 1H, $^3J_{H-1,H-2}$ = 3.44 Hz, H-1), 4.77 (dd, 1H, H-6b), 3.90 (dt, 1H, H-5), 3.63 (s, 3H, CH_3), 3.58 (s, 3H, CH_3), 3.45 (s, 3H, CH_3), 3.48–3.42 (m, 1H, H-3), 3.24 (dd, 1H, H-2), 3.05 (dd, 1H, H-4), 2.94 (s, 3H, CH_3); ^{13}C NMR (125 MHz, MeOD): δ = 147.7 (CH_{Pyr}), 147.1 (CH_{Pyr}), 129.4 (CH_{Pyr}), 98.9 (C-1), 84.6 (C-3), 82.8 (C-2), 82.0 (C-4), 71.0 (C-5), 63.6 (C-6), 61.2, 61.1, 59.1, 55.6 (4x CH_3); ^{19}F NMR (282 MHz, MeOD): δ = -80.06; HRMS (ESI), m/z calc. for $C_{15}H_{24}NO_5$ [Cation]$^+$: 298.165, found: 298.165, m/z calc. for CF_3O_3S [Anion]$^-$: 148.953, found: 148.953; Elemental Analysis for $C_{16}H_{24}F_3NO_8S$: C: 42.95, H: 5.41, N: 3.13, found: C: 42.87, H: 5.67, N: 3.10.

***N*-(Methyl-6-deoxy-2,3,4-*O*-ethyl-β-D-glucopyranoside-6-yl)-pyridinium triflate 5j**

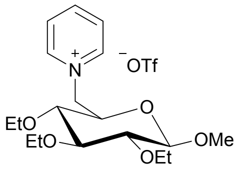

Work-up: The crude product was dissolved in dest. Water (100 mL) and washed with dichloromethane (4x 10 mL) and diethyl ether (2x 10 mL). The aqueous phase was evaporated. The product was then dissolved in dry methanol (10 mL) and a small amount of activated charcoal was added to further purify the product from remaining pyridine. The solvent was evaporated after filtration and the product was dried under high vaccum.

Yield: 1.81 g (82 %); m.p. = 114–116°C; R_f = 0 (EA); $[\alpha]_D^{23}$ = -18.0° (c = 1.0, MeOH); ^{1}H NMR (300 MHz, MeOD): δ = 8.97 (dd, 2H, CH_{Pyr}), 8.68–8.61 (m, 1H, CH_{Pyr}), 8.15 (dd, 2H, CH_{Pyr}), 5.07 (dd, 1H, $^3J_{H-6a,H-6b}$ = 13.41 Hz, H-6a), 4.75 (dd, 1H, H-6b), 4.09 (d, 1H, $^3J_{H-1,H-2}$ = 7.74 Hz, H-1), 4.02–3.56 (m, 7H, 3x CH_2, H-5), 3.34–3.28 (m, 1H, H-3), 3.22 (s, 3H, CH_3), 3.17 (t, 1H, H-4), 3.03 (dd, 1H, H-2), 1.27 (t, 3H, CH_3), 1.21 (t, 3H, CH_3), 1.14 (t, 3H, CH_3); ^{13}C NMR

(75 MHz, MeOD): δ = 147.7, 147.1, 129.2 (CH_{Pyr}), 105.6 (C-1), 85.6 (C-3), 83.4 (C-2), 80.7 (C-4), 74.3 (C-5), 70.1, 69.7, 69.4 (3x CH_2), 63.9 (C-6), 57.38, 16.3, 16.2, 16.1 (4x CH_3); ^{19}F NMR (282 MHz, MeOD): δ = -80.06; HRMS (ESI), m/z calc. for $C_{18}H_{30}NO_5$ $[Cation]^+$: 340.212, found: 340.212, m/z calc. for CF_3O_3S $[Anion]^-$: 148.953, found: 148.953; Elemental Analysis for $C_{19}H_{30}F_3NO_8S$: C: 46.62, H: 6.18, N: 2.86, found: : 46.51, H: 6.09, N: 2.99.

Procedure for the introduction of the 6-*O*-mesylate group on 5d

5d (827 mg, 3.5 mmol) was dissolved in dry pyridine (20 mL), mesyl chloride (0.39 mL, 5.0 mmol) was added and the reaction was stirred at room temperature for 12 h. Dichloromethane (100 mL) was added and the mixture was washed with cold water (20 mL), 15 % aqueous $NaHSO_4$ solution (3x 20 mL) and cold water again (20 mL). Column chromatography (PE/EA 2:1) led to product **5k**.

Methyl-2,3,4-*O*-methyl-6-*O*-mesyl-β-D-glucopyranoside 5k

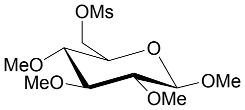

Yield: 1.05 g (95 %); R_f = 0.60 (EA); $[\alpha]_D^{23}$ = -17.7° (c = 1.0, $CHCl_3$); ^1H NMR (500 MHz, $CDCl_3$): δ = 4.48 (dd, 1H, $^3J_{H\text{-}6a,H\text{-}5}$ = 2.21 Hz, H-6a), 4.38 (dd, 1H, $^3J_{H\text{-}6a,H\text{-}6b}$ = 11.35 Hz, H-6b), 4.18 (d, 1H, $^3J_{H\text{-}1,H\text{-}2}$ = 7.88 Hz, H-1), 3.62 (s, 3H, CH_3), 3.56 (s, 3H, CH_3), 3.56 (s, 3H, CH_3), 3.52 (s, 3H, CH_3), 3.40 (ddd, 1H, $^3J_{H\text{-}4,H\text{-}5}$ = 9.77 Hz, H-5), 3.18 (t, 1H, $^3J_{H\text{-}3,H\text{-}4}$ = 8.83 Hz, H-3), 3.10 (t, 1H, H-4), 3.06 (s, 3H, SO_2CH_3), 2.96 (t, 1H, H-2); ^{13}C NMR (125 MHz, $CDCl_3$): δ = 104.2 (C-1), 86.3 (C-3), 83.6 (C-2), 78.7 (C-4), 72.7 (C-5), 68.5 (C-6), 60.8, 60.5, 60.4, 57.1 (4x CH_3), 37.7 (SO_2CH_3); HRMS (ESI), m/z calc. for $C_{11}H_{22}NaO_8S$ $[M+Na]^+$: 337.093, found: 337.093; Elemental Analysis for $C_{11}H_{22}O_8S$: C: 42.03, H: 7.05, S: 10.20, found: C: 42.21, H: 7.08, S: 10.08.

Procedure for the quarternization of the 6-*O*-mesylate 5k

5k (944 mg, 3.0 mmol) was dissolved in dry pyridine (5 mL) and heated at 125°C for 3 h. The crude product was dissolved in dest. water (100 mL) and washed with dichloromethane (4x 10 mL) and diethyl ether (2x 10 mL). The aqueous phase was evaporated. The product was then dissolved in dry methanol (10 mL) and a small amount of activated charcoal was added to further purify the product from remaining pyridine. The solvent was evaporated after filtration and the product **5l** was dried under high vaccum.

N-(Methyl-6-deoxy-2,3,4-O-methyl-β-D-glucopyranoside-6-yl)-pyridinium mesylate 5l

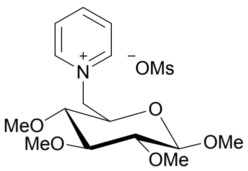

Yield: 1.11 g (94 %); m.p. = 60–63°C; R_f = 0 (EA); $[\alpha]_D^{27}$ = -23.2° (c = 1.0, MeOH); ^{1}H NMR (300 MHz, MeOD): δ = 9.03–8.98 (d, 2H, CH_{Pyr}), 8.73–8.65 (m, 1H, CH_{Pyr}), 8.23–8.15 (m, 2H, CH_{Pyr}), 5.11 (dd, 1H, $^3J_{H-6a,H-5}$ = 2.83 Hz, H-6a), 4.81 (dd, 1H, $^3J_{H-6b,H-5}$ = 9.25 Hz, H-6b), 4.16 (d, 1H, $^3J_{H-1,H-2}$ = 7.74 Hz, H-1), 3.78 (dt, 1H, $^3J_{H-4,H-5}$ = 9.44 Hz, H-5), 3.66 (s, 3H, CH_3), 3.64 (s, 3H, CH_3), 3.54 (s, 3H, CH_3), 3.29 (s, 3H, CH_3), 3.32-3.24 (m, 1H, H-3), 3.10 (dd, 1H, H-4), 2.98 (dd, 1H, H-2), 2.73 (s, 3H, SO_2CH_3); ^{13}C NMR (75 MHz, MeOD): δ = 147.7, 147.1, 129.3 (CH_{Pyr}), 105.5 (C-1), 87.4 (C-3), 85.1 (C-2), 82.0 (C-4), 74.2 (C-5), 63.7 (C-6), 61.3, 61.0, 60.9, 57.3 (4x CH_3), 39.6 (SO_2CH_3); HRMS (ESI), m/z calc. for $C_{15}H_{24}NO_5$ [Cation]$^+$: 298.165, found: 298.165, m/z calc. for CH_3O_3S [Anion]$^-$: 94.981, found: 94.981; Elemental Analysis for $C_{16}H_{27}NO_8S$: C: 48.84, H: 6.92, N: 3.56, S: 8.15, found: C: 48.65, H: 7.14, N: 3.49, S: 8.07.

Procedure for the introduction of the 6-O-tosylate group on 5d

5d (827 mg, 3.5 mmol) was dissolved in dry pyridine (25 mL), tosyl chloride (950 mg, 5.0 mmol) was added and the reaction was stirred at room temperature for 12 h. Dichloromethane (100 mL) was added and the mixture was washed with cold water (20 mL), 15 % aqueous $NaHSO_4$ solution (3x 20 mL) and cold water again (20 mL). Column chromatography (PE/EA 2:1) led to product **5m**.

Methyl-2,3,4-O-methyl-6-O-tosyl-β-D-glucopyranoside 5m

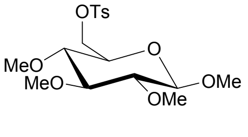

Yield: 1.23 g (90 %); m.p. = 65–68°C; R_f = 0.61 (PE/EA 1:1); $[\alpha]_D^{22}$ = -12.4° (c = 1.0, $CHCl_3$); ^{1}H NMR (300 MHz, $CDCl_3$): δ = 7.85–7.78 (m, 2H, CH_{Ar}), 7.38–7.31 (m, 2H, CH_{Ar}), 4.27 (dd, 1H, $^3J_{H-6a,H-6b}$ = 10.58 Hz, H-6a), 4.19 (dd, 1H, $^3J_{H-5,H-6b}$ = 5.10 Hz, H-6b), 4.09 (d, 1H, $^3J_{H-1,H-2}$ = 7.74 Hz, H-1), 3.60 (s, 3H, CH_3), 3.54 (s, 3H, CH_3), 3.48 (s, 3H, CH_3), 3.53 (s, 3H, CH_3),

3.35 (dt, 1H, $^3J_{H-4,H-5}$ = 9.63 Hz, H-5), 3.14 (t, 1H, $^3J_{H-2,H-3}$ = 8.69 Hz, H-3), 3.03 (dd, 1H, $^3J_{H-3,H-4}$ = 8.88 Hz, H-4), 2.97 (dd, 1H, H-2), 2.45 (s, 3H, Tos-CH_3); ^{13}C NMR (63 MHz, $CDCl_3$): δ = 144.8, 133.0 (C_{Ar}), 129.8, 128.0 (CH_{Ar}), 103.9 (C-1), 86.3 (C-3), 83.4 (C-2), 78.8 (C-4), 72.5 (C-5), 68.7 (C-6), 60.7, 60.4, 60.4, 56.8 (4x CH_3), 21.6 (Tos-CH_3); HRMS (ESI), m/z calc. for $C_{17}H_{26}NaO_8S$ $[M+Na]^+$: 413.124, found: 413.124; Elemental Analysis for $C_{17}H_{26}O_8S$: C: 52.29, H: 6.71, S: 8.21, found: C: 52.34, H: 6.70, S: 8.18.

Procedure for the quarternization of the 6-*O*-tosylate 5m

5m (1.17 g, 3.0 mmol) was dissolved in dry pyridine (5 mL) and heated at 125°C for 3 h. The crude product was dissolved in dest. water (100 mL) and washed with dichloromethane (4x 10 mL) and diethyl ether (2x 10 mL). The aqueous phase was evaporated. The product was then dissolved in dry methanol (10 mL) and a small amount of activated charcoal was added to further purify the product from remaining pyridine. The solvent was evaporated after filtration and the product **5n** was dried under high vaccum. The product was crystalized from dichloromethane.

N-(Methyl-6-deoxy-2,3,4-*O*-methyl-β-D-glucopyranoside-6-yl)-pyridinium tosylate 5n

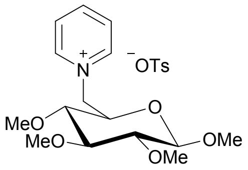

Yield: 1.06 g (75 %); m.p. = 135–137°C; R_f = 0 (EA); $[\alpha]_D^{21}$ = -19.8° (c = 1.0, MeOH); ^1H NMR (500 MHz, MeOD): δ = 8.98–8.91 (m, 2H, CH_{Pyr}), 8.65 (t, 1H, CH_{Pyr}), 8.19–8.08 (m, 2H, CH_{Pyr}), 7.74–7.66 (m, 2H, CH_{Ar}), 7.26–7.20 (m, 2H, CH_{Ar}), 5.06 (dd, 1H, $^3J_{H-6a,H-5}$ = 2.83 Hz, H-6a), 4.76 (dd, 1H, $^3J_{H-6b,H-5}$ = 9.25 Hz, H-6b), 4.11 (d, 1H, $^3J_{H-1,H-2}$ = 7.74 Hz, H-1), 3.73 (dt, 1H, $^3J_{H-4,H-5}$ = 9.44 Hz, H-5), 3.62 (s, 3H, CH_3), 3.60 (s, 3H, CH_3), 3.50 (s, 3H, CH_3), 3.25 (s, 3H, CH_3), 3.28–3.19 (m, 1H, $^3J_{H-3,H-4}$ = 8.83 Hz, H-3), 3.05 (dd, 1H, H-4), 2.93 (dd, 1H, H-2), 2.37 (s, 3H, Tos-CH_3); ^{13}C NMR (75 MHz, MeOD): δ = 147.7, 147.1 (CH_{Pyr}), 143.9, 147.8 (C_{Ar}), 129.9 (CH_{Ar}), 129.2 (CH_{Pyr}), 127.1 (CH_{Ar}), 105.4 (C-1), 87.3 (C-3), 85.0 (C- 2), 82.0 (C-4), 74.1 (C-5), 63.7 (C-6), 61.2, 61.0, 60.9, 57.3 (4x CH_3), 21.5 (Tos-CH_3); HRMS (ESI), m/z calc. for $C_{15}H_{24}NO_5$ $[Cation]^+$: 298.165, found: 298.165, m/z calc. for $C_7H_7O_3S$ $[Anion]^-$: 171.012, found: 171.012; Elemental Analysis for $C_{22}H_{31}NO_8S$: C: 56.27, H: 6.65, N: 2.98, found: C: 56.07, H: 6.67, N: 2.83.

NMR Spectra of all final pentose based ionic products

N-(2,3-*O*-Methyl-1,5-deoxy-D-ribofuranoside-5-yl)-pyridinium triflate 1i

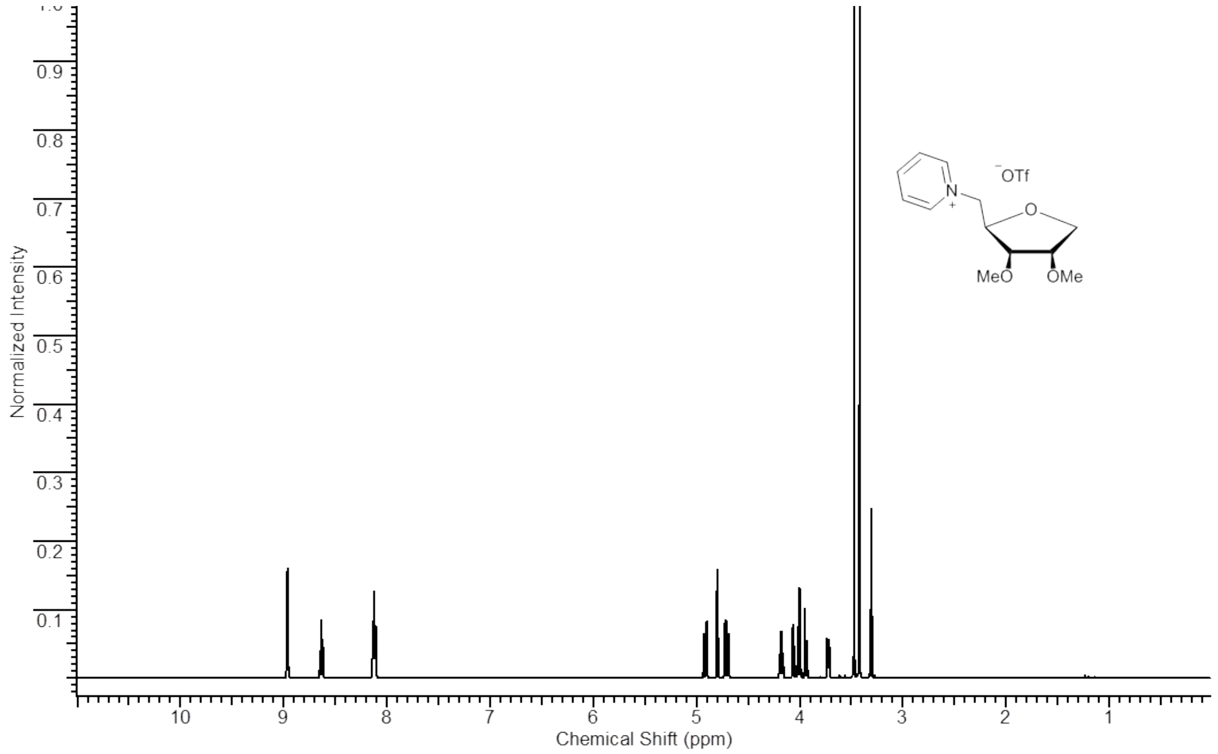

Figure 1: ^{1}H spectrum of compound **1i** (500 MHz, MeOD).

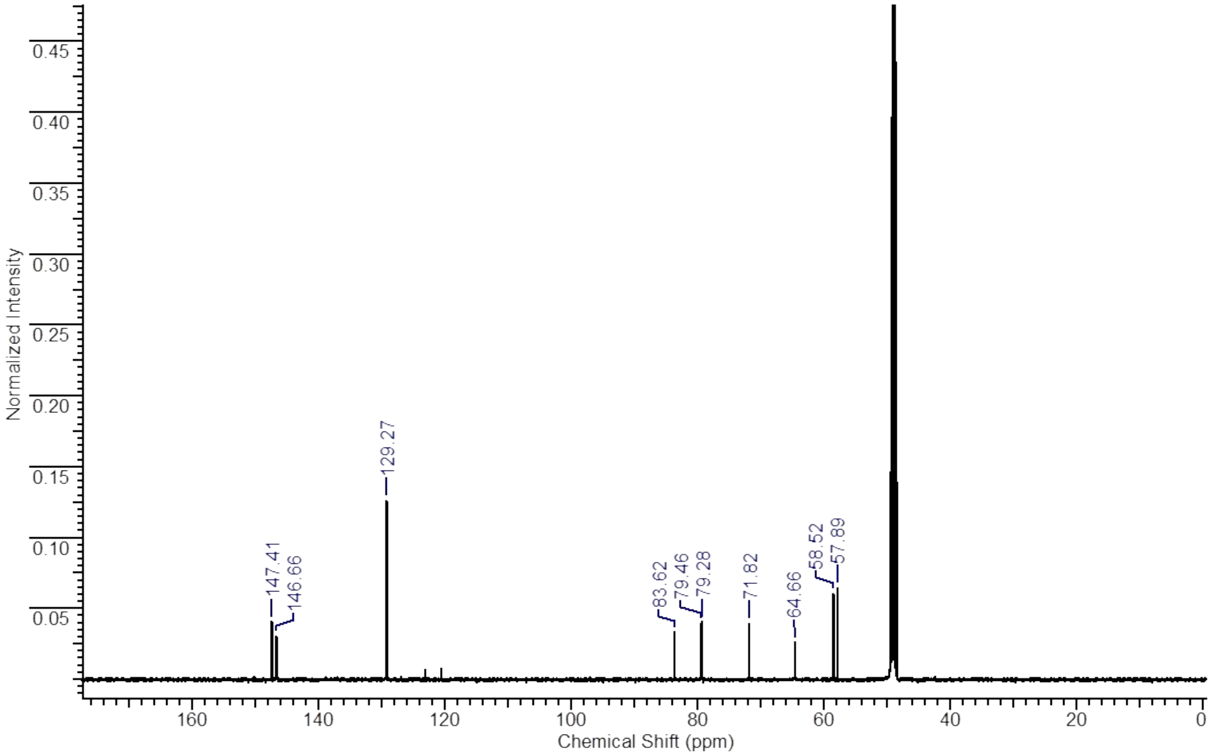

Figure 2: ^{13}C spectrum of compound **1i** (125 MHz, MeOD).

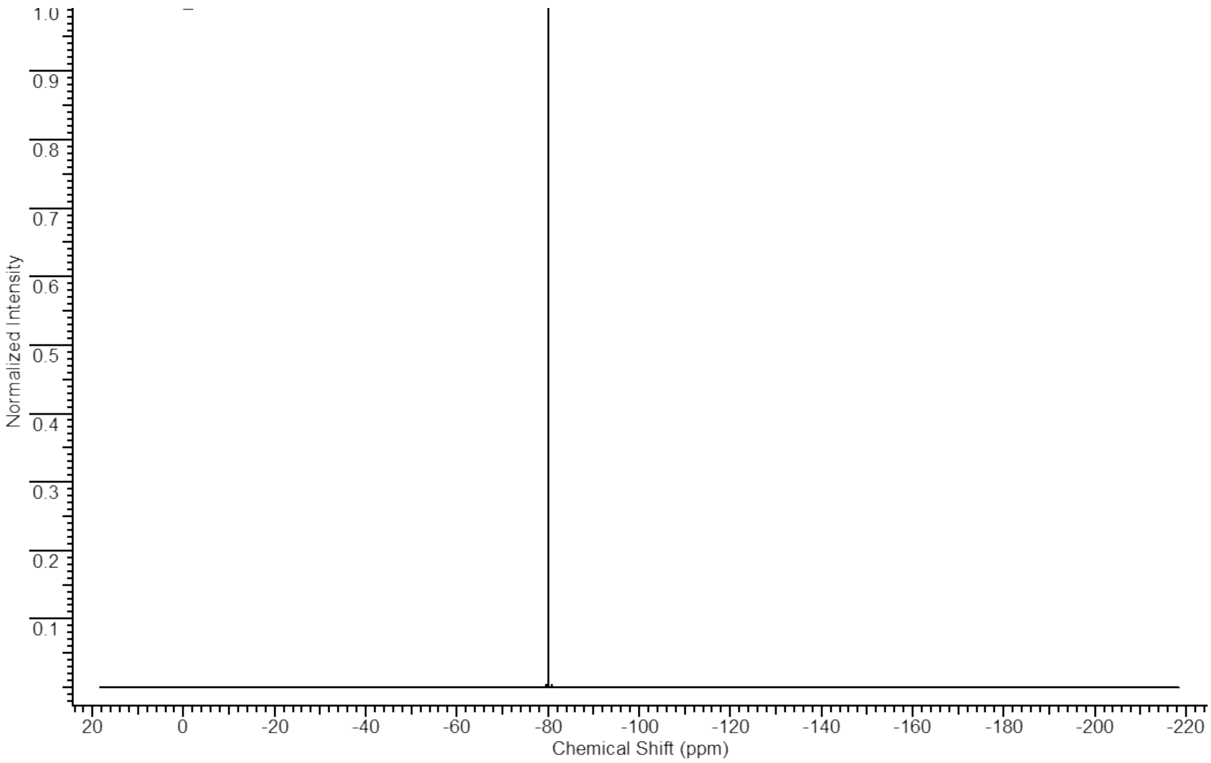

Figure 3: ^{19}F spectrum of compound **1i** (282 MHz, MeOD).

***N*-(2,3-*O*-Methyl-1,5-deoxy-D-lyxofuranoside-5-yl)-pyridinium triflate 2i**

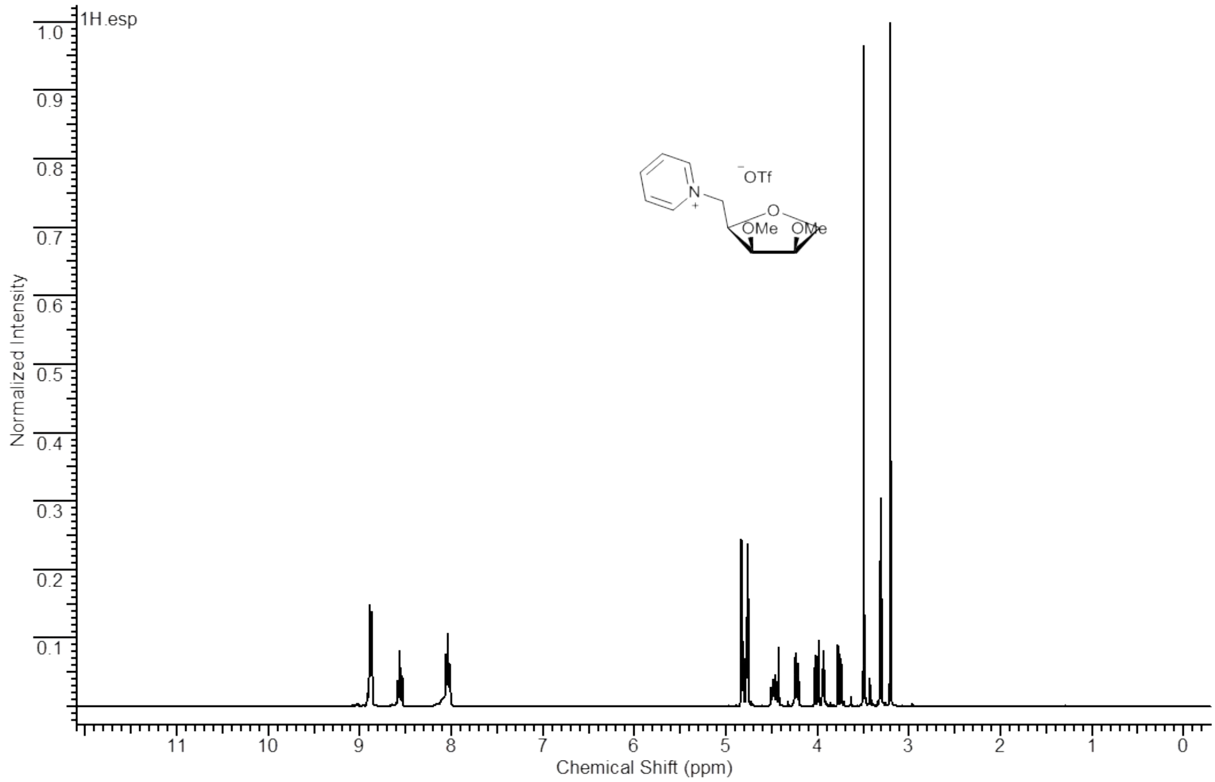

Figure 4: ^{1}H spectrum of compound **2i** (300 MHz, MeOD).

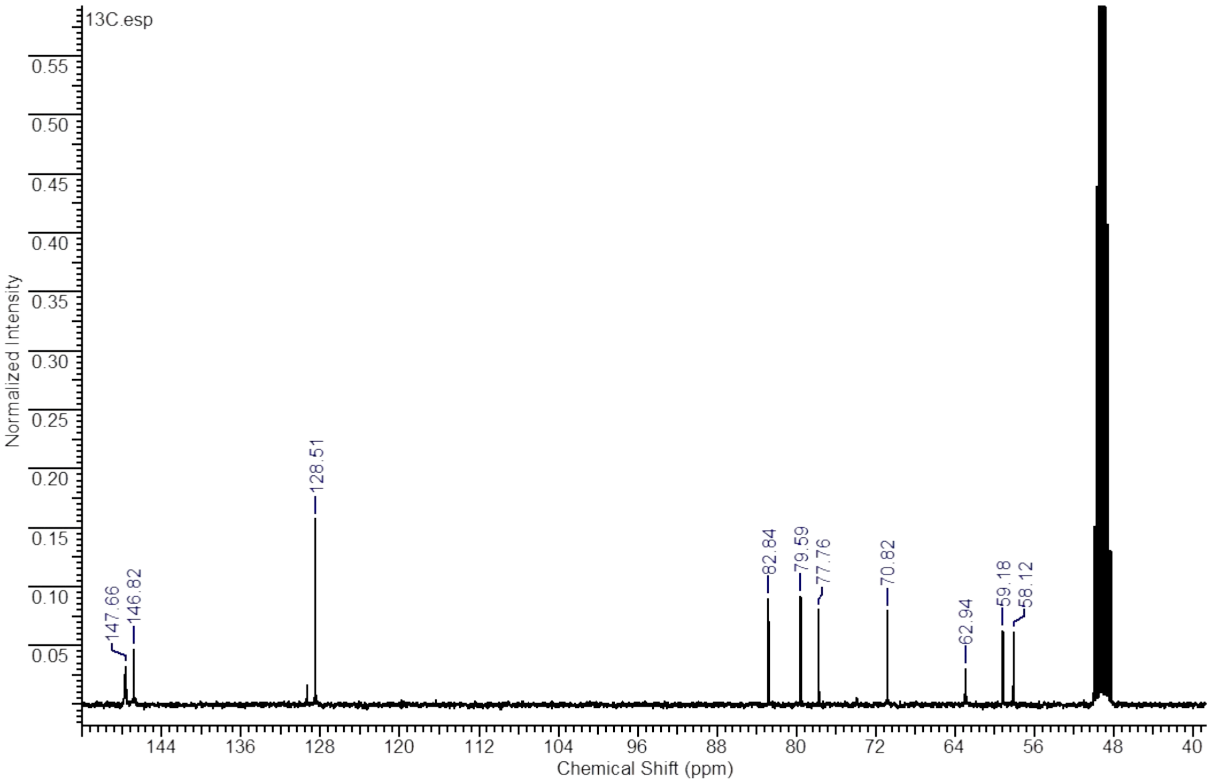

Figure 5: ^{13}C spectrum of compound **2i** (75 MHz, MeOD).

Figure 6: ^{19}F spectrum of compound **2i** (282 MHz, MeOD).

N-(2,3-O-Methyl-1,5-deoxy-D-xylofuranoside-5-yl)-pyridinium triflate 3i

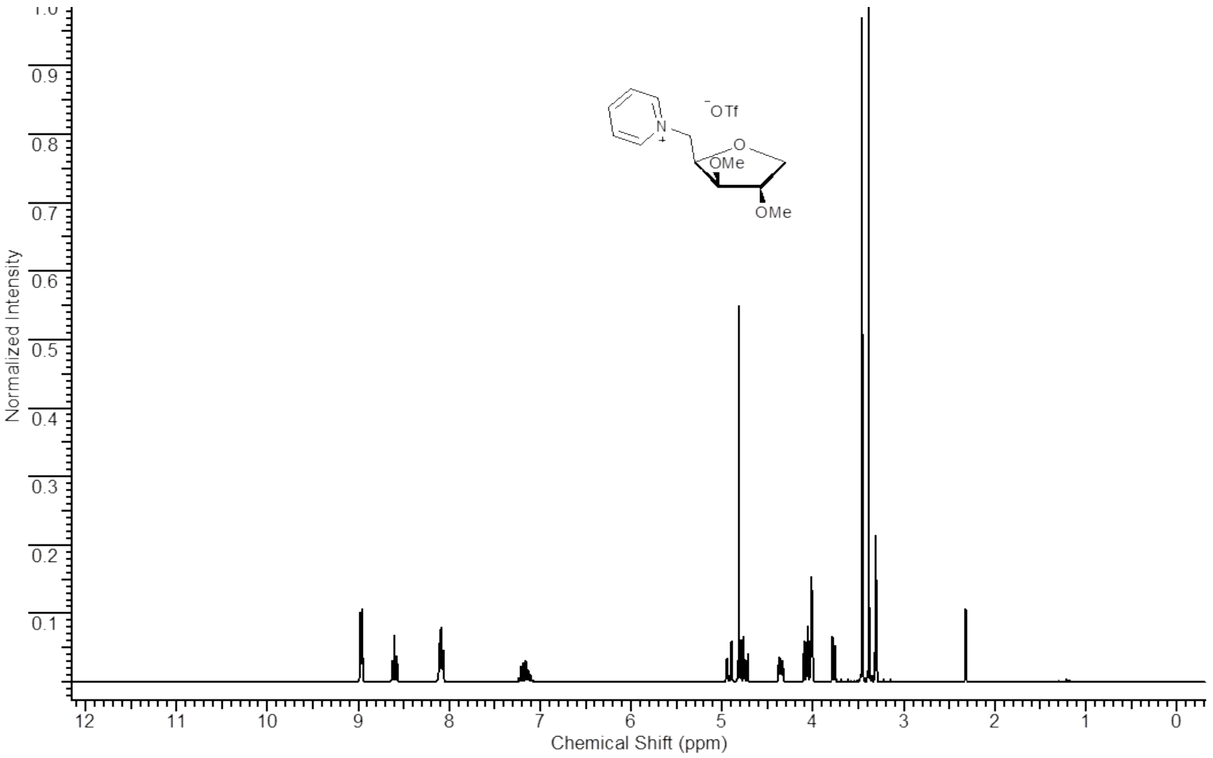

Figure 7: ^{1}H spectrum of compound **3i** (300 MHz, MeOD).

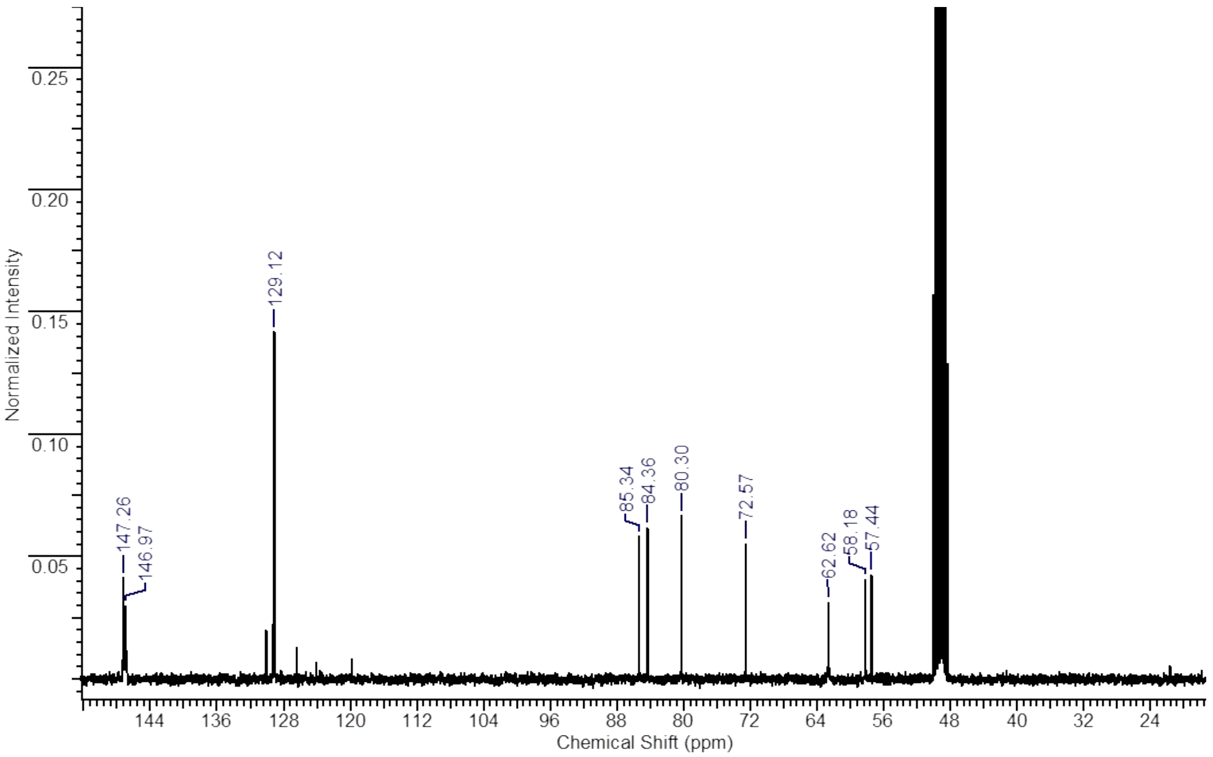

Figure 8: ^{13}C spectrum of compound **3i** (75 MHz, MeOD).

Figure 9: ^{19}F spectrum of compound **3i** (282 MHz, MeOD).

***N*-(2,3-*O*-Methyl-1,5-deoxy-L-arabinofuranoside-5-yl)-pyridinium triflate 4i**

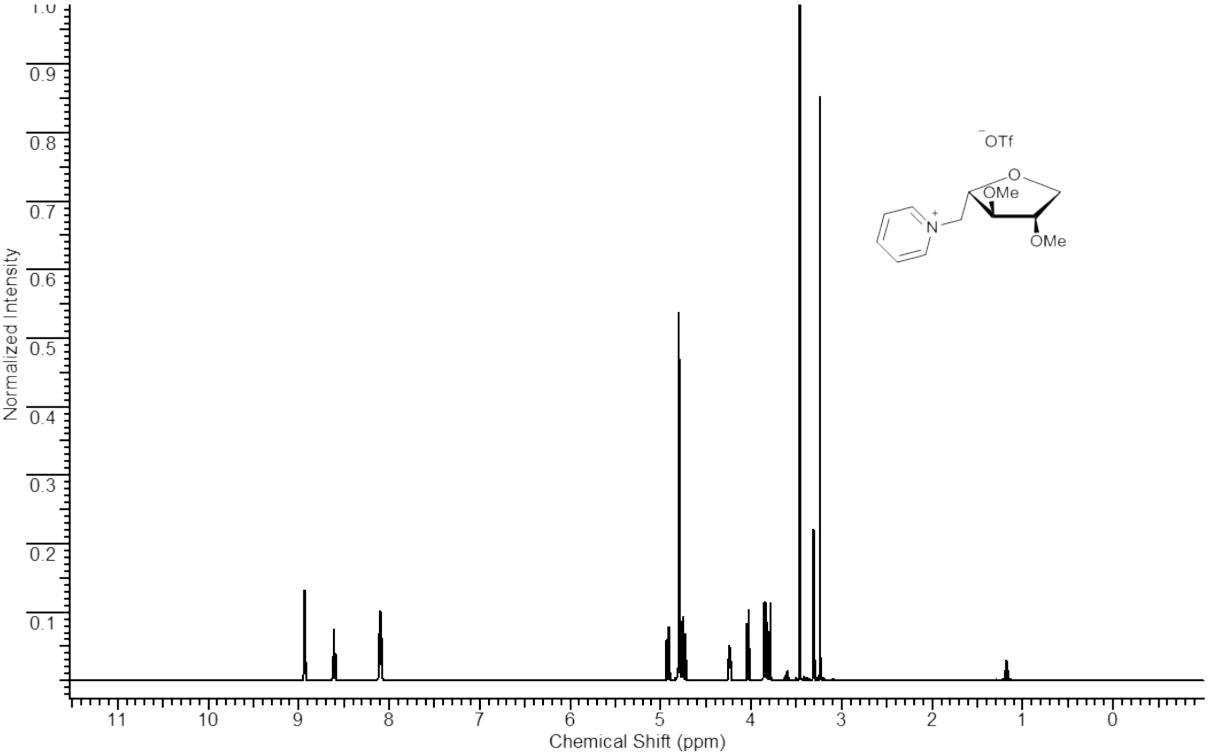

Figure 10: ^{1}H spectrum of compound **4i** (500 MHz, MeOD).

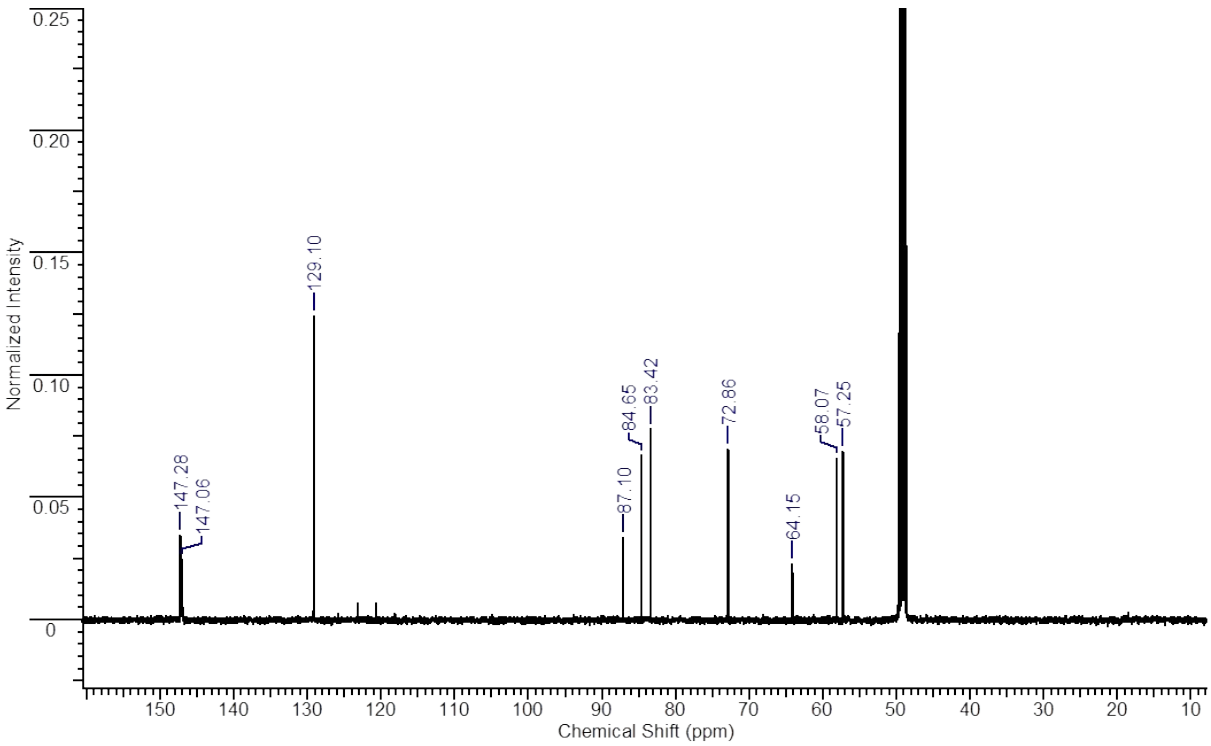

Figure 11: ^{13}C spectrum of compound **4i** (125 MHz, MeOD).

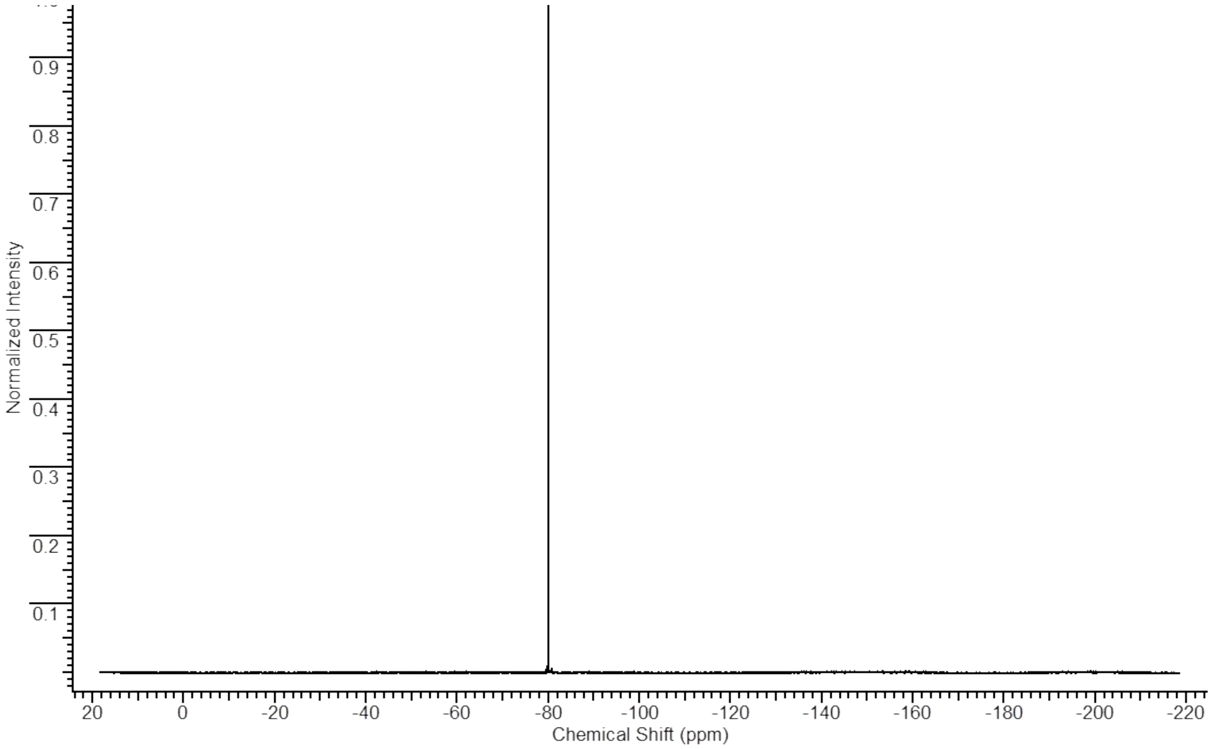

Figure 12: ^{19}F spectrum of compound **4i** (282 MHz, MeOD).

N-(2,3-*O*-Ethyl-1,5-deoxy-D-ribofuranoside-5-yl)-pyridinium triflate 1o

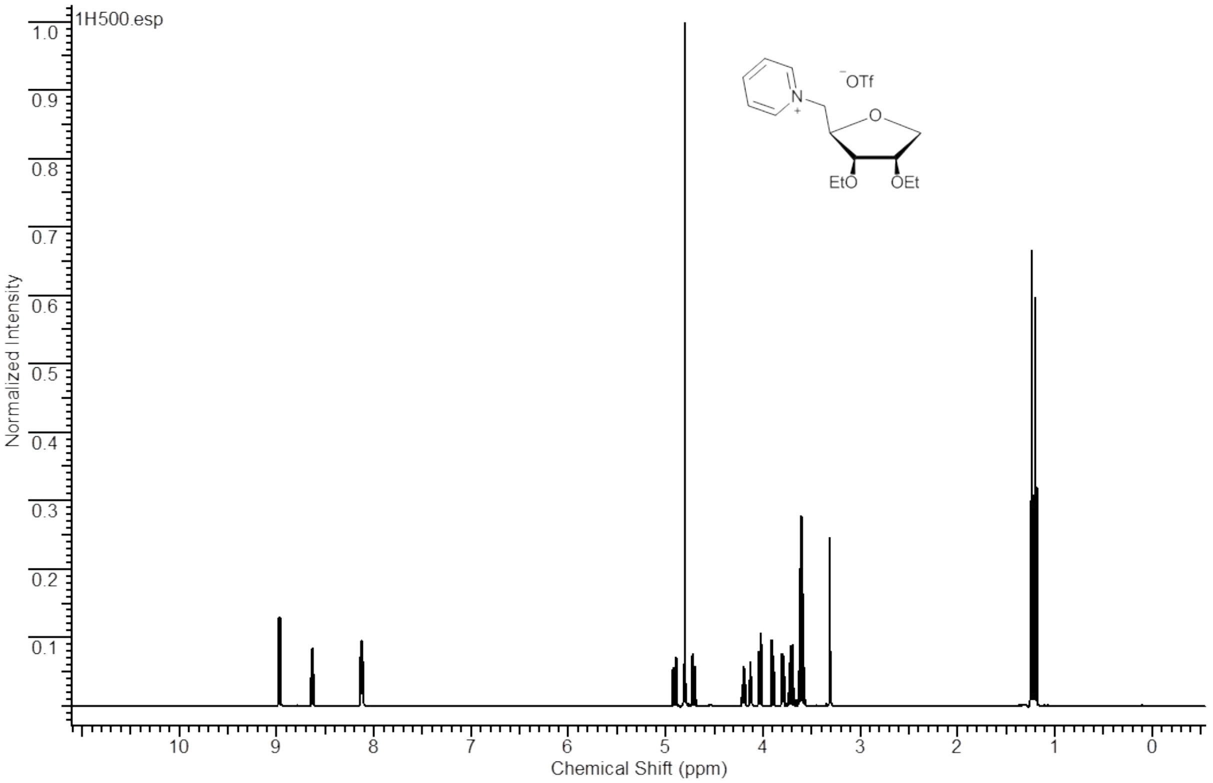

Figure 13: ^{1}H spectrum of compound **1o** (500 MHz, MeOD).

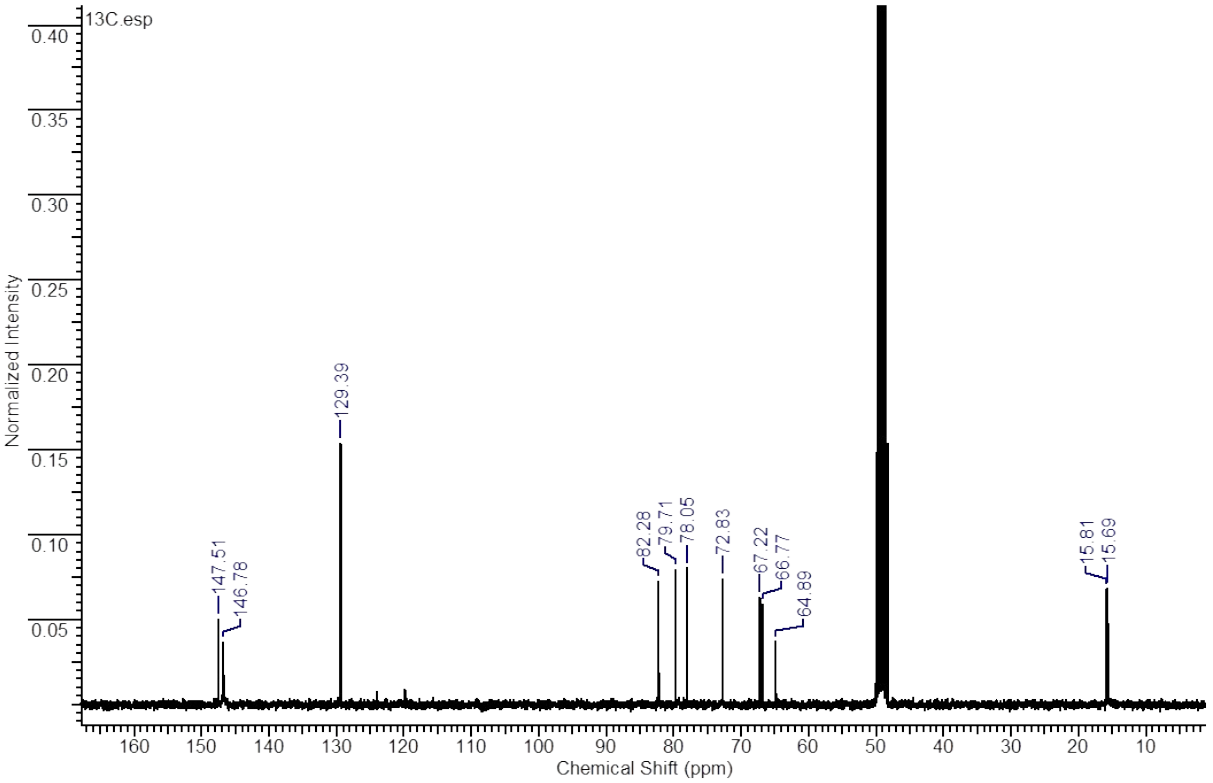

Figure 14: ^{13}C spectrum of compound **1o** (75 MHz, MeOD).

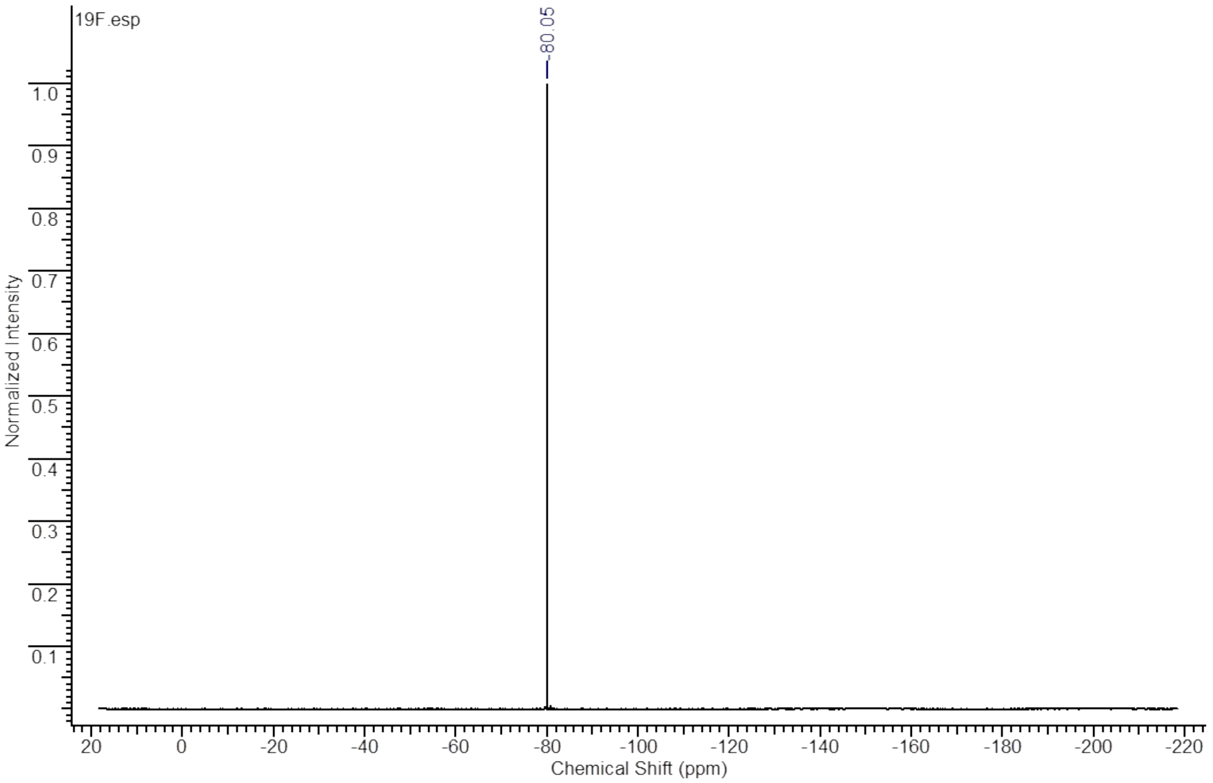

Figure 15: ^{19}F spectrum of compound **1o** (282 MHz, MeOD).

***N*-(2,3-*O*-Allyl-1,5-deoxy-D-ribofuranoside-5-yl)-pyridinium triflate 1s**

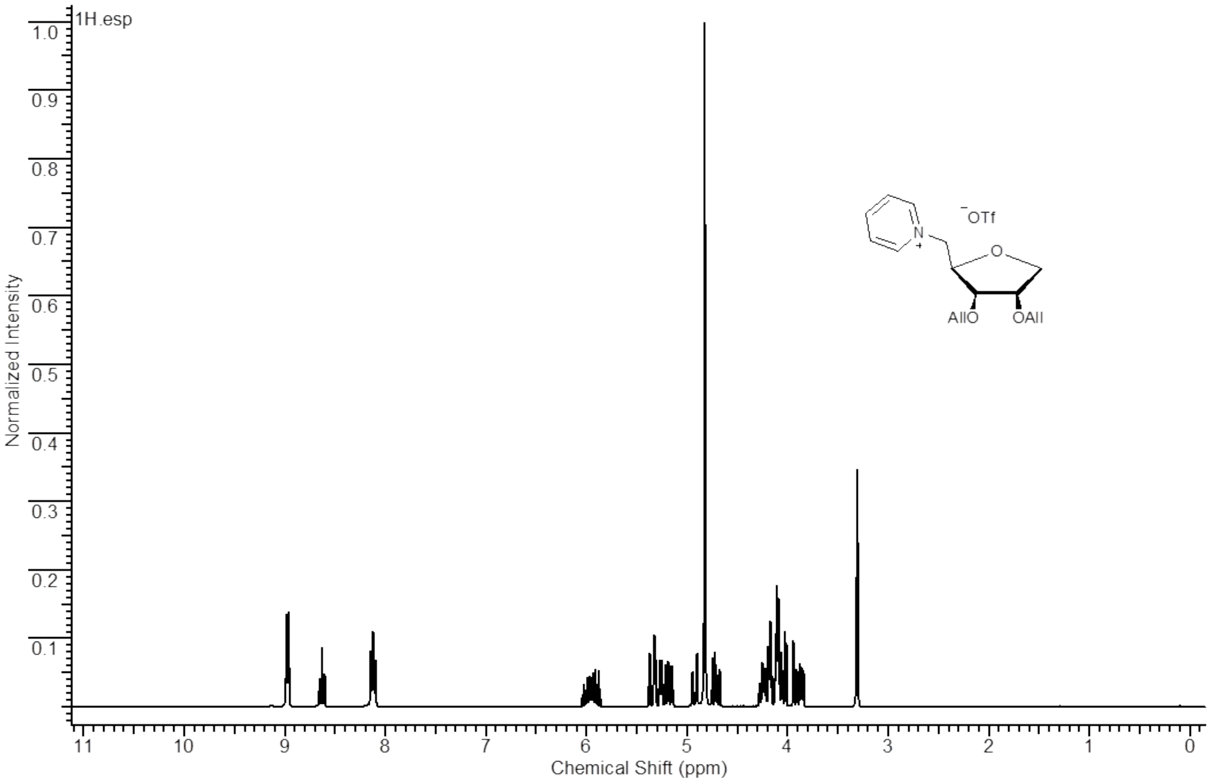

Figure 16: ^{1}H spectrum of compound **1s** (300 MHz, MeOD).

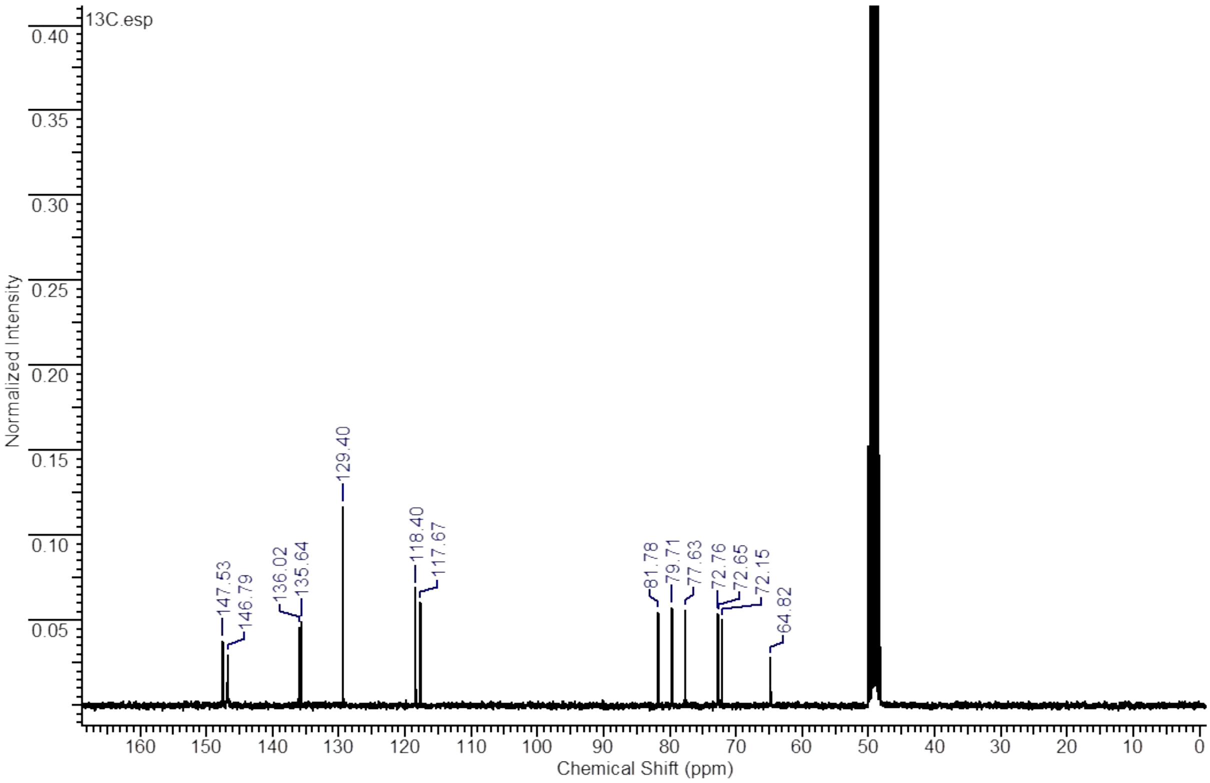

Figure 17: ^{13}C spectrum of compound **1s** (75 MHz, MeOD).

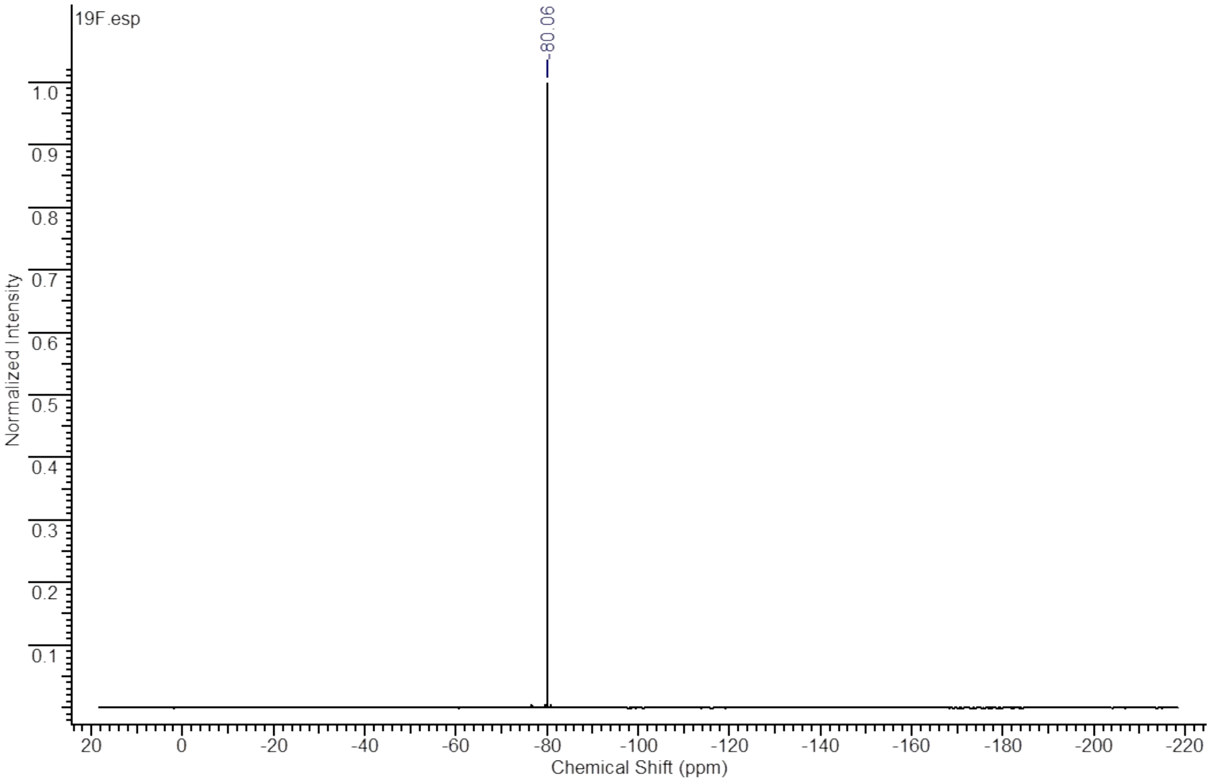

Figure 18: ^{19}F spectrum of compound **1s** (282 MHz, MeOD).

N-(2,3-*O*-Propyl-1,5-deoxy-D-ribofuranoside-5-yl)-pyridinium triflate 1v

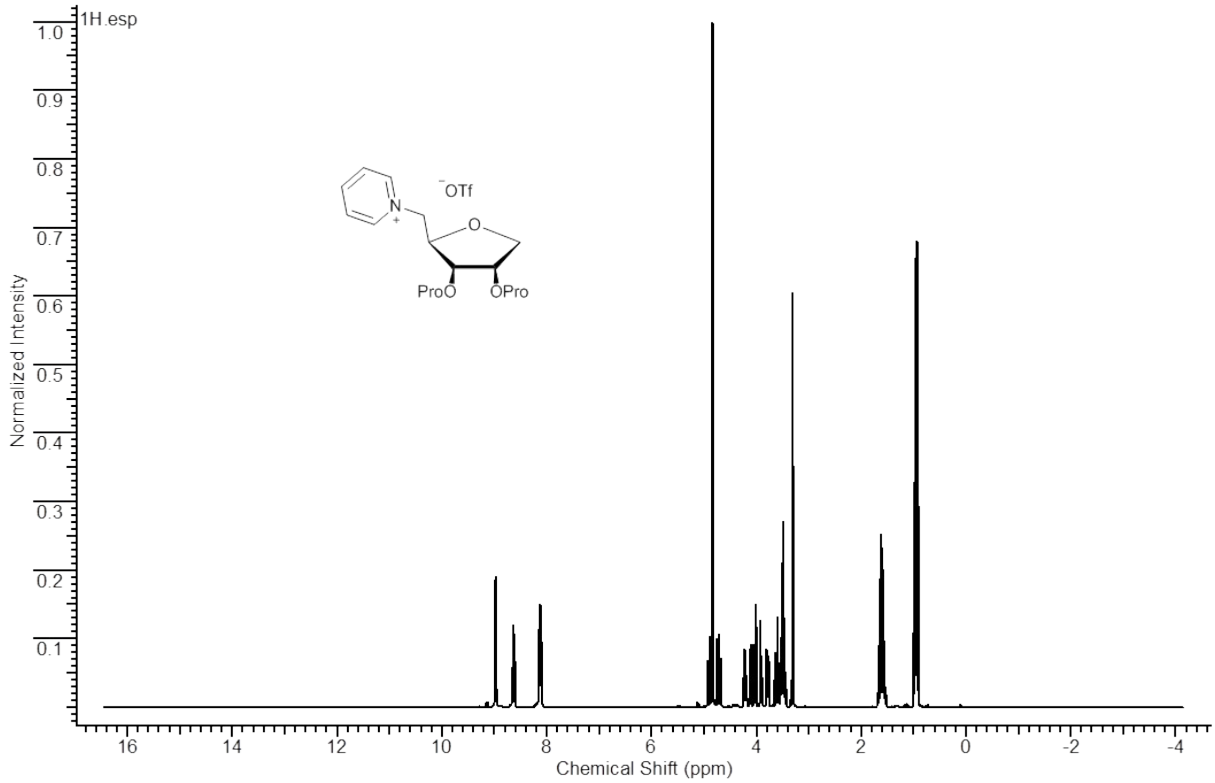

Figure 19: ^{1}H spectrum of compound **1v** (300 MHz, MeOD).

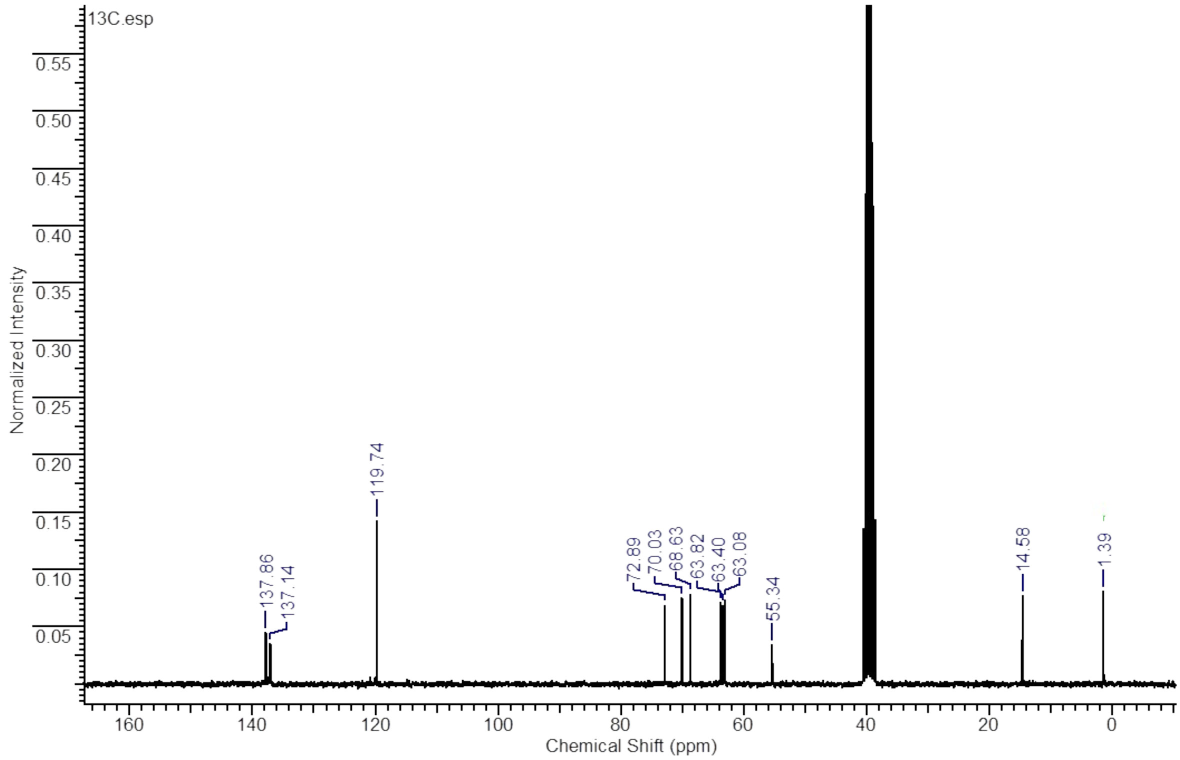

Figure 20: ^{13}C spectrum of compound **1v** (75 MHz, MeOD).

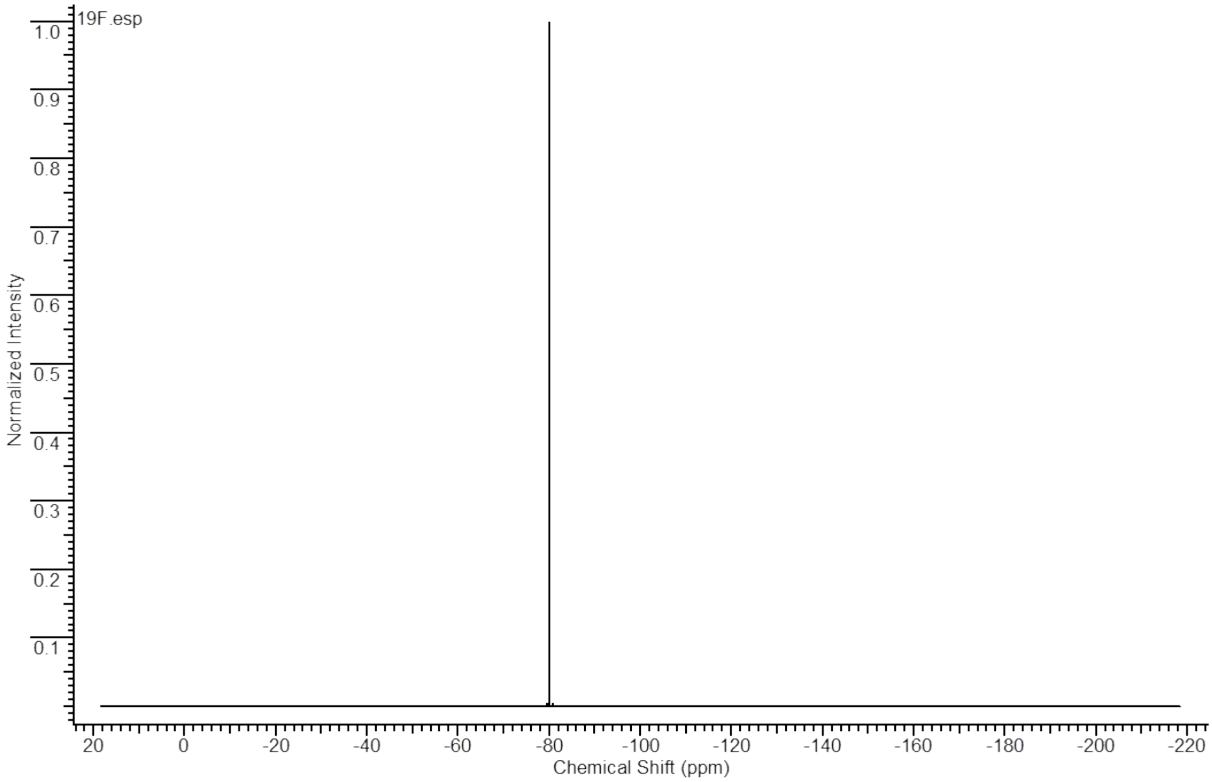

Figure 21: ^{19}F spectrum of compound **1v** (282 MHz, MeOD).

N-(1,5-deoxy-D-ribofuranoside-5-yl)-pyridinium triflate 1k

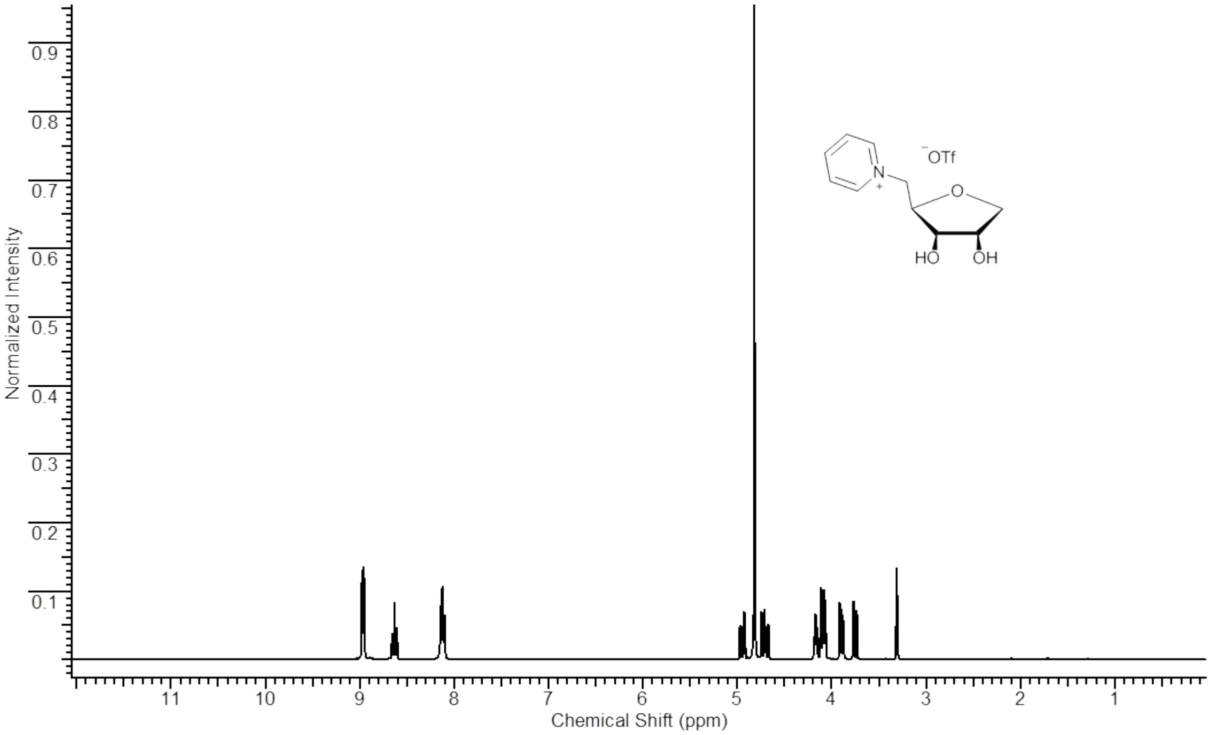

Figure 22: ^{1}H spectrum of compound **1k** (300 MHz, MeOD).

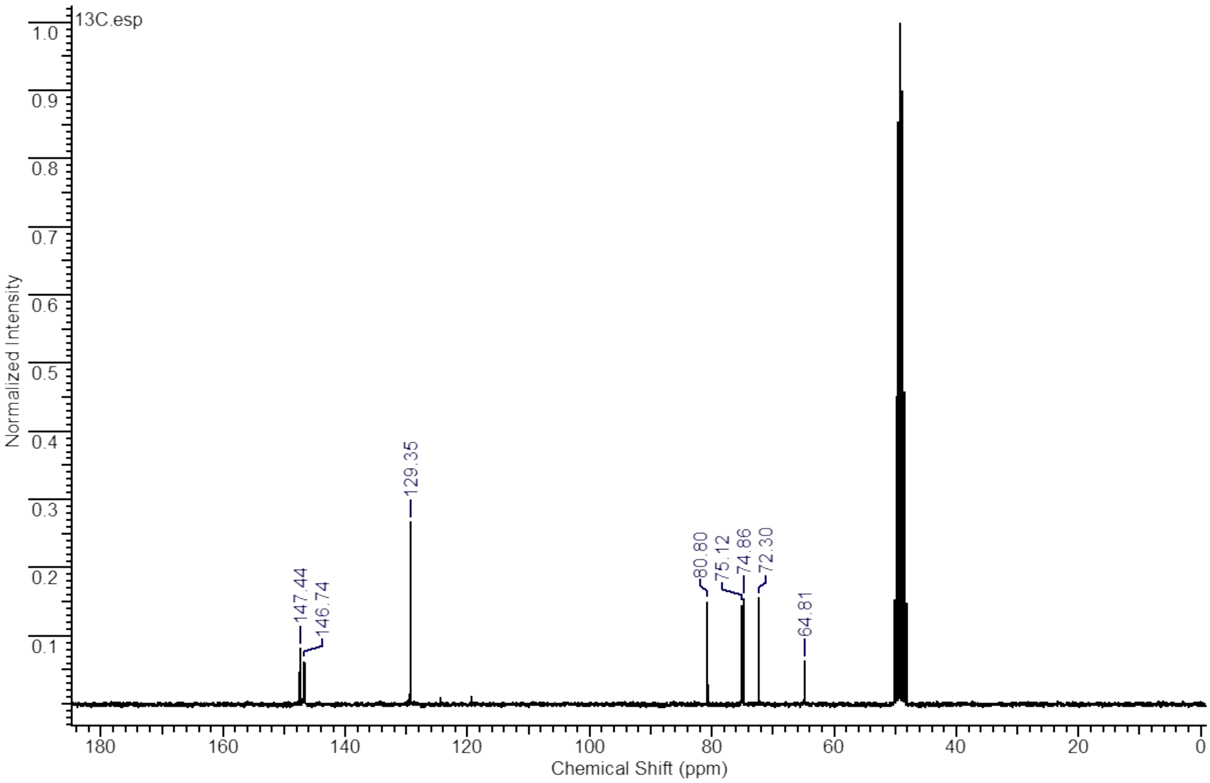

Figure 23: ^{13}C spectrum of compound **1k** (63 MHz, MeOD).

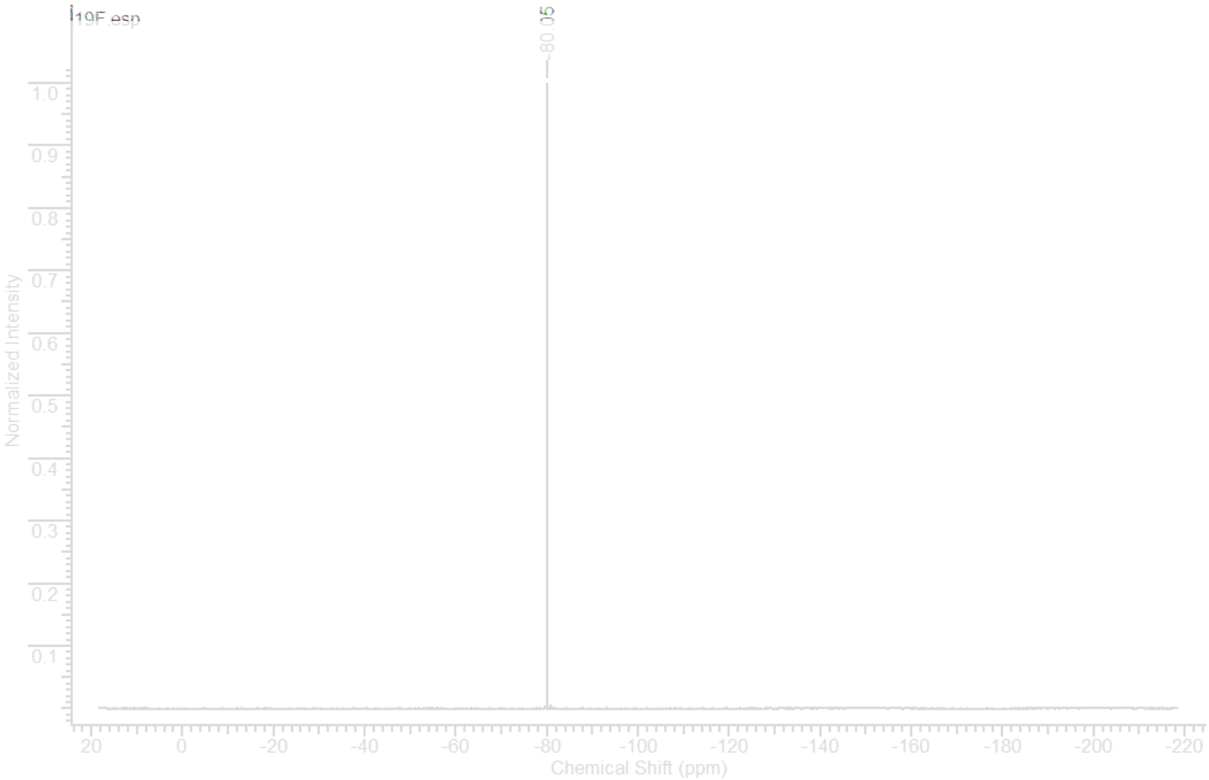

Figure 24: ^{19}F spectrum of compound **1k** (282 MHz, MeOD).

N-(2,3-*O*-Isopropylidene-1,5-deoxy-D-ribofuranoside-5-yl)-pyridinium mesylate 1x

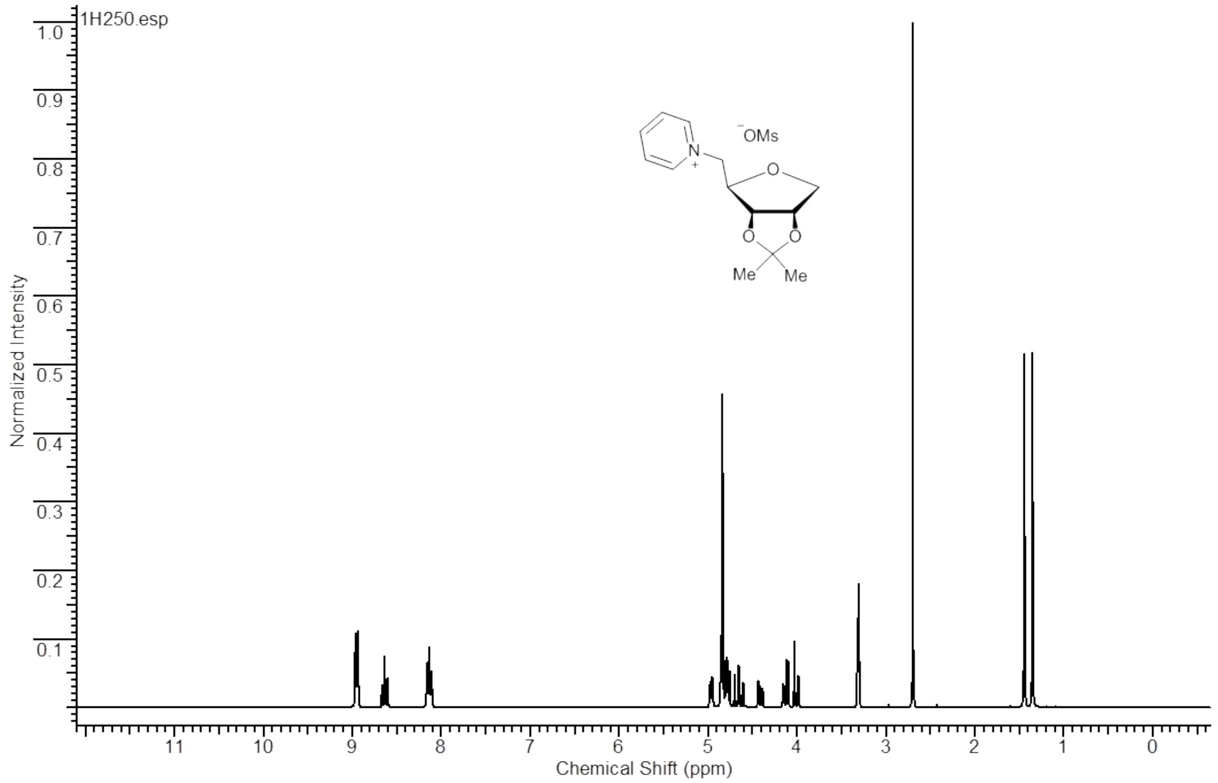

Figure 25: ^{1}H spectrum of compound **1x** (250 MHz, MeOD).

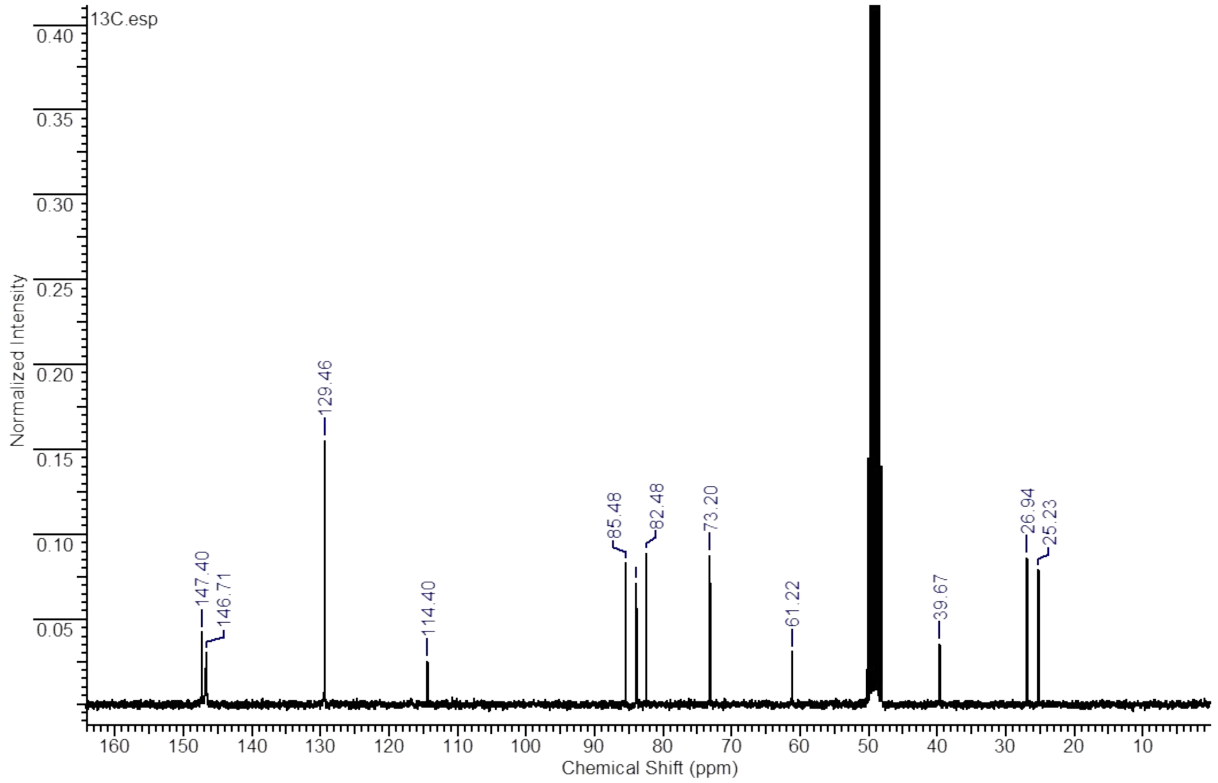

Figure 26: ^{13}C spectrum of compound **1x** (75 MHz, MeOD).

NMR Spectra of all final glucose based ionic products

N-(Methyl-6-deoxy-2,3,4-*O*-methyl-β-D-glucopyranoside-6-yl)-pyridinium triflate 5f

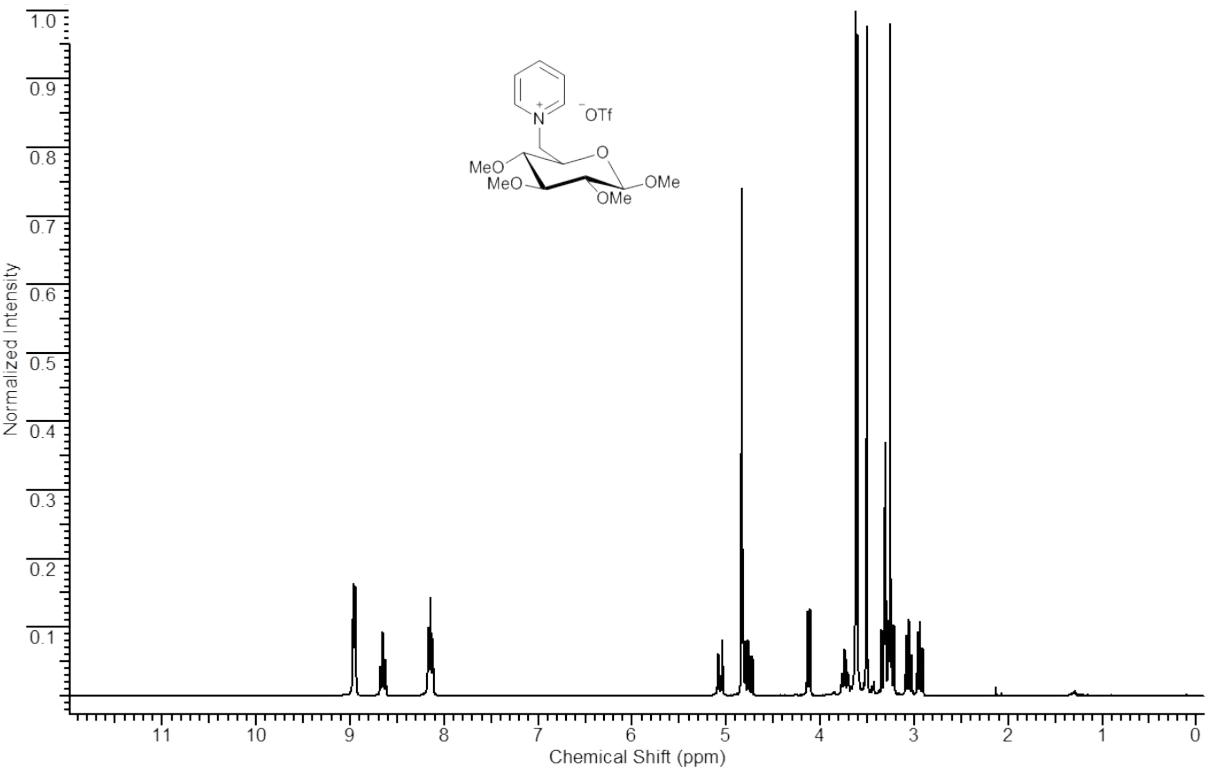

Figure 27: ^{1}H NMR spectrum of compound **5f** (300 MHz, MeOD).

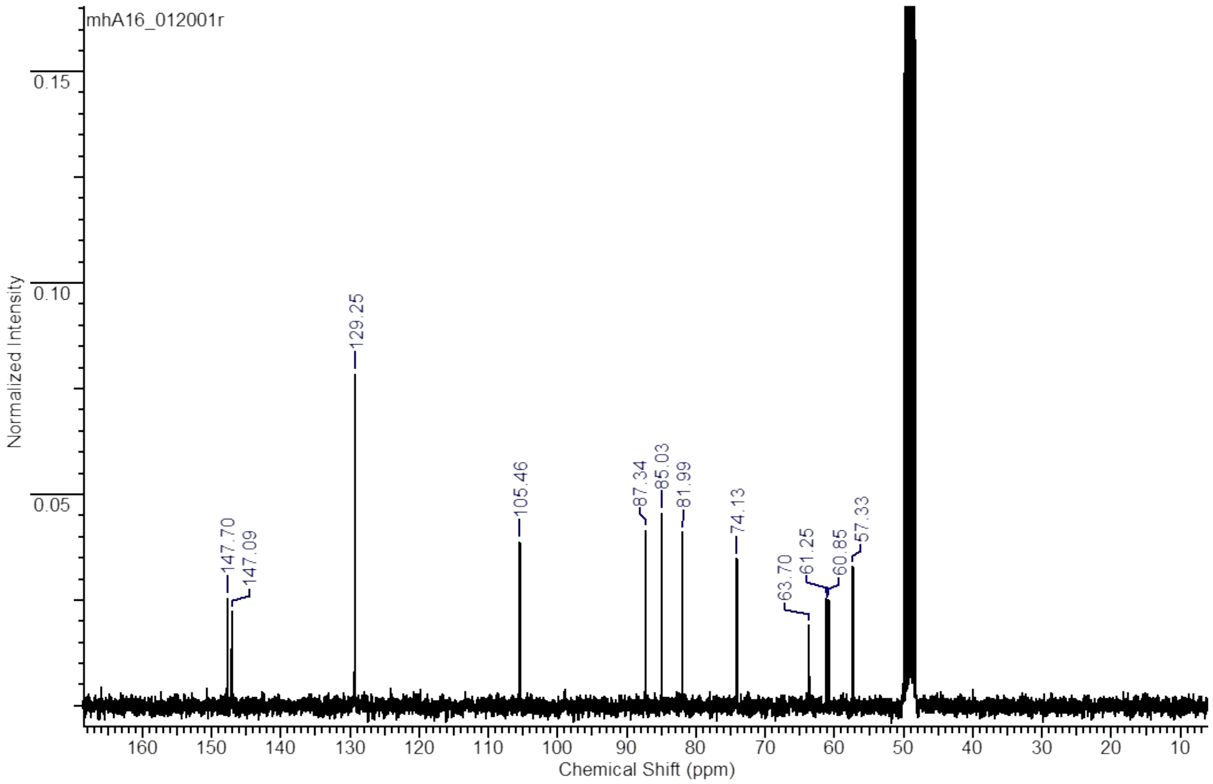

Figure 28: ^{13}C spectrum of compound **5f** (75 MHz, MeOD).

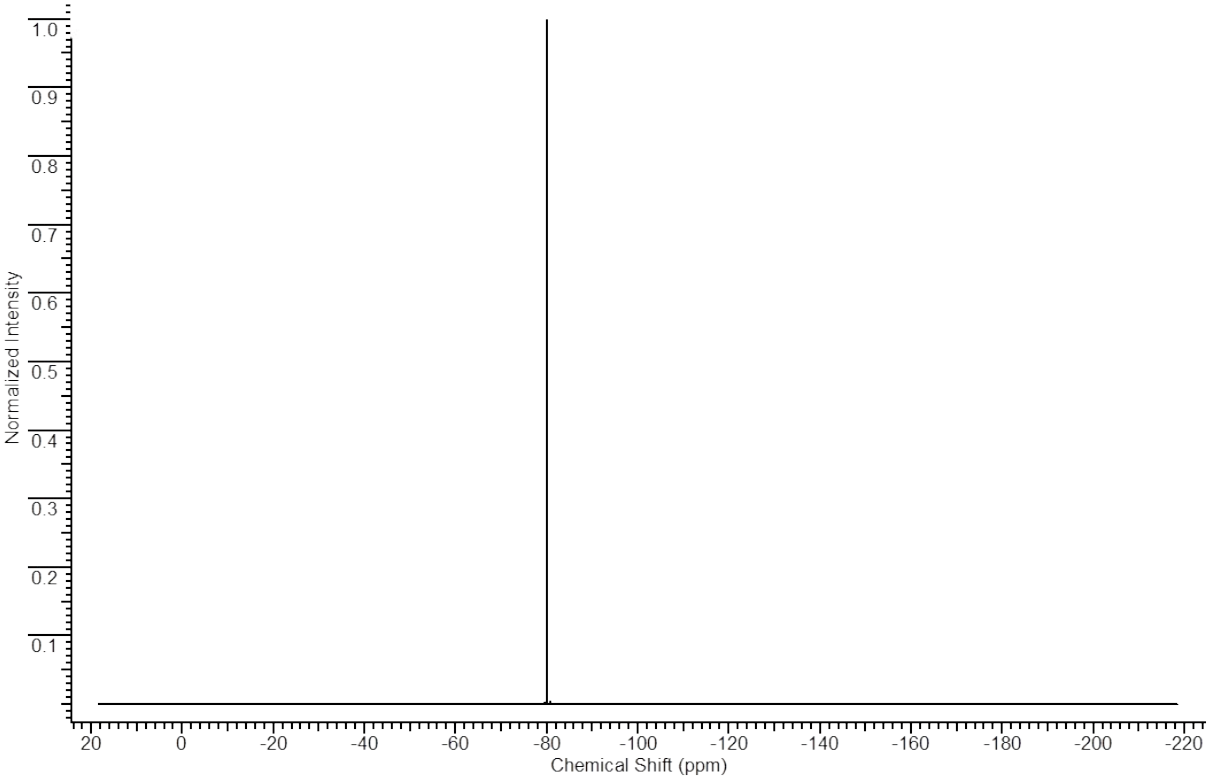

Figure 29: ^{19}F spectrum of compound **5f** (282 MHz, MeOD).

***N*-(Methyl-6-deoxy-2,3,4-*O*-ethyl-β-D-glucopyranoside-6-yl)-pyridinium triflate 5j**

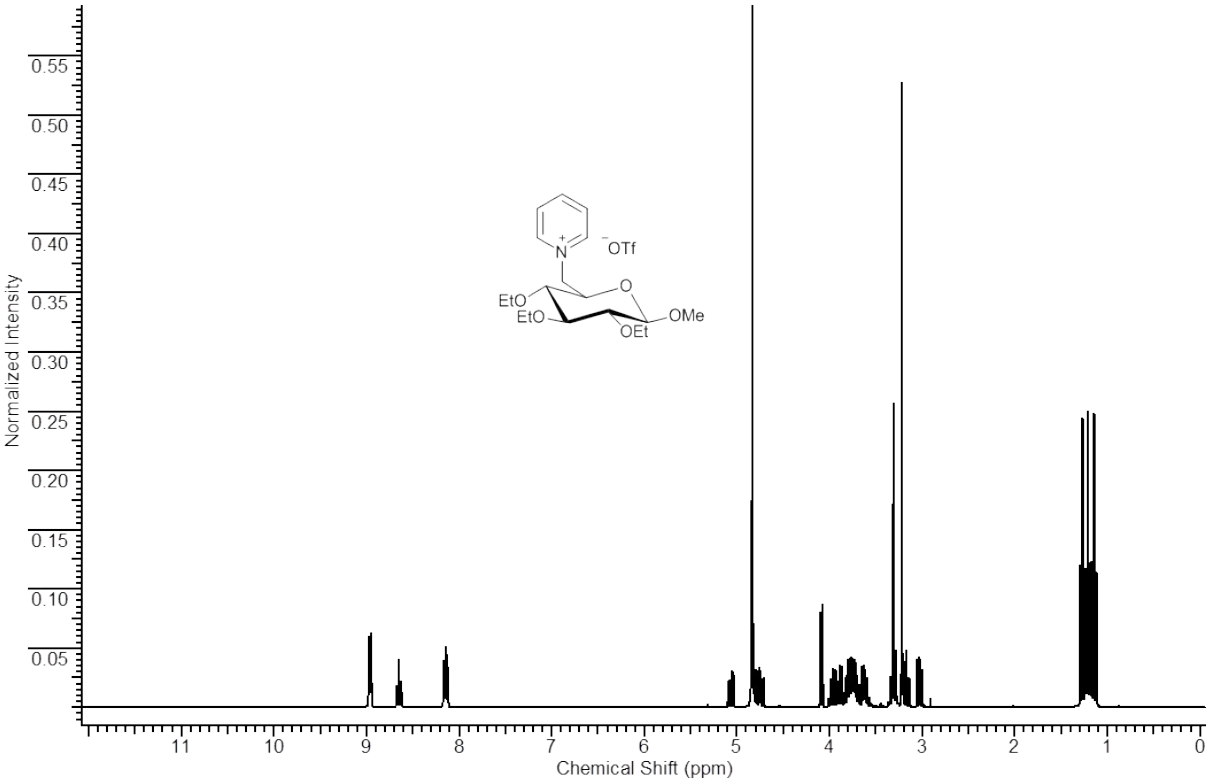

Figure 30: ^{1}H spectrum of compound **5j** (300 MHz, MeOD).

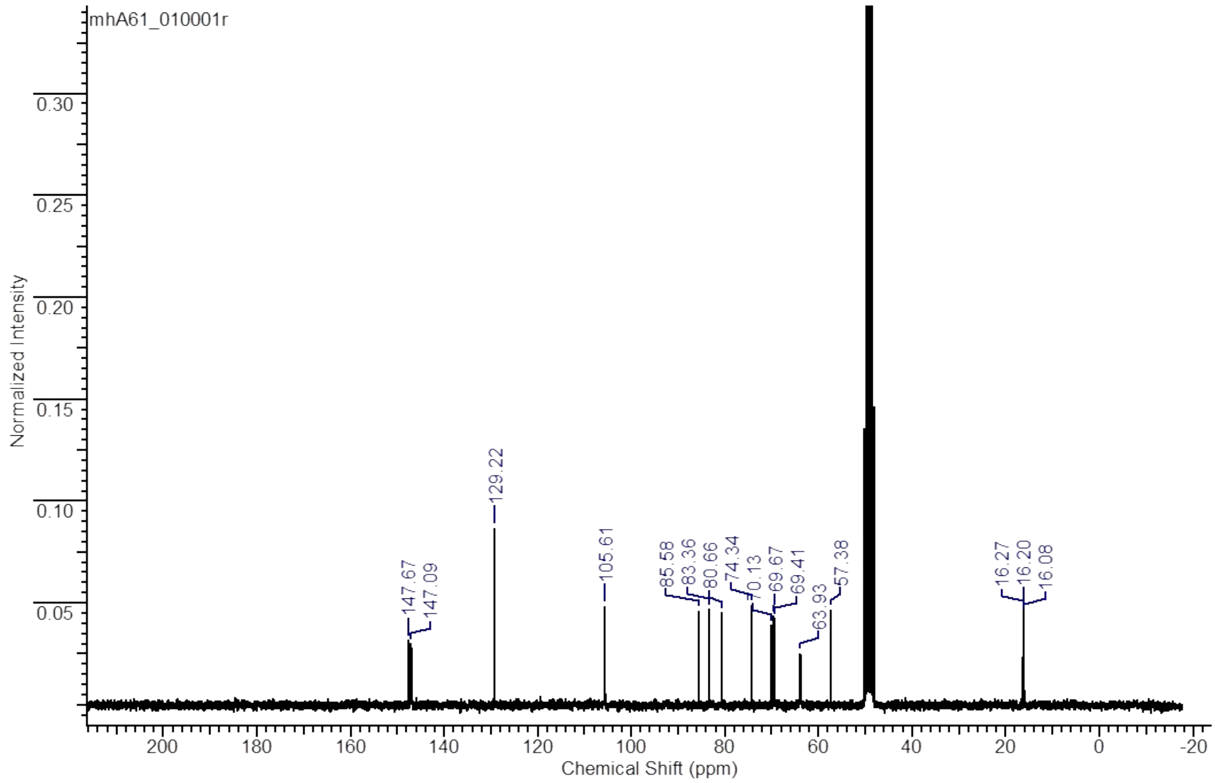

Figure 31: ^{13}C spectrum of compound **5j** (75 MHz, MeOD).

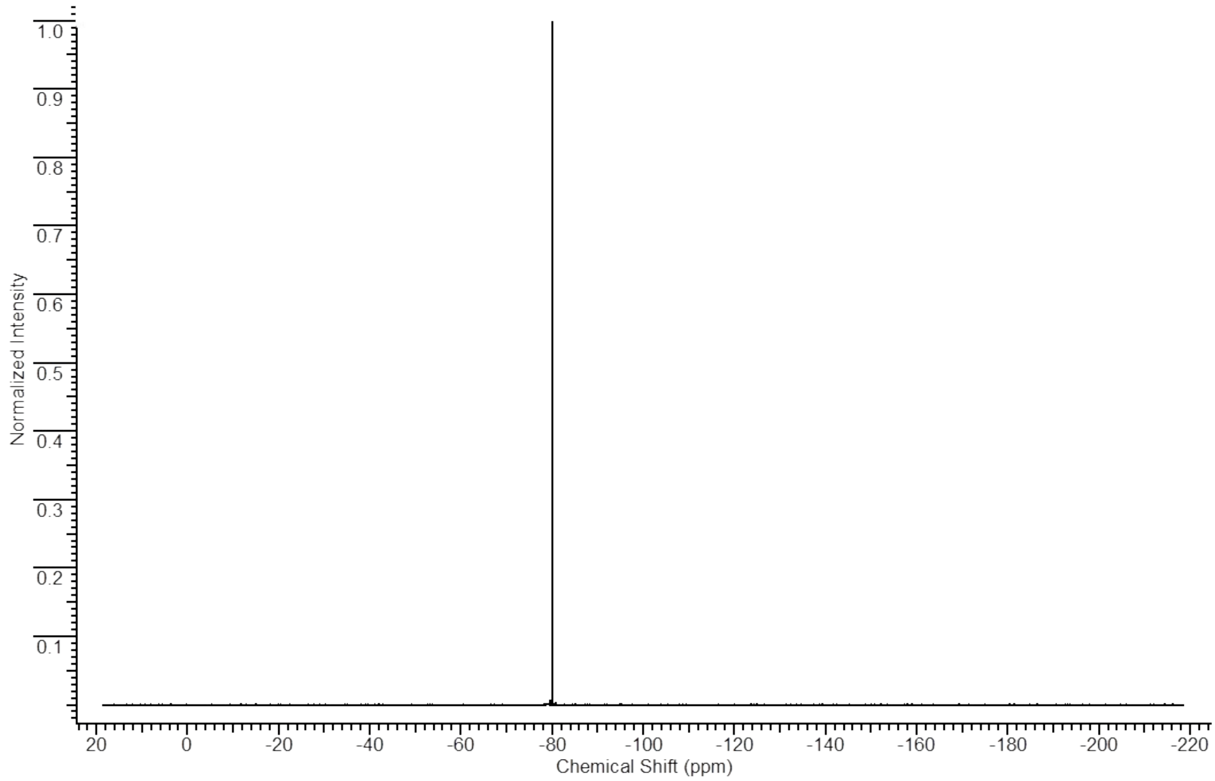

Figure 32: ^{19}F spectrum of compound **5j** (282 MHz, MeOD).

N-(Methyl-6-deoxy-2,3,4-*O*-methyl-β-D-glucopyranoside-6-yl)-pyridinium mesylate 5l

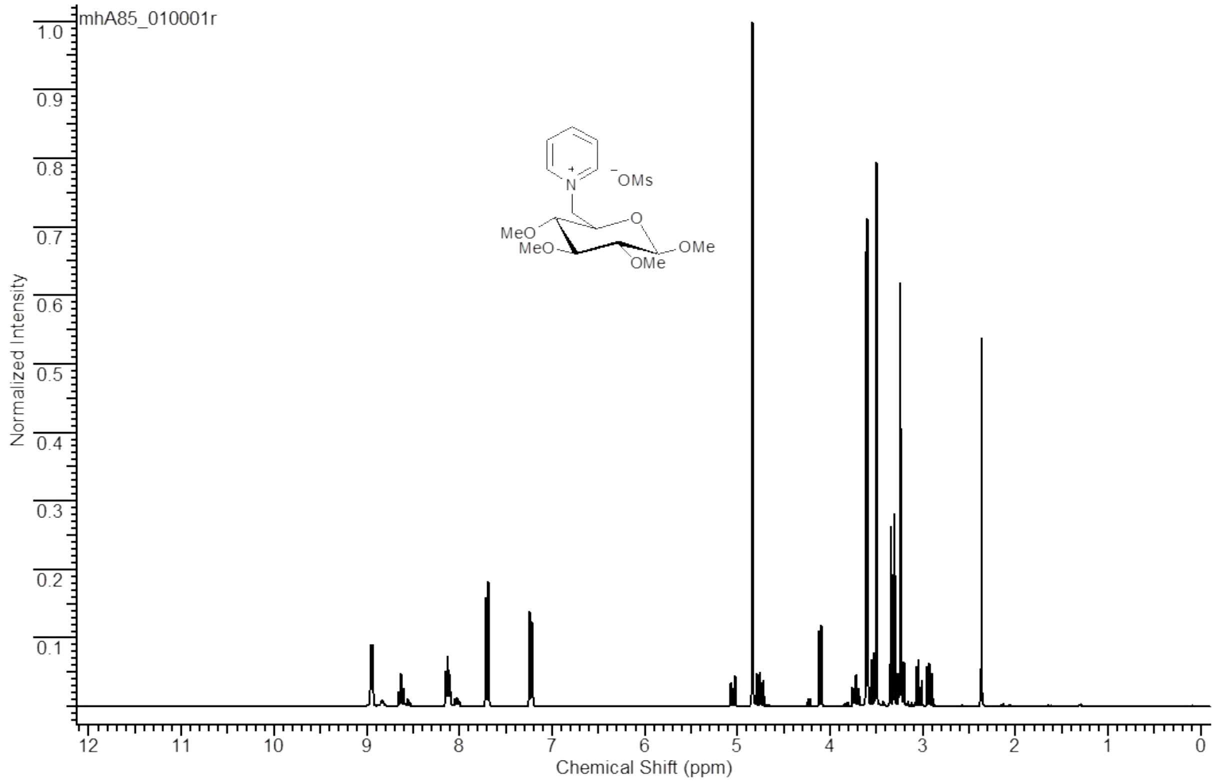

Figure 33: ^{1}H spectrum of compound **5l** (300 MHz, MeOD).

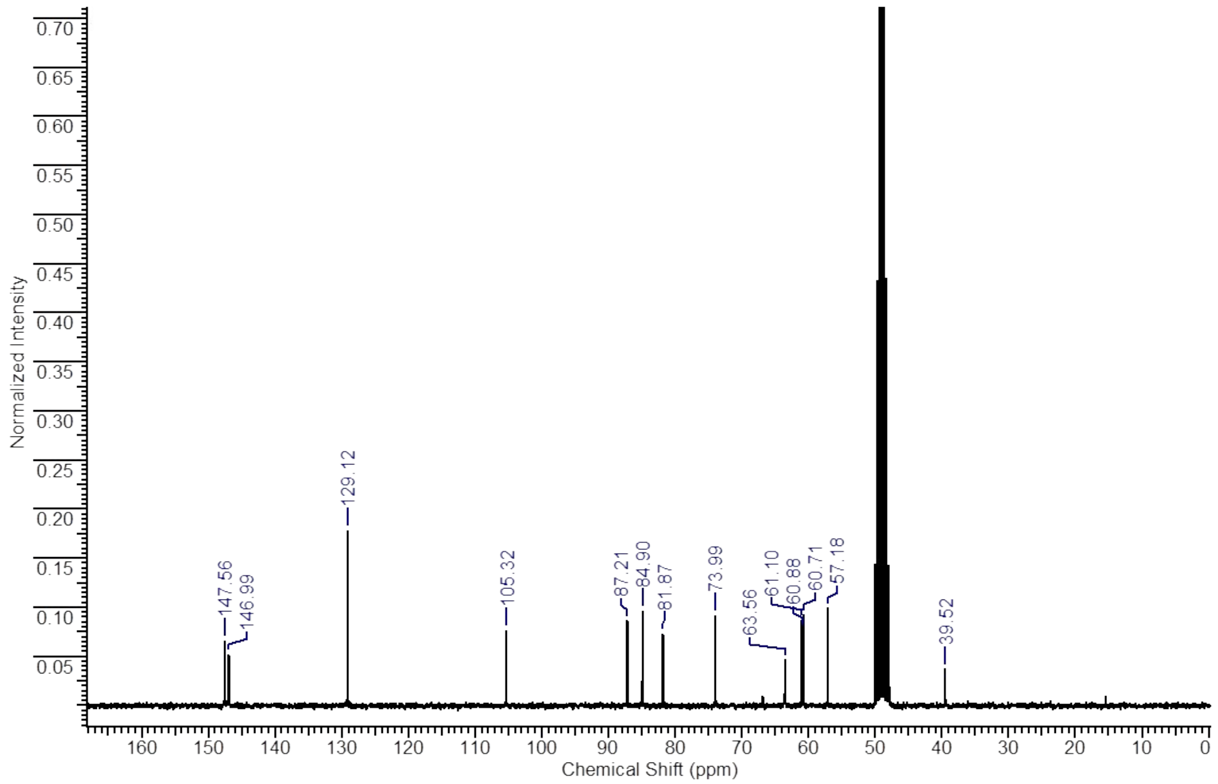

Figure 34: ^{13}C spectrum of compound **5l** (75 MHz, MeOD).

N-(Methyl-6-deoxy-2,3,4-*O*-methyl-β-D-glucopyranoside-6-yl)-pyridinium tosylate 5n

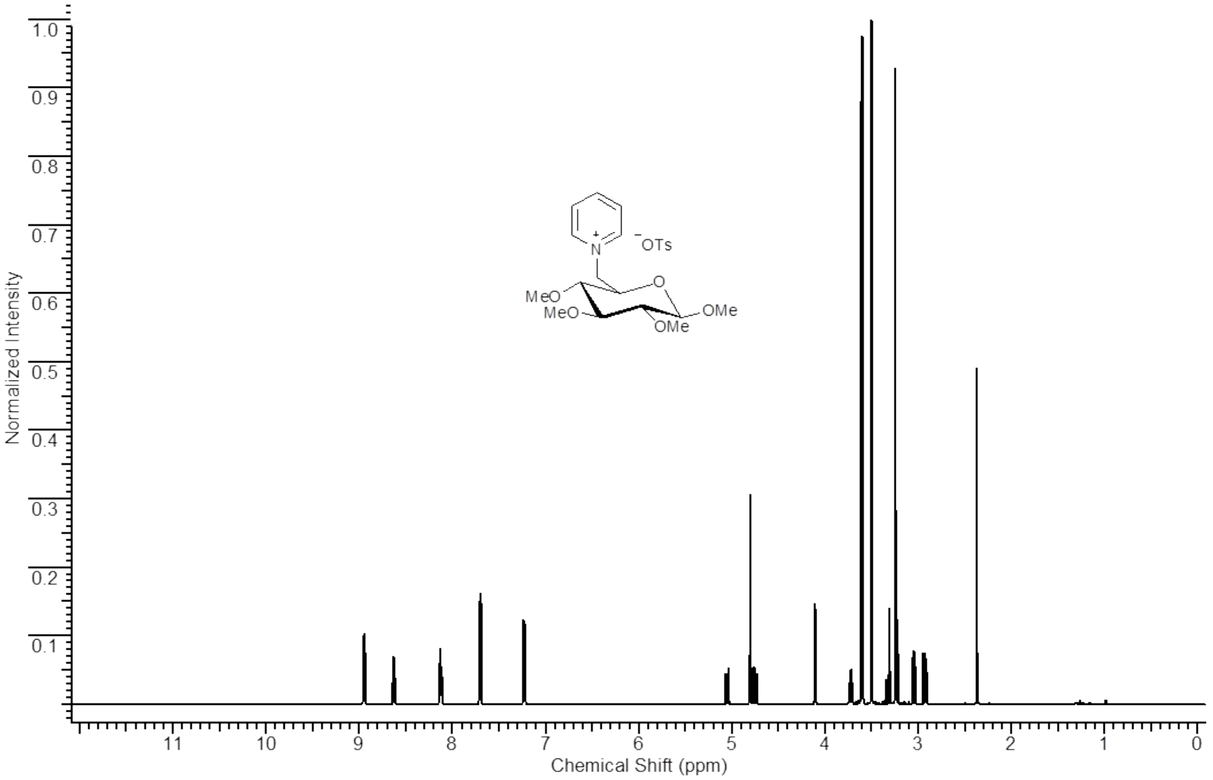

Figure 35: ^{1}H spectrum of compound **5n** (500 MHz, MeOD).

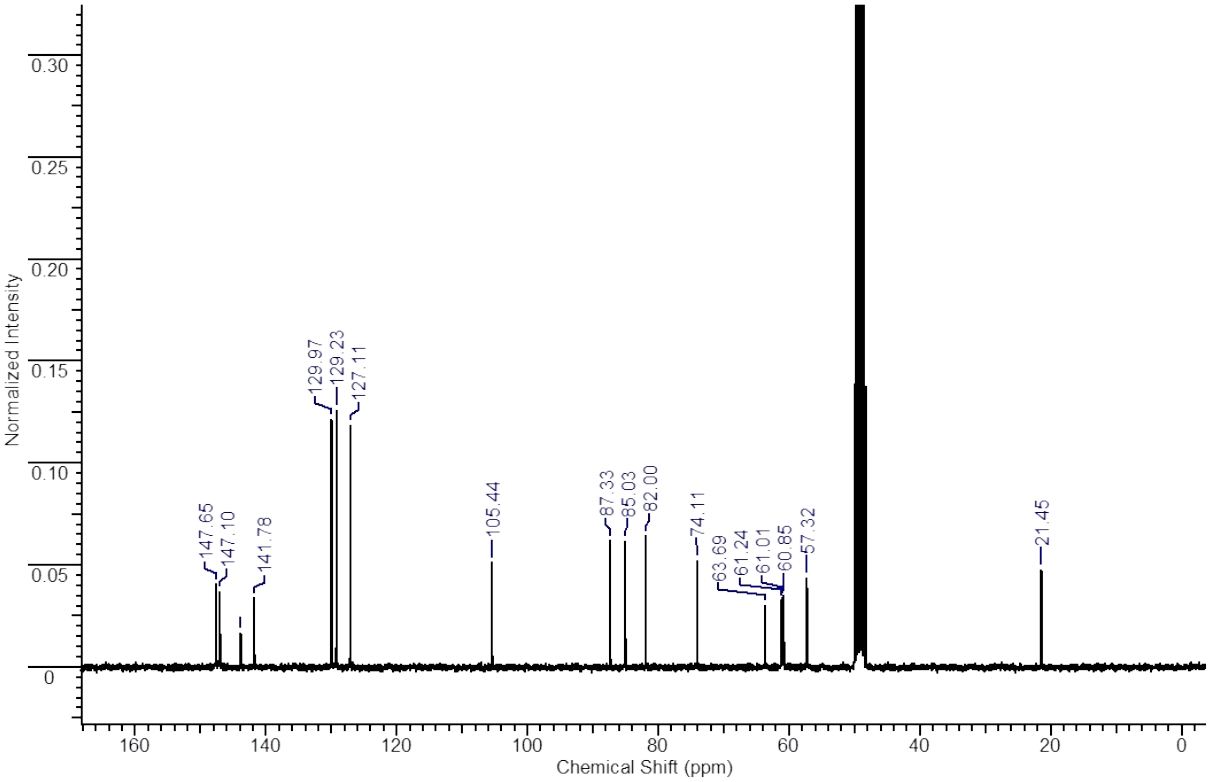

Figure 36: ^{13}C spectrum of compound **5n** (75 MHz, MeOD).

_N_-(Allyl-6-deoxy-2,3,4-_O_-methyl-β-D-glucopyranoside-6-yl)-pyridinium triflate 6f

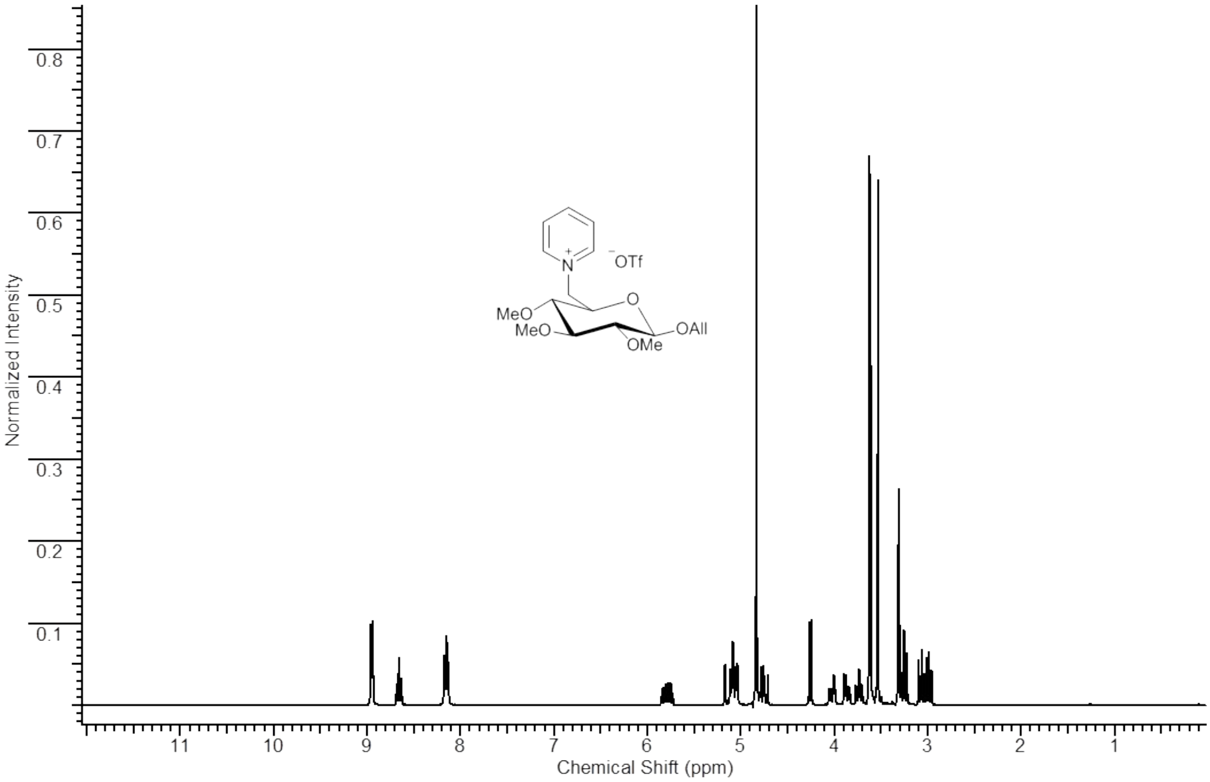

Figure 37: ^{1}H spectrum of compound **6f** (300 MHz, MeOD).

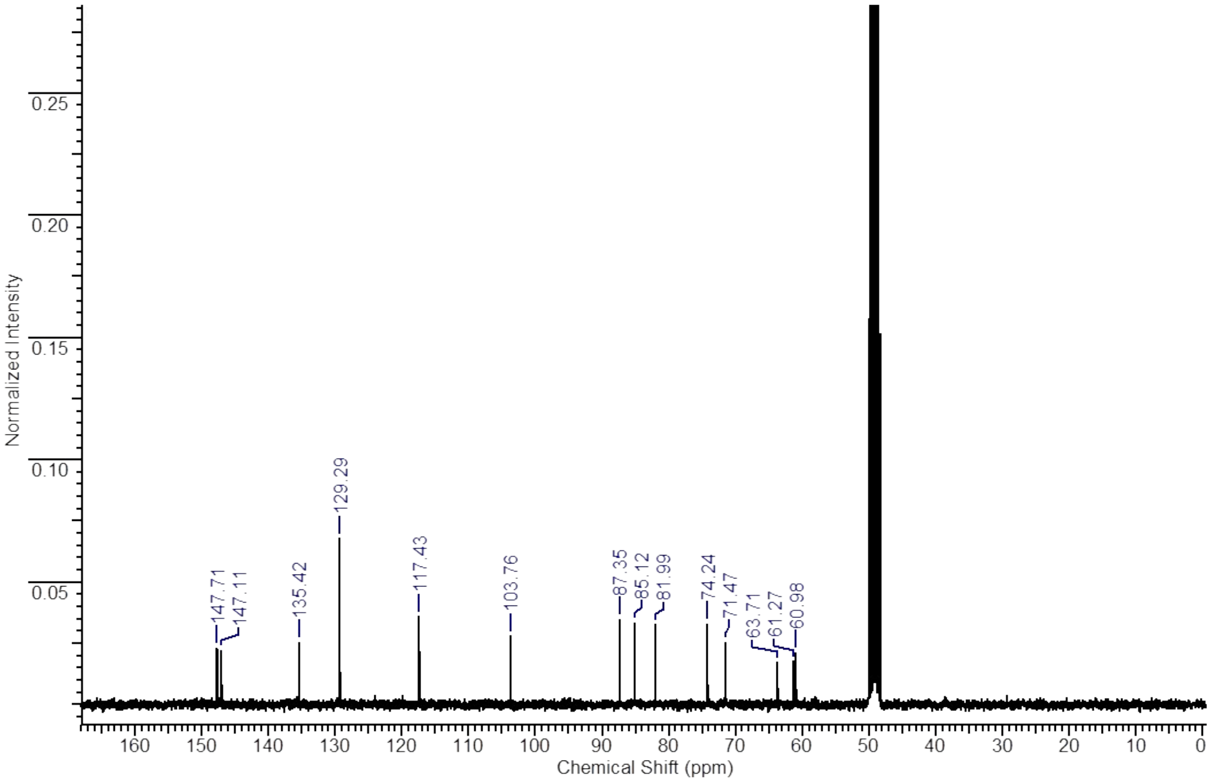

Figure 38: ^{13}C spectrum of compound **6f** (75 MHz, MeOD).

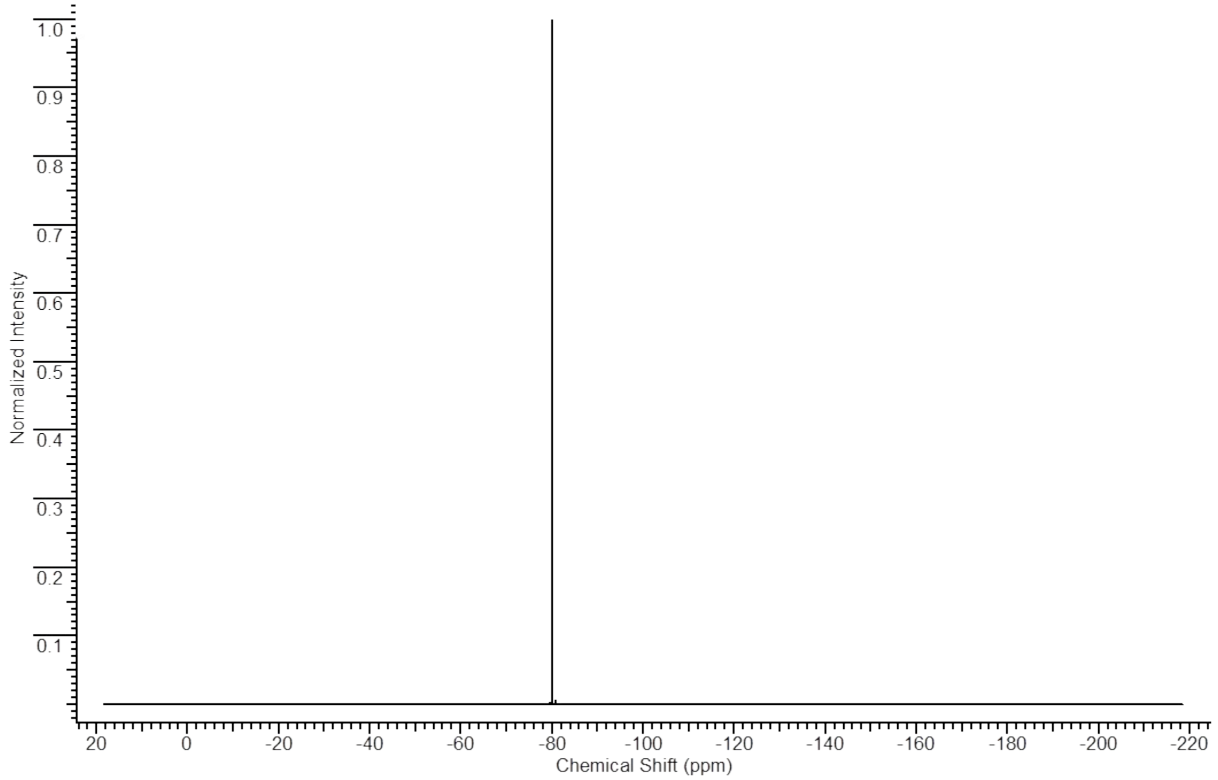

Figure 39: ^{19}F spectrum of compound **6f** (282 MHz, MeOD).

N-(Phenyl-6-deoxy-2,3,4-*O*-methyl-β-D-glucopyranoside-6-yl)-pyridinium triflate 7f

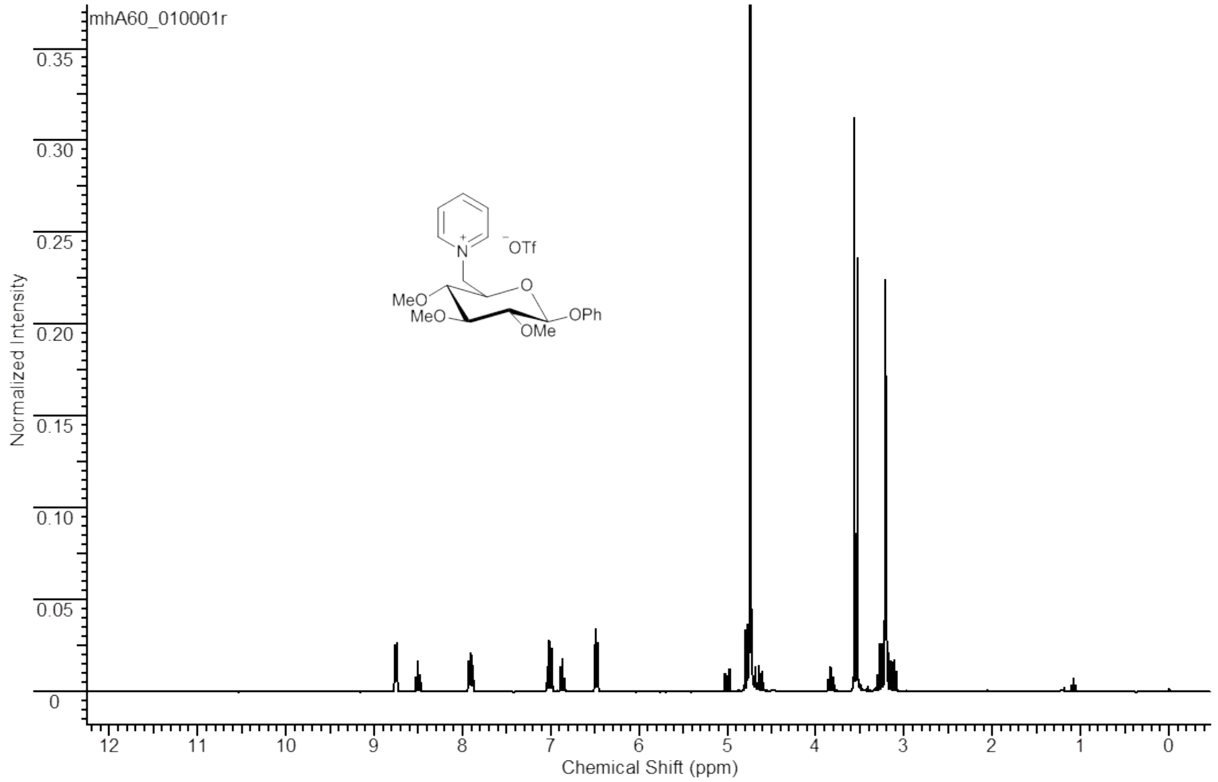

Figure 40: ^{1}H spectrum of compound **7f** (300 MHz, MeOD).

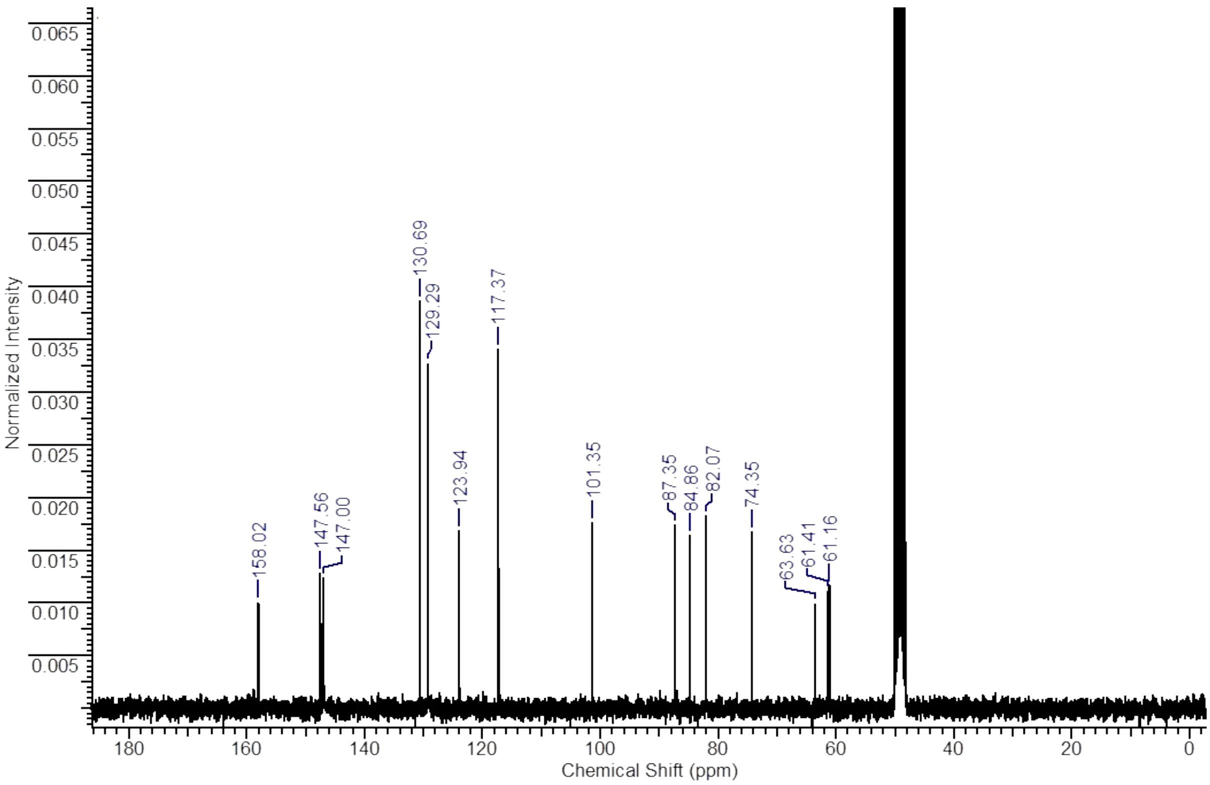

Figure 41: ¹³C spectrum of compound **7f** (75 MHz, MeOD).

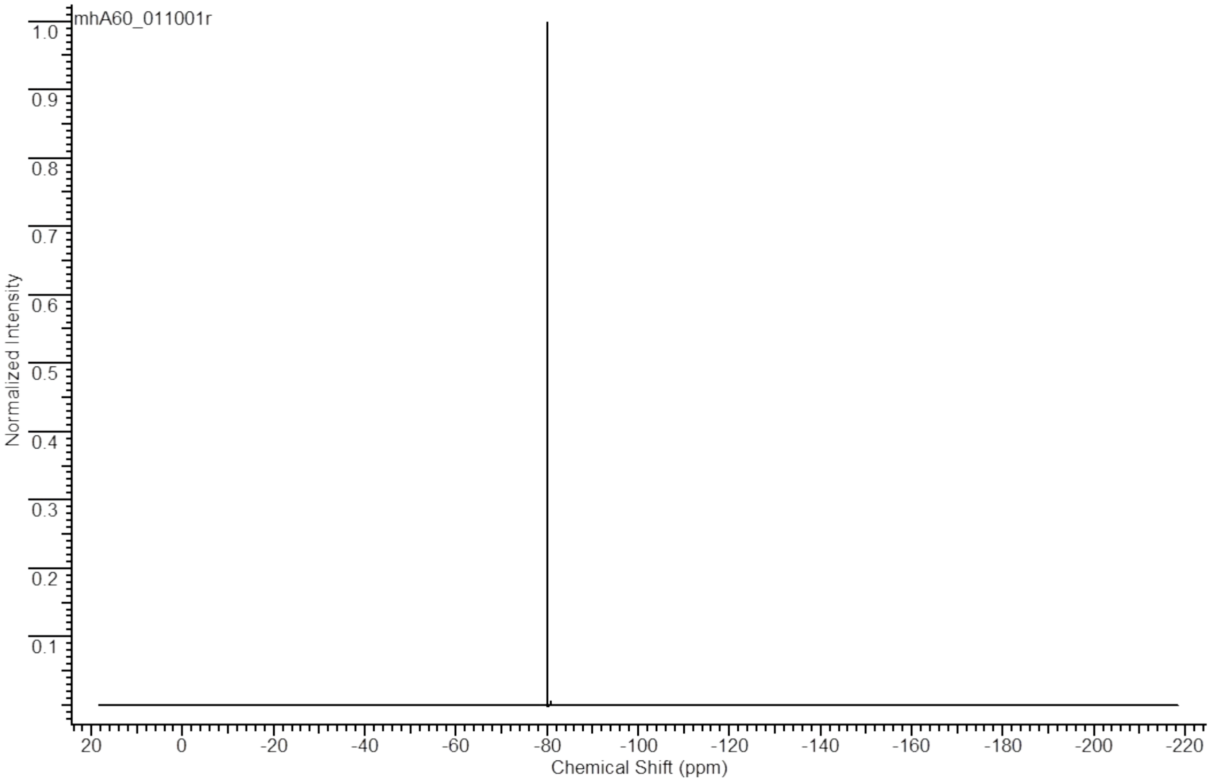

Figure 42: ¹⁹F spectrum of compound **7f** (282 MHz, MeOD).

_N_-(Methyl-6-deoxy-2,3,4-_O_-methyl-α-D-glucopyranoside-6-yl)-pyridinium triflate 8f

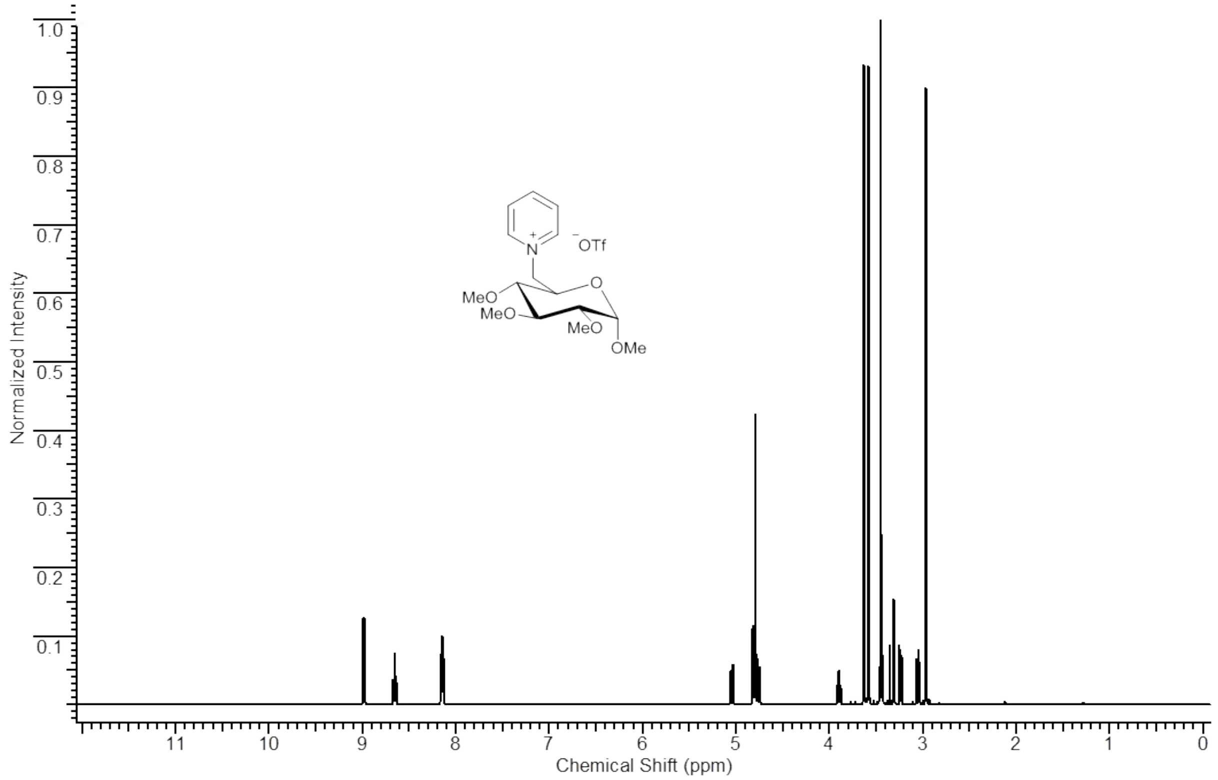

Figure 43: ^{1}H spectrum of compound **8f** (500 MHz, MeOD).

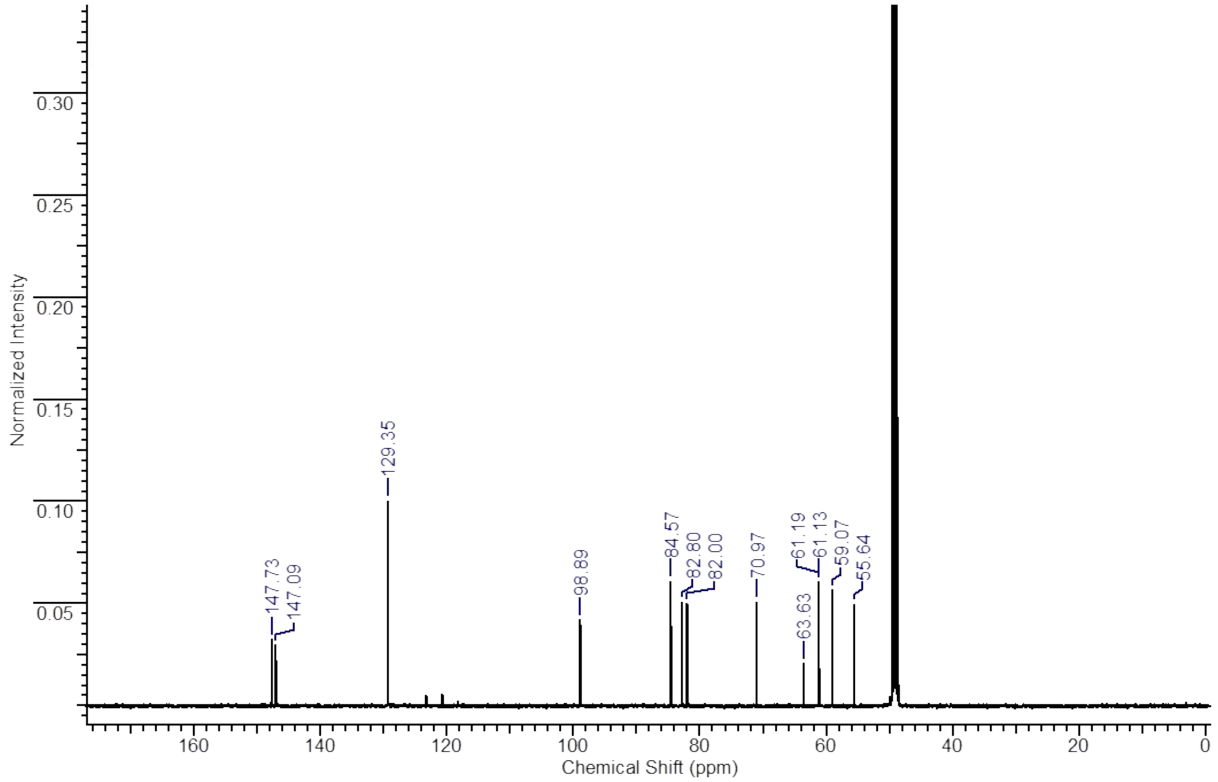

Figure 44: ^{13}C spectrum of compound **8f** (125 MHz, MeOD).

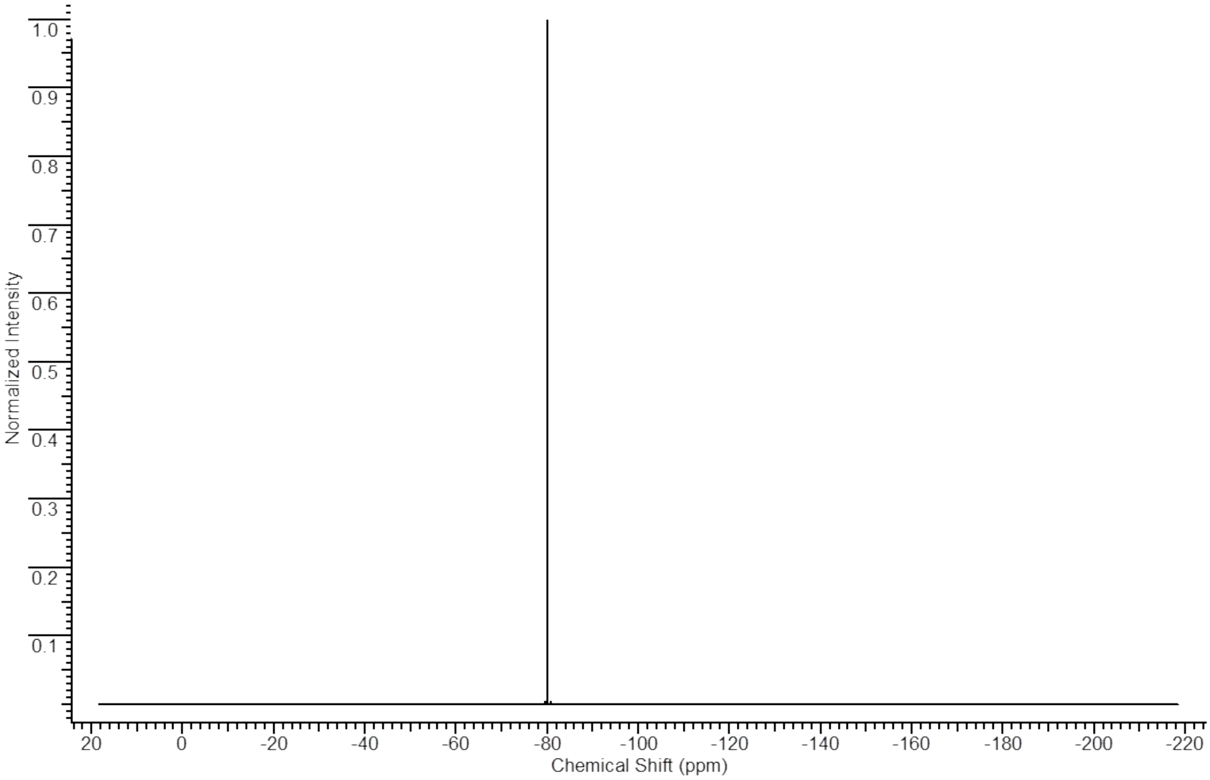

Figure 45: ^{19}F spectrum of compound **8f** (282 MHz, MeOD).

NMR spectra of key pentose intermediates

5-*O*-Trityl-1-deoxy-D-ribofuranoside 1e

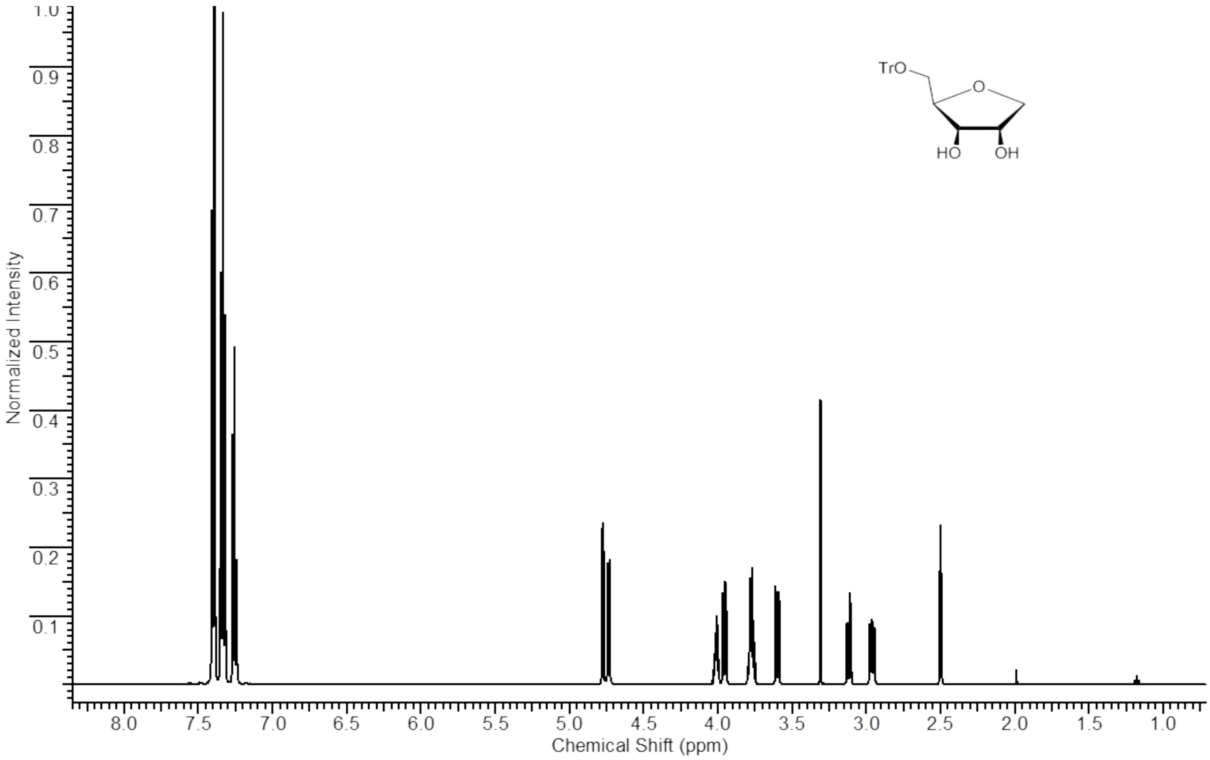

Figure 46: ^{1}H spectrum of compound **1e** (300 MHz in DMSO-*d6*).

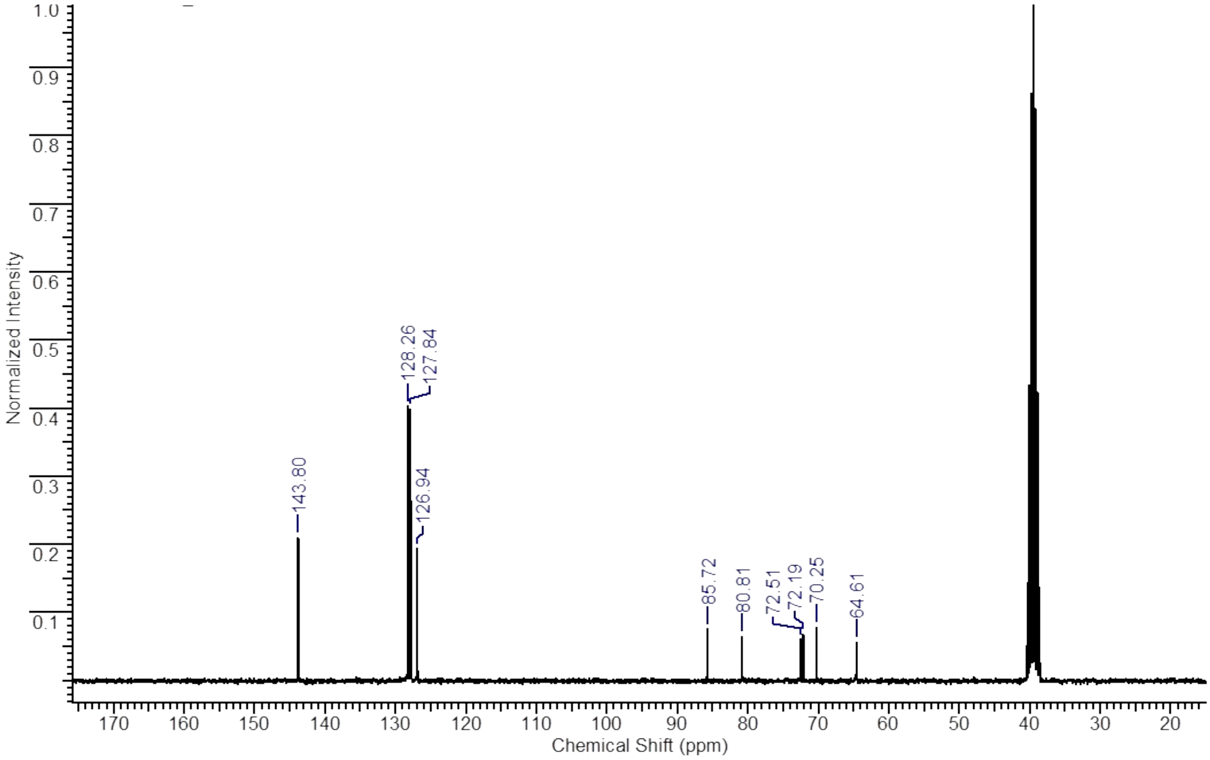

Figure 47: ^{13}C spectrum of compound **1e** (75 MHz, DMSO-*d6*).

5-*O*-Trityl-1-deoxy-D-lyxofuranoside 2e

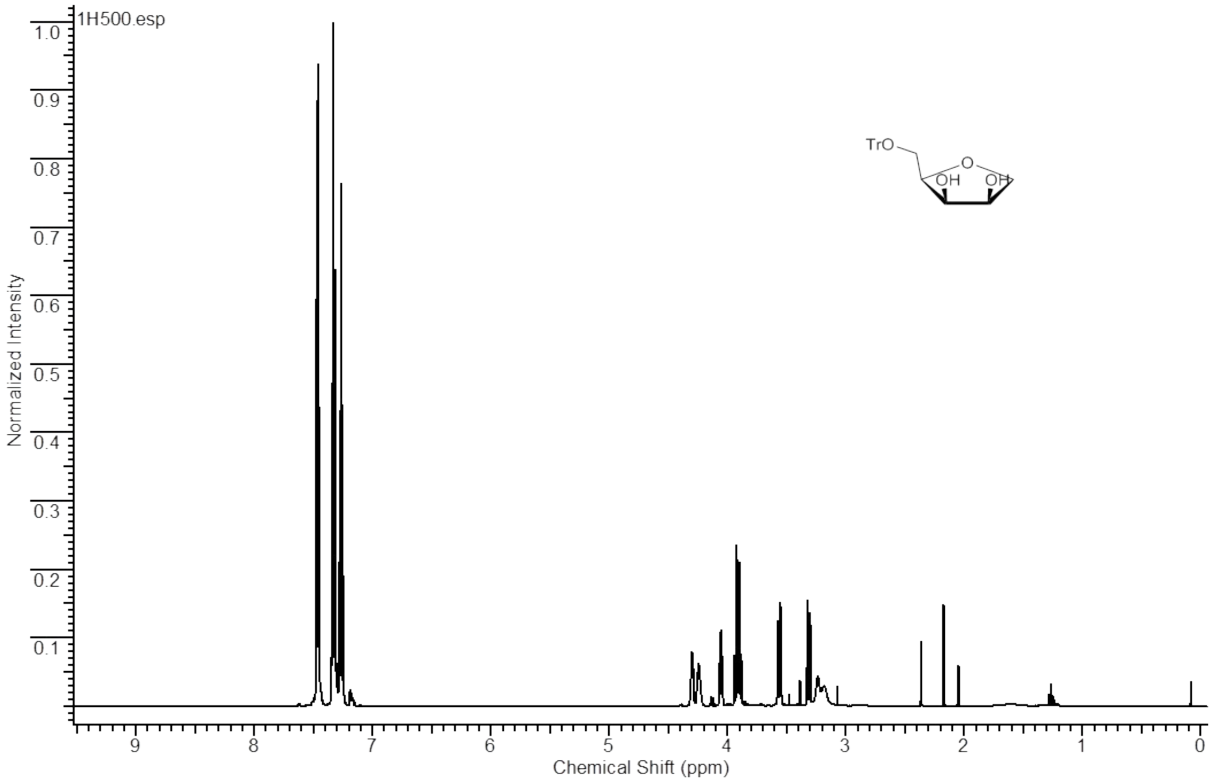

Figure 48: ^{1}H spectrum of compound **2e** (500 MHz, $CDCl_3$).

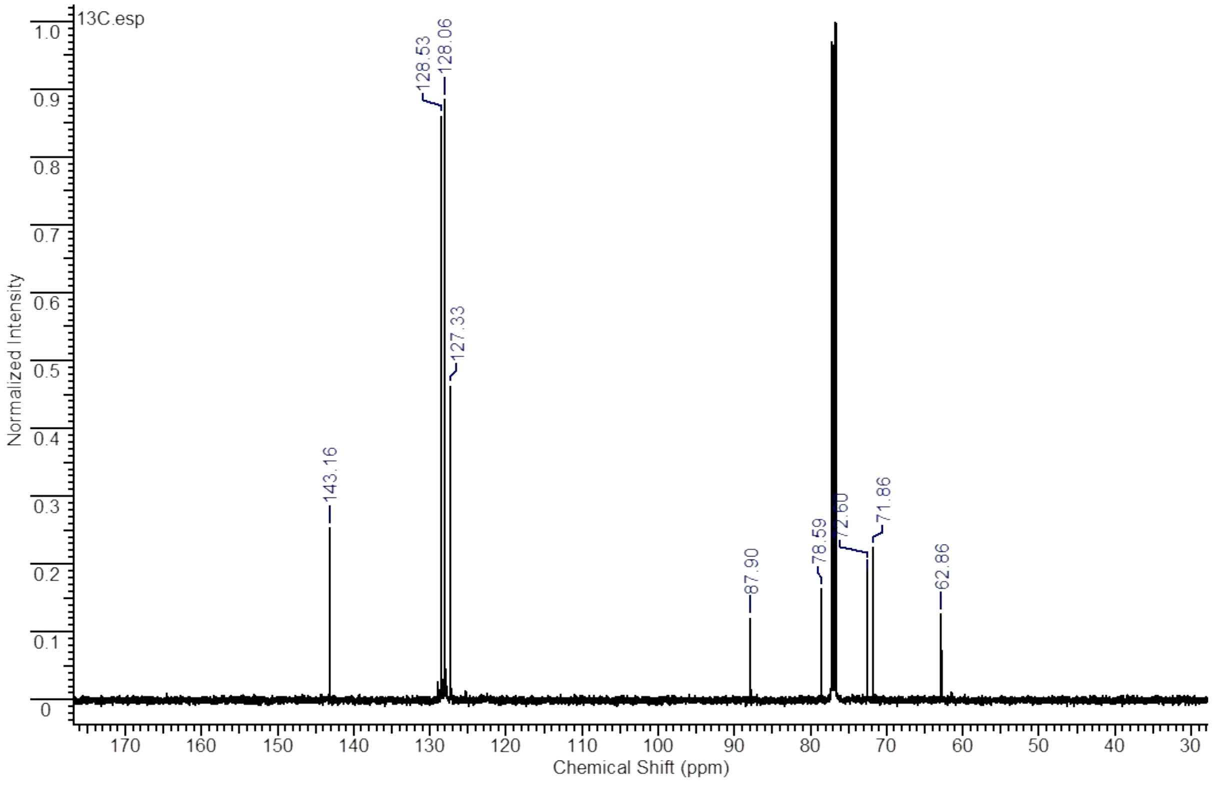

Figure 49: ^{13}C spectrum of compound **2e** (125 MHz, $CDCl_3$).

5-*O*-Trityl-1-deoxy-D-xylofuranoside 3e

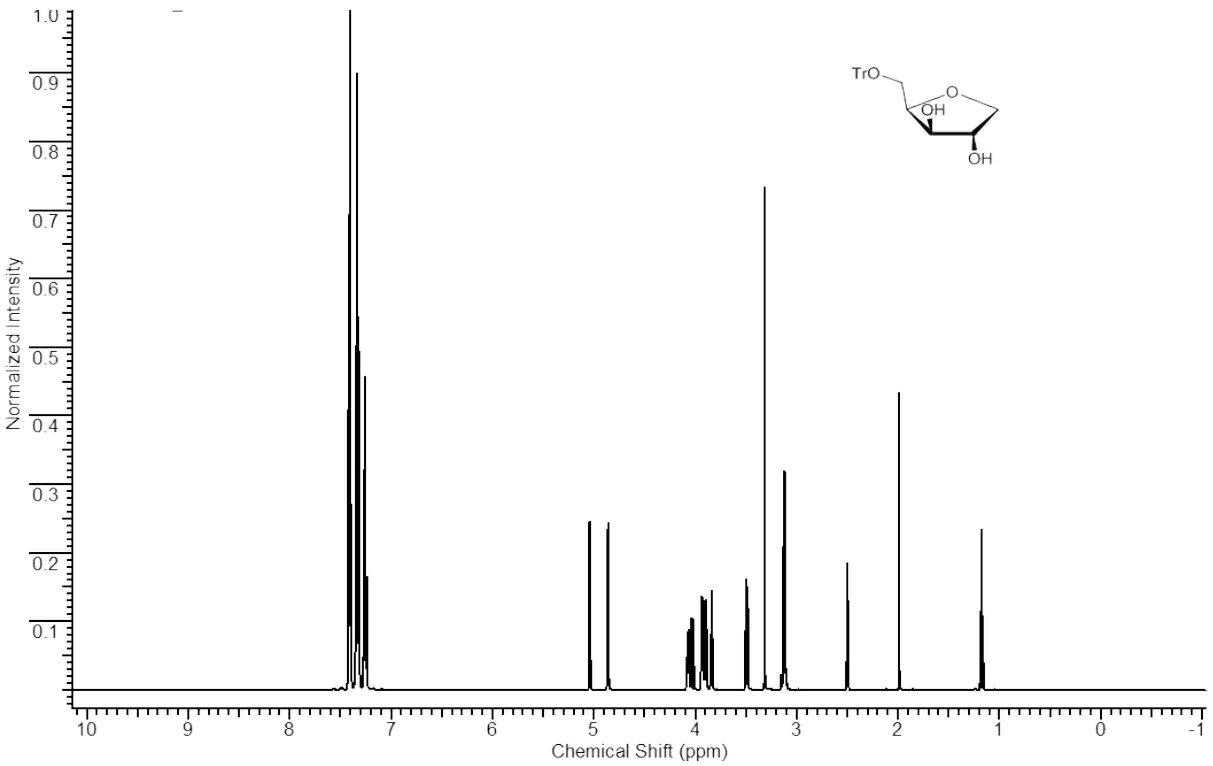

Figure 50: ^{1}H spectrum of compound **3e** (500 MHz, DMSO-*d6*)

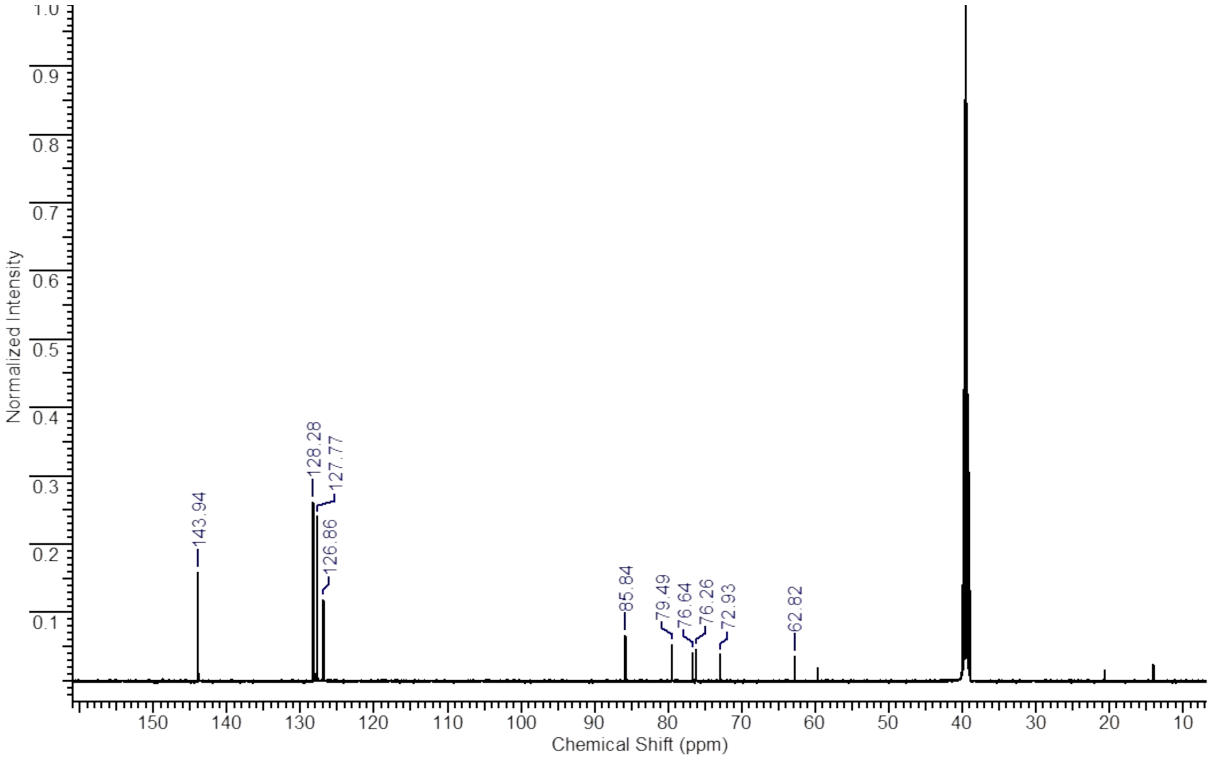

Figure 51: ^{13}C spectrum of compound **3e** (125 MHz, DMSO-*d6*).

5-*O*-Trityl-1-deoxy-L-arabinofuranoside 4e

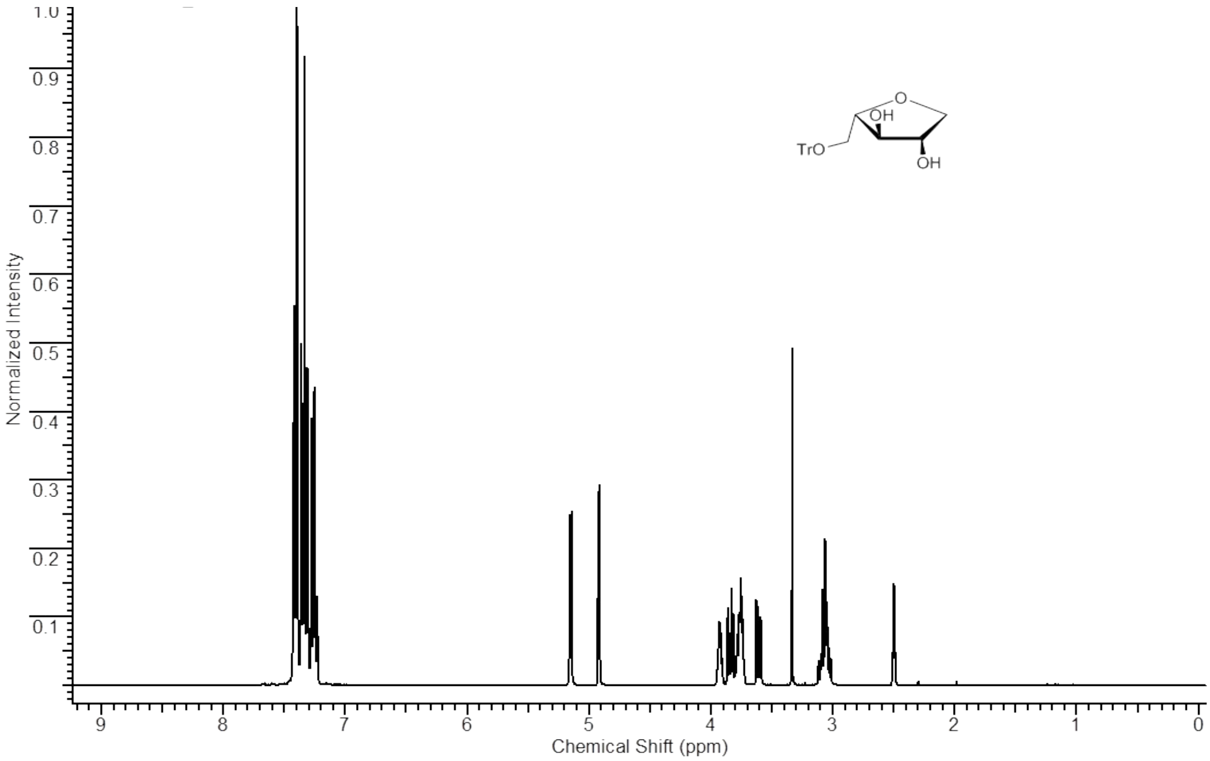

Figure 52: ^{1}H spectrum of compound **4e** (300 MHz, DMSO-*d6*).

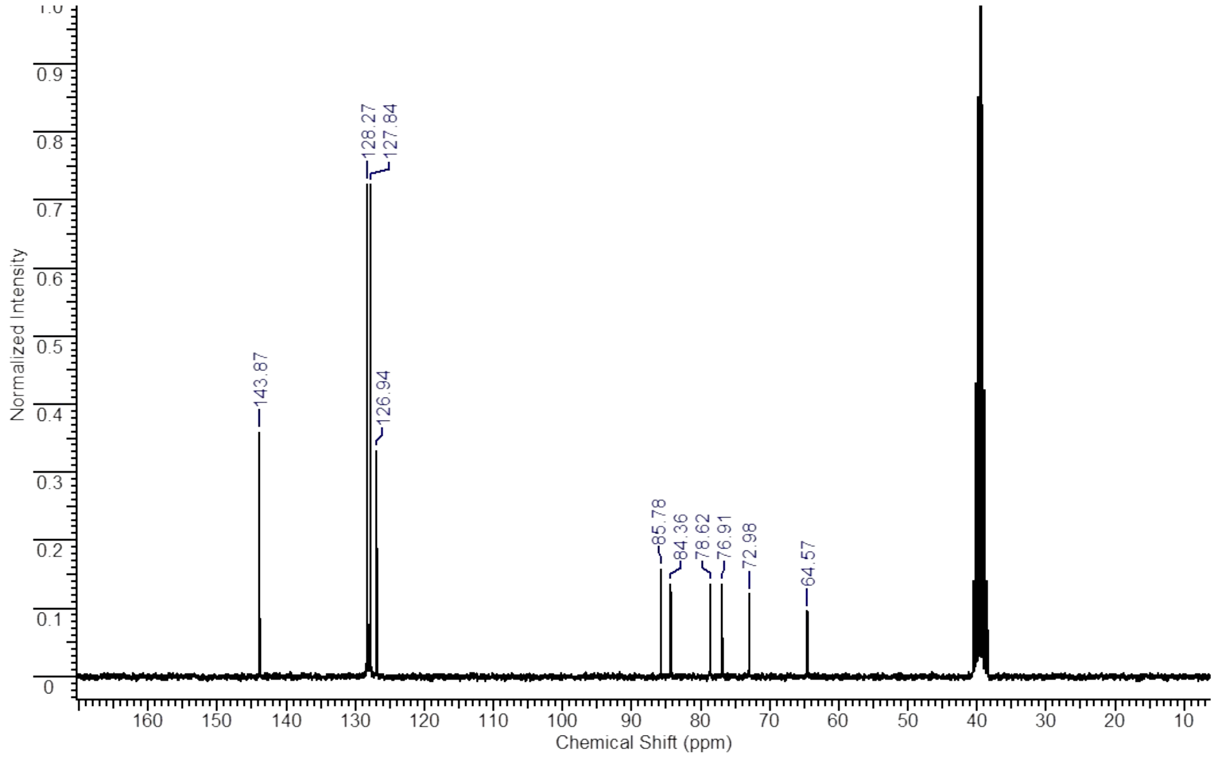

Figure 53: ^{13}C spectrum of compound **4e** (75 MHz, DMSO-*d6*).

2,3-*O*-Isopropylidene-1-deoxy-D-ribofuranoside 1j

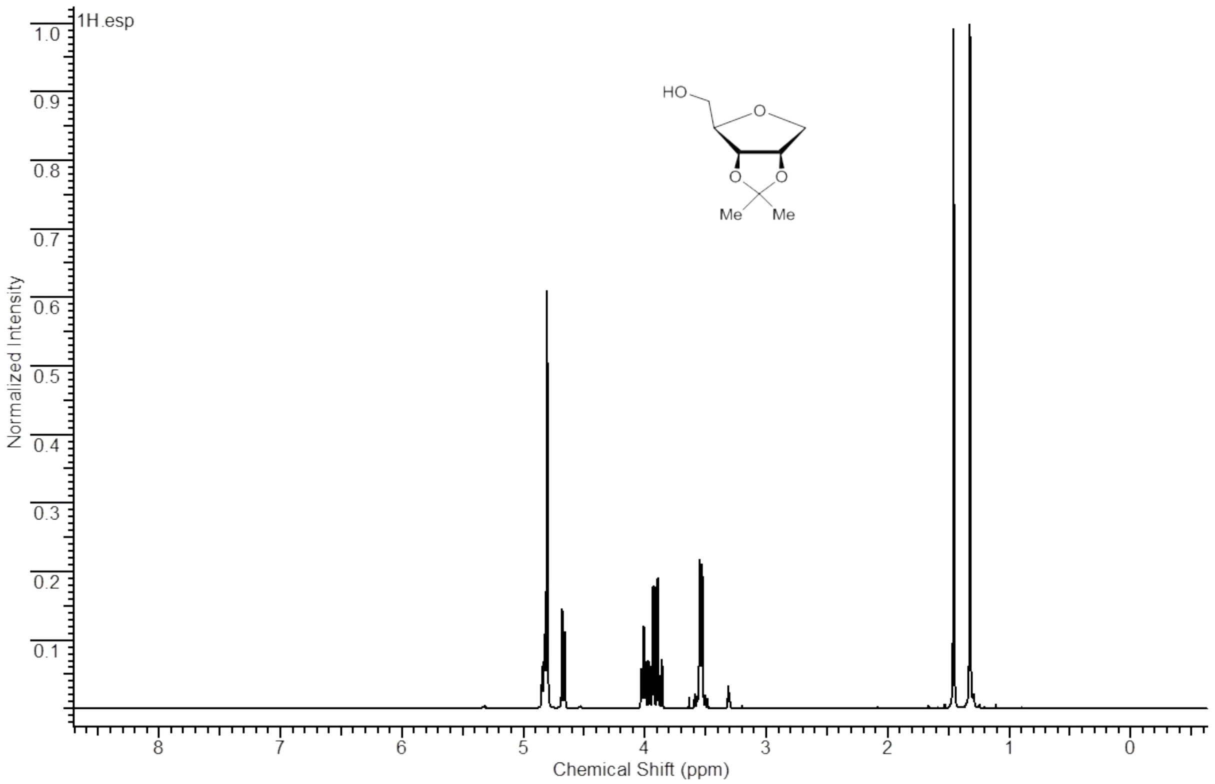

Figure 54: ^{1}H spectrum of compound **1j** (300 MHz, $CDCl_3$).

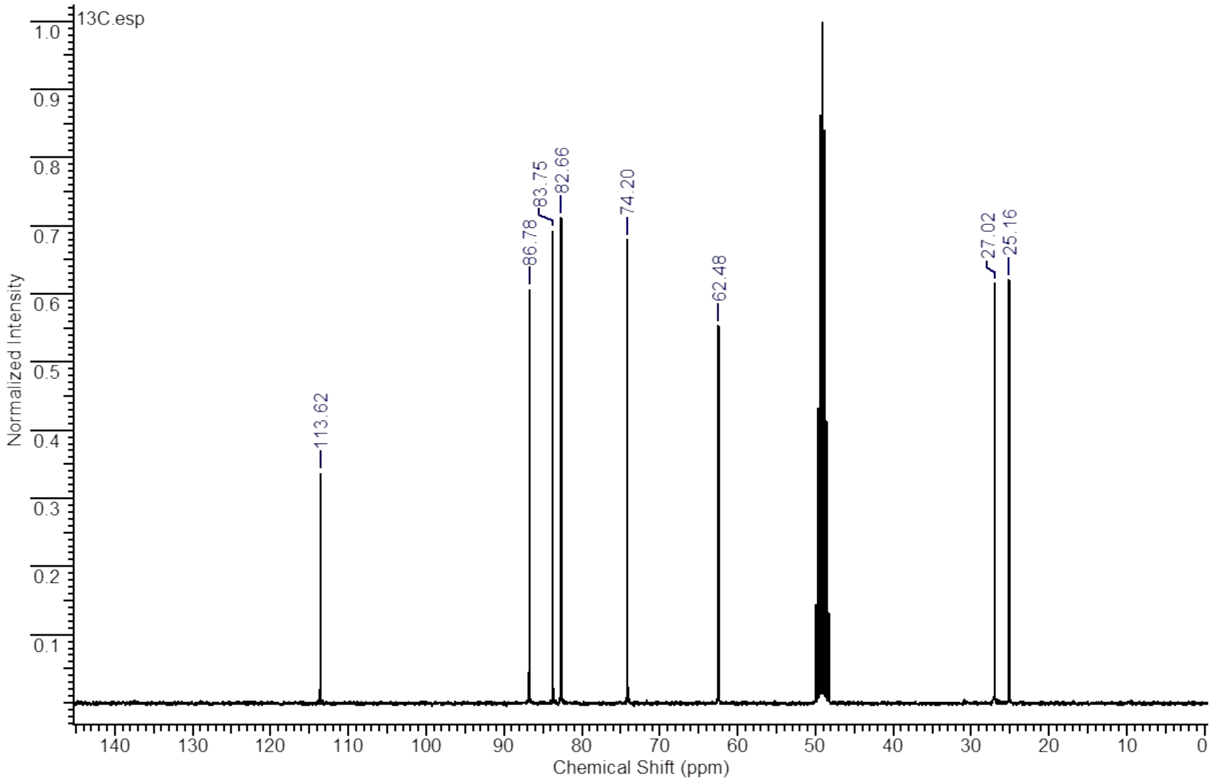

Figure 55: ^{13}C spectrum of compound **1j** (75 MHz, $CDCl_3$).

NMR Spectra of key glucoside intermediates

Methyl-2,3,4-*O*-methyl-β-D-glucopyranoside 5d

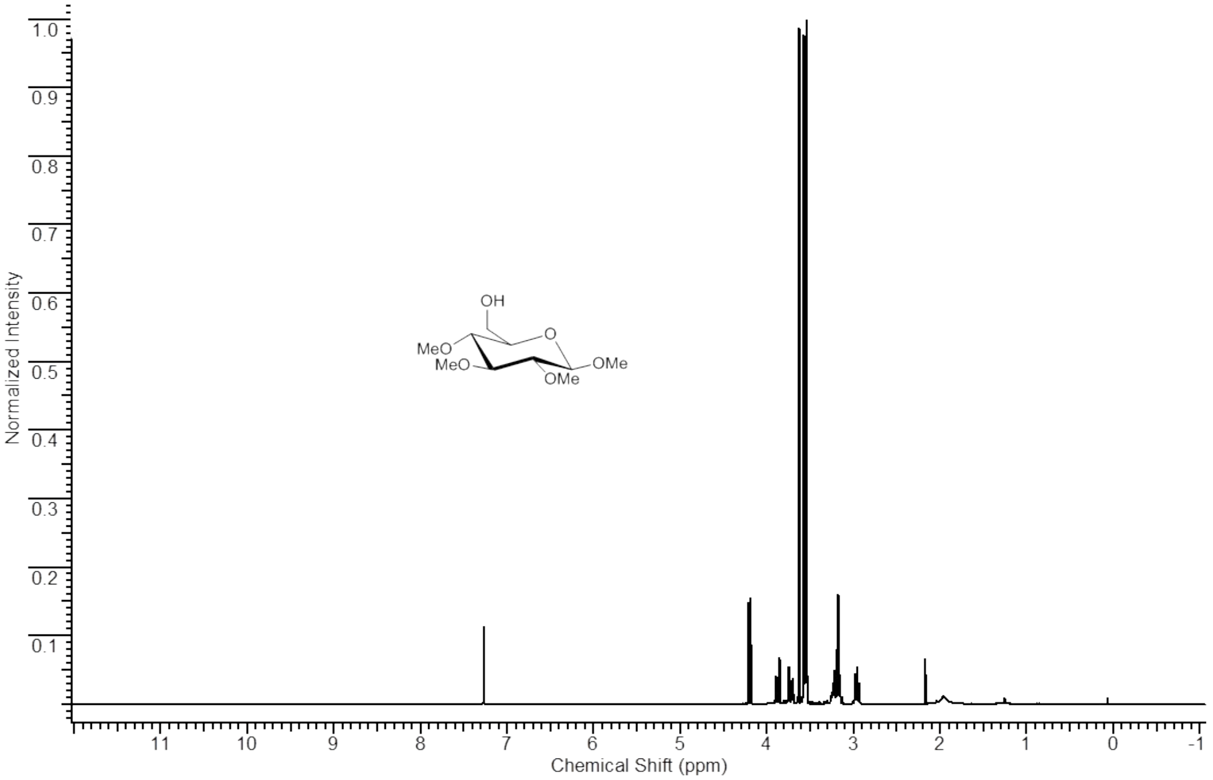

Figure 56: ^{1}H spectrum of compound **5d** (300 MHz, $CDCl_3$).

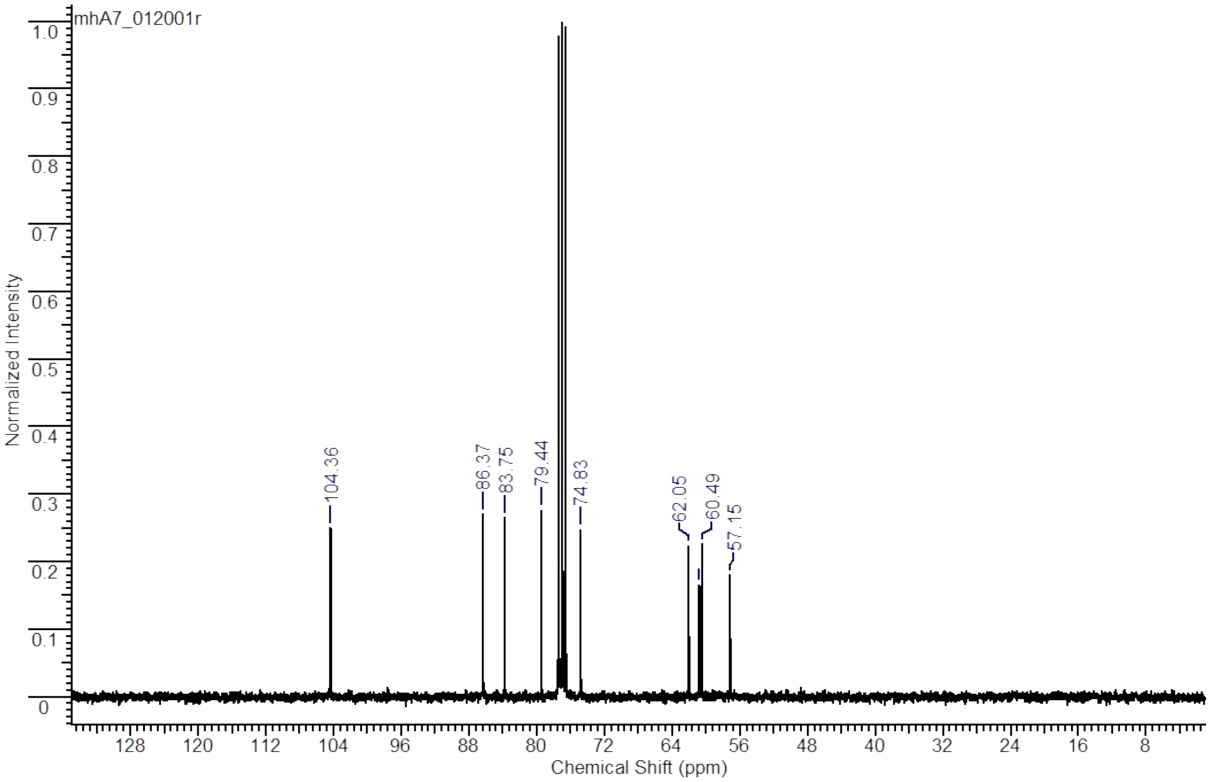

Figure 57: ^{13}C spectrum of compound **5d** (75 MHz, $CDCl_3$).

Allyl-2,3,4-*O*-methyl-β-D-glucopyranoside 6d

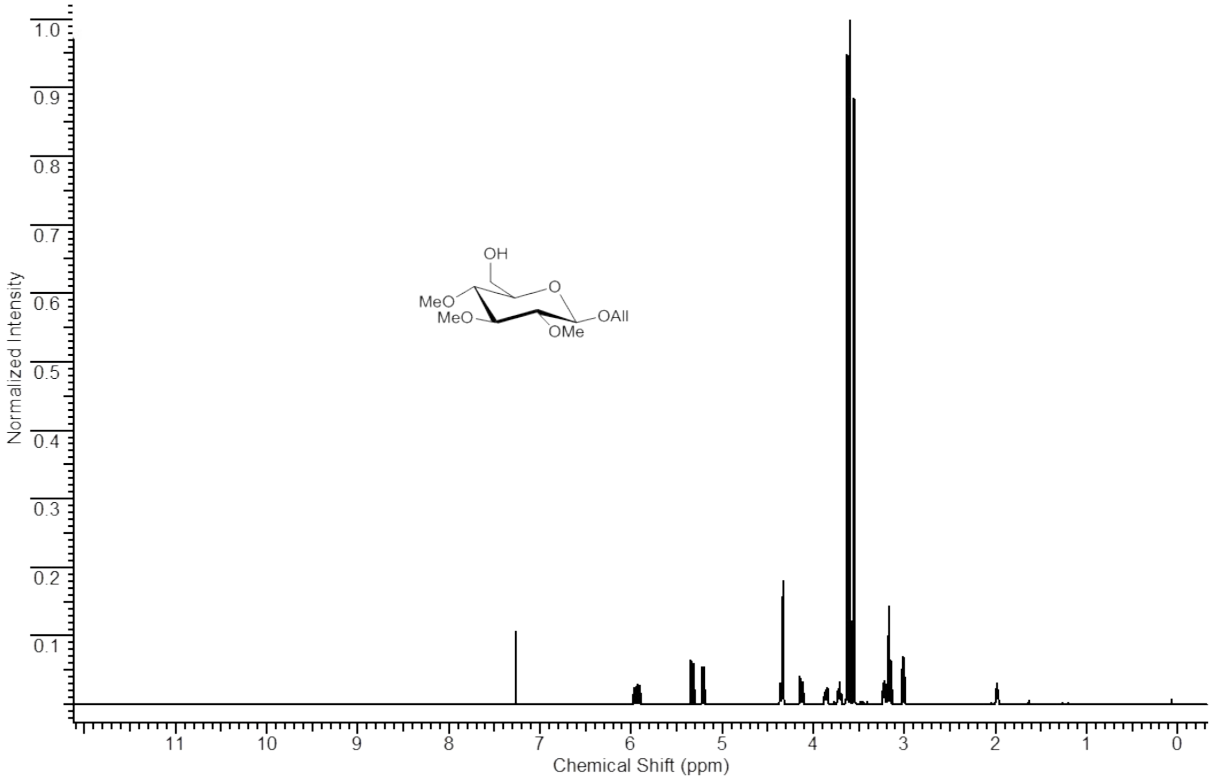

Figure 58: ^{1}H spectrum of compound **6d** (500 MHz, $CDCl_3$).

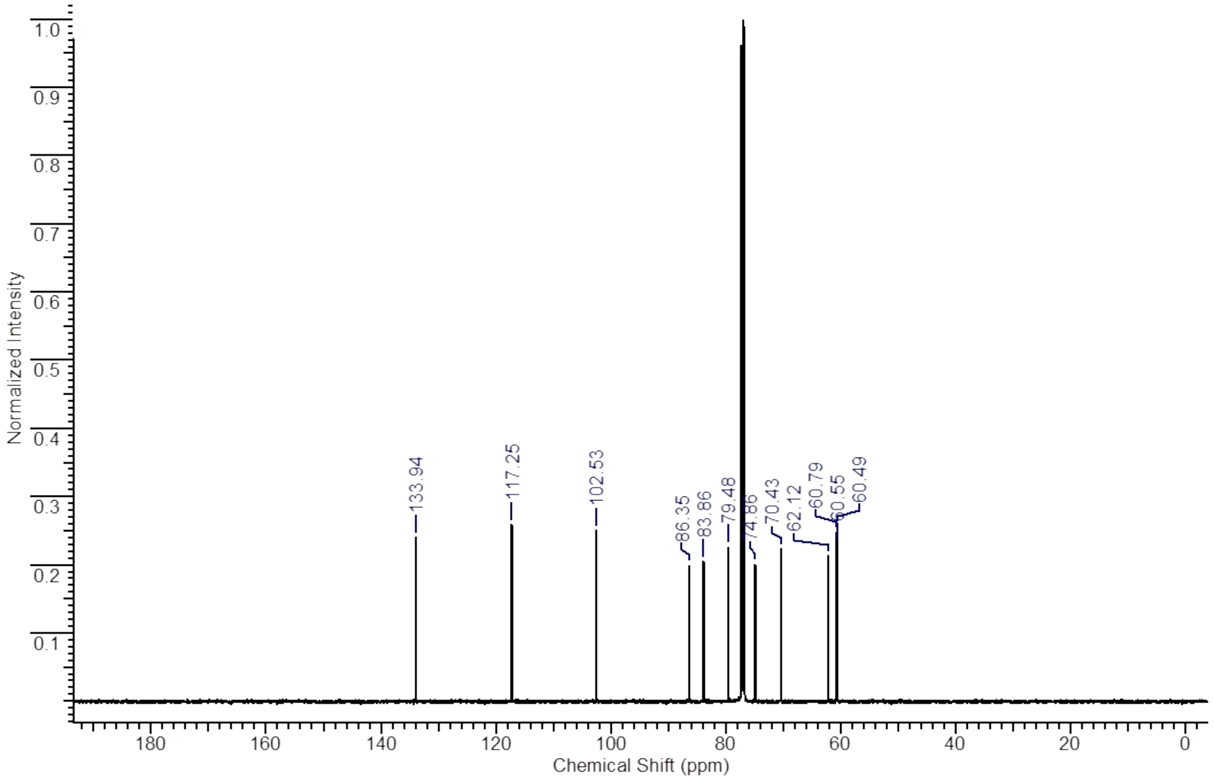

Figure 59: ^{13}C spectrum of compound **6d** (125 MHz, $CDCl_3$).

Phenyl-2,3,4-*O*-methyl-β-D-glucopyranoside 7d

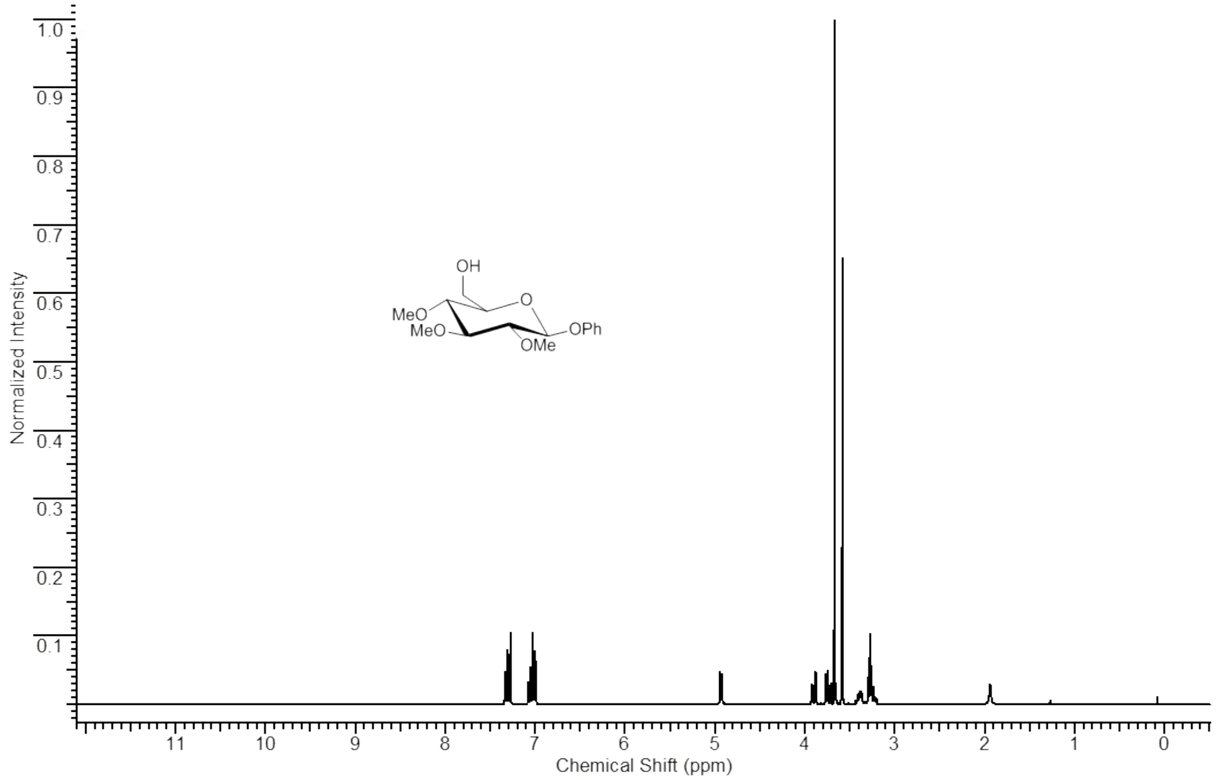

Figure 60: ^{1}H spectrum of compound **7d** (300 MHz, $CDCl_3$).

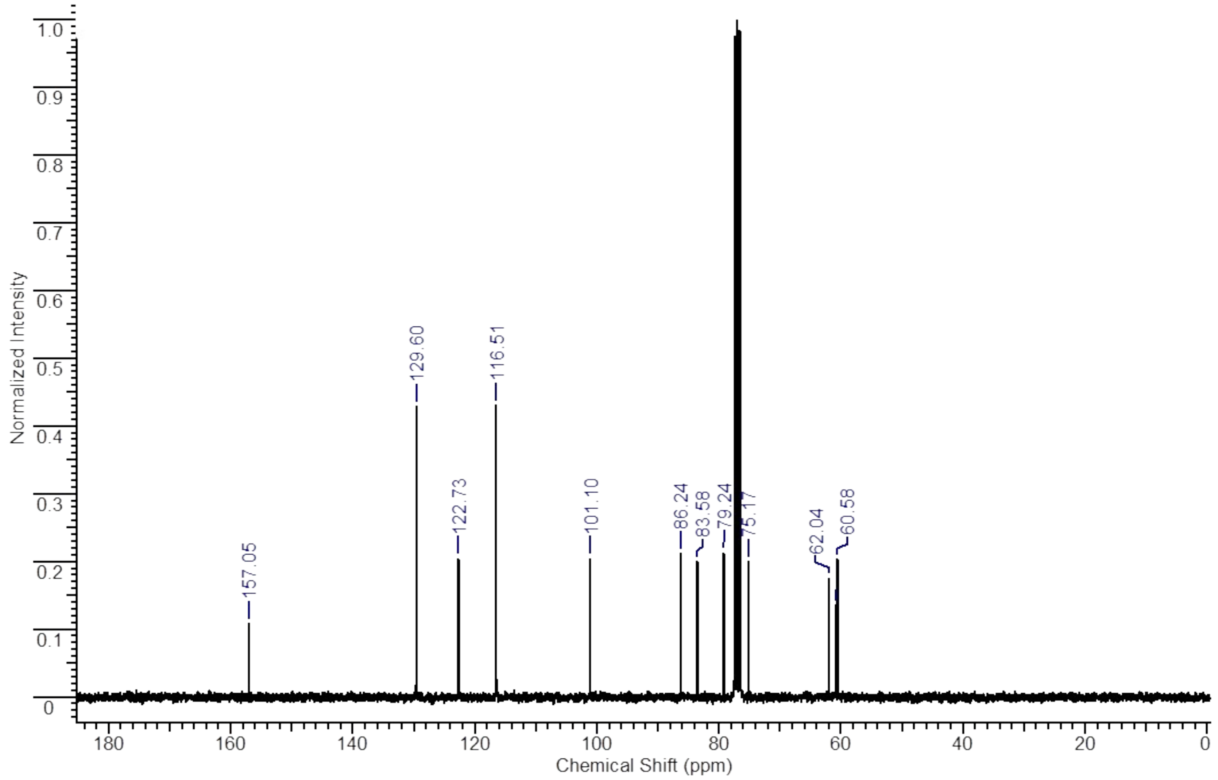

Figure 61: ^{13}C spectrum of compound **7d** (75 MHz, $CDCl_3$).

Methyl-2,3,4-*O*-methyl-α-D-glucopyranoside 8d

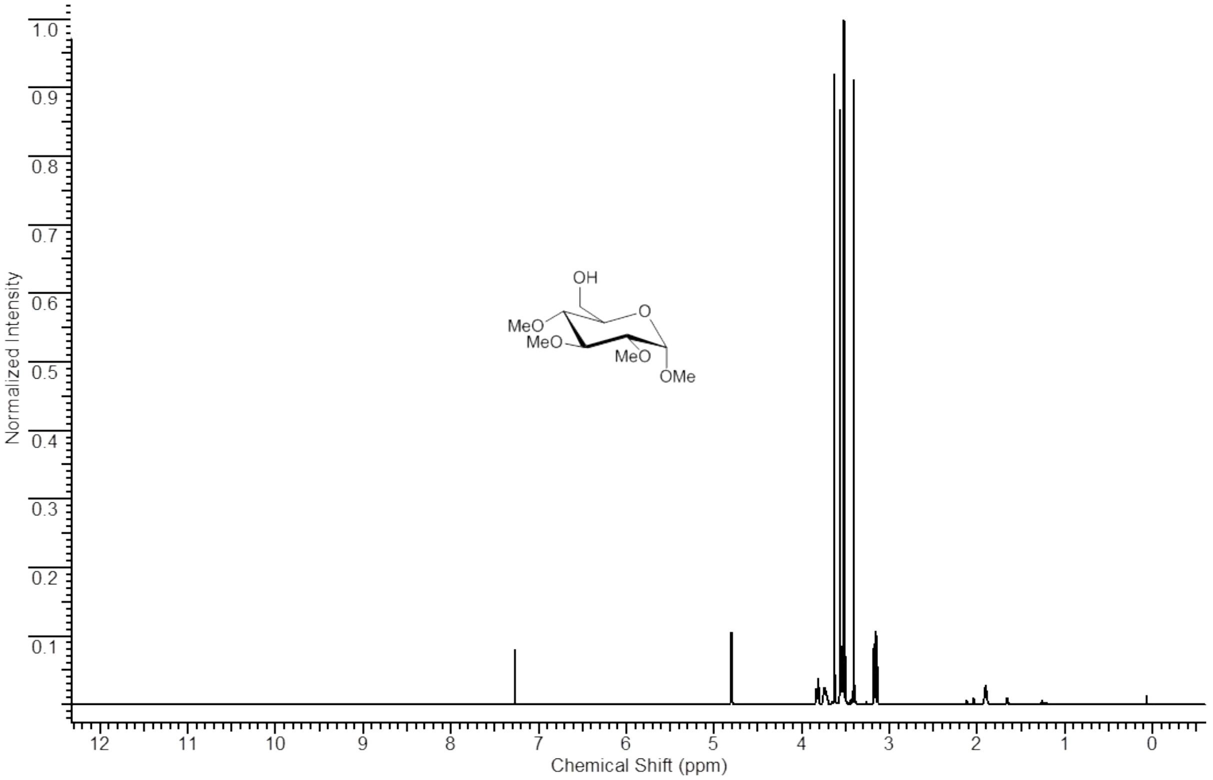

Figure 62: ^{1}H spectrum of compound **8d** (500 MHz, $CDCl_3$).

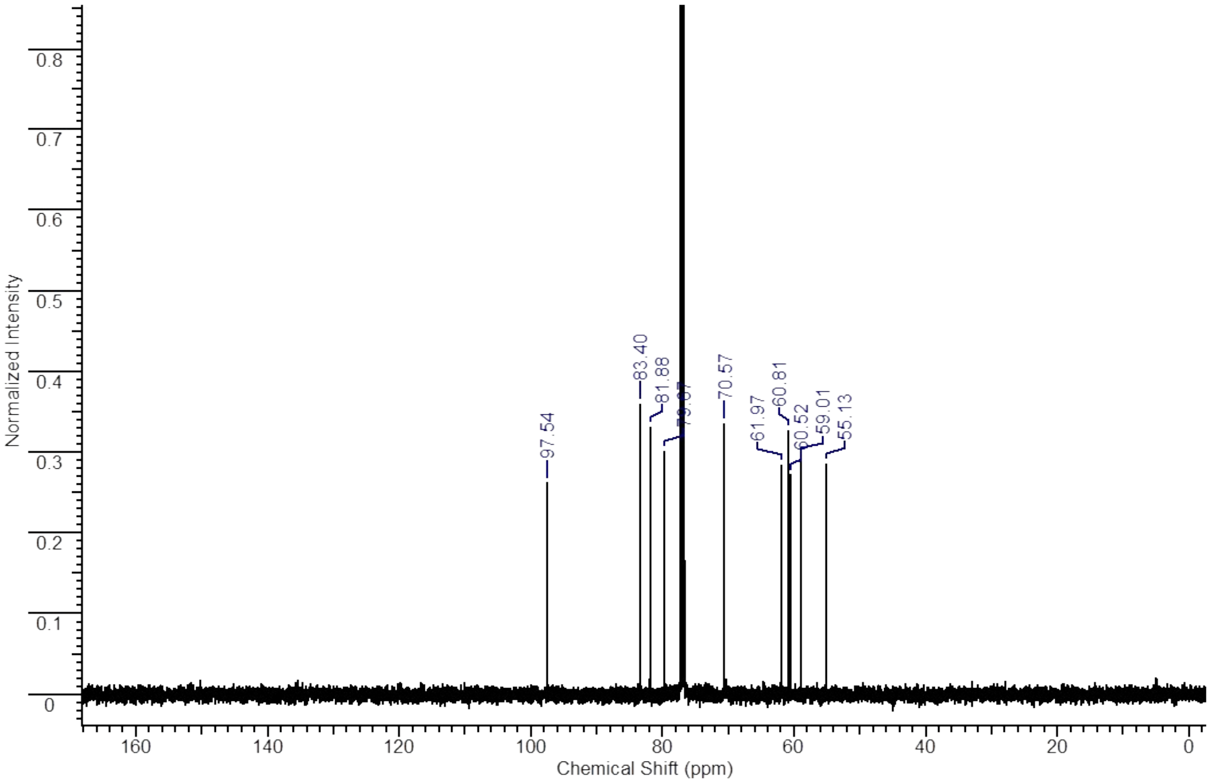

Figure 63: ^{13}C spectrum of compound **8d** (125 MHz, $CDCl_3$).

Methyl-2,3,4-*O*-ethyl-β-D-glucopyranoside 5h

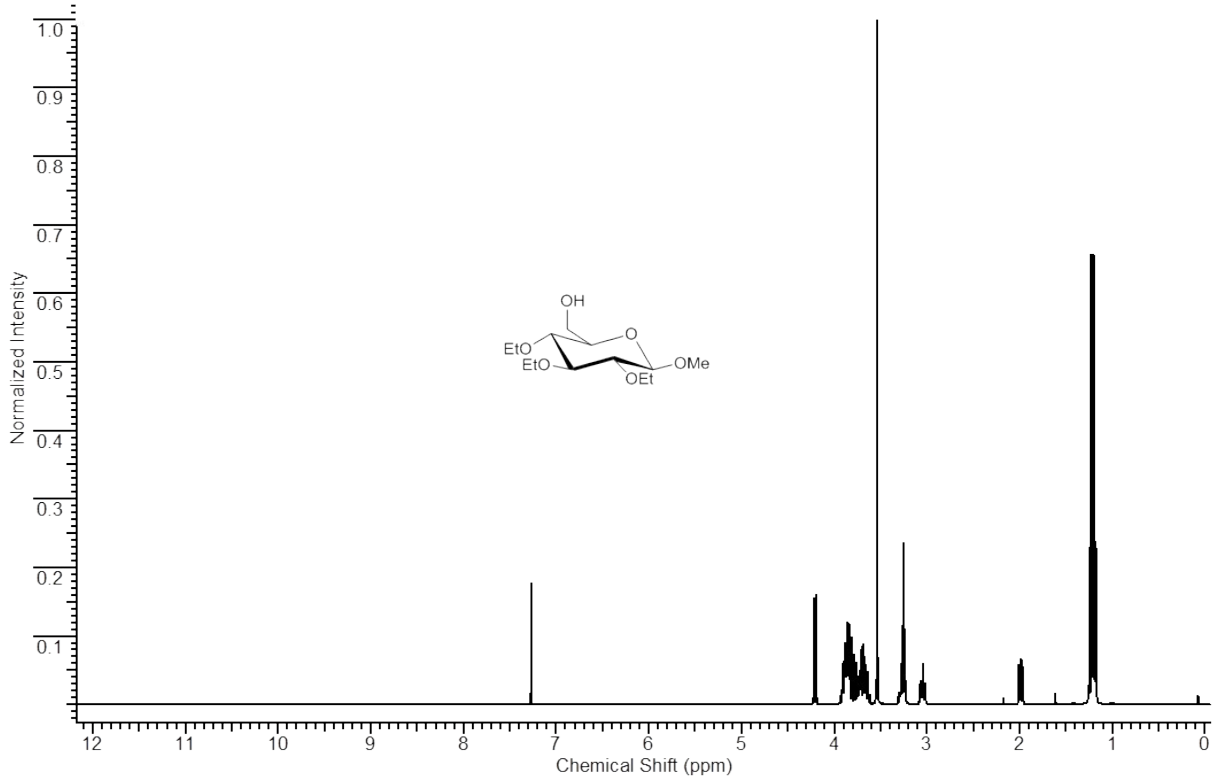

Figure 64: ^{1}H spectrum of compound **5h** (300 MHz, $CDCl_3$).

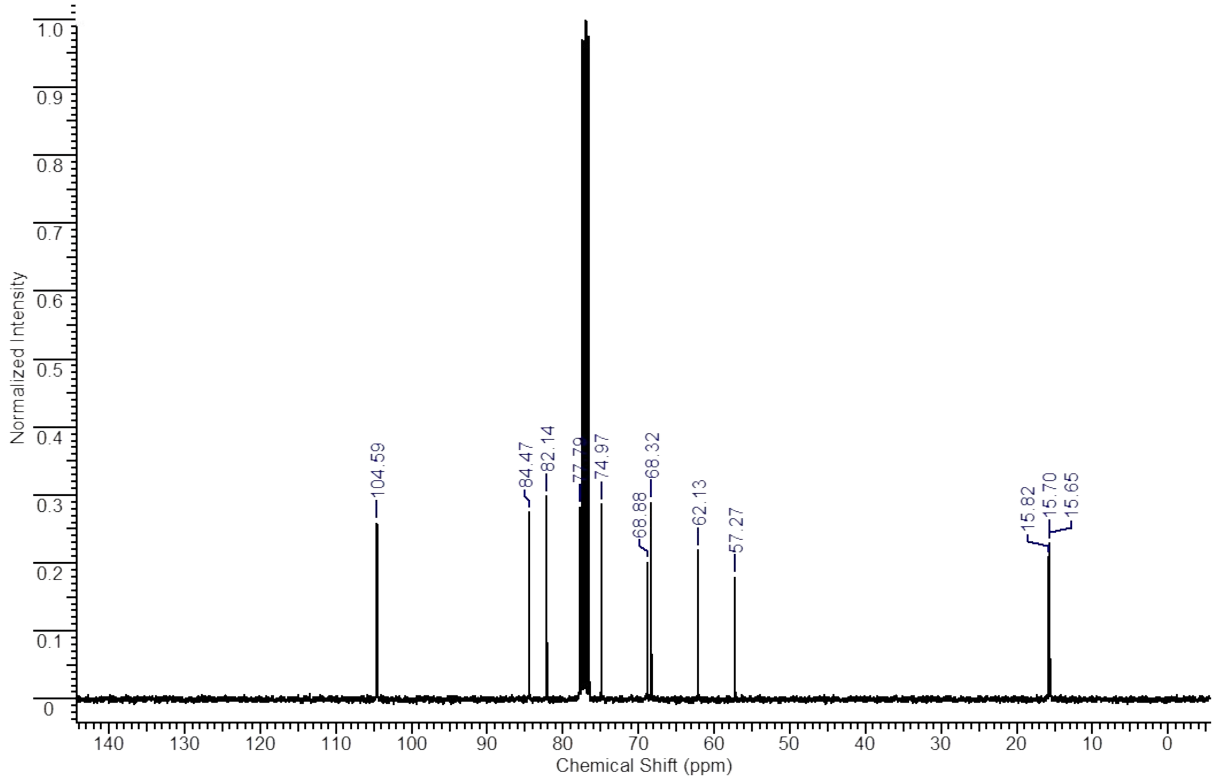

Figure 65: ^{13}C spectrum of compound **5h** (75 MHz, $CDCl_3$).
